# NLRP4 unlocks an NK/macrophages-centered ecosystem to suppress non-small cell lung cancer

**DOI:** 10.1186/s40364-025-00756-4

**Published:** 2025-03-14

**Authors:** Zhouwenli Meng, Jian Li, Hui Wang, Zhengqi Cao, Wenqing Lu, Xiaomin Niu, Yi Yang, Ziming Li, Ying Wang, Shun Lu

**Affiliations:** 1 Shanghai Lung Cancer Center, Shanghai Chest Hospital, Shanghai Jiao Tong University School of Medicine, Shanghai, 200030 P. R. China; 2 Shanghai Institute of Immunology, Department of Immunology and Microbiology, Key Laboratory of Cell Differentiation and Apoptosis of Chinese Ministry of Education, Shanghai Jiao Tong University School of Medicine, Shanghai, 200025 P. R. China

**Keywords:** NLRP4, Natural killer cell, Macrophages, Chemokines, PP2A

## Abstract

**Background:**

Tumor immune evasion extends beyond T cells, affecting innate immune elements like natural killer cells (NK) and macrophages within the tumor-immune microenvironment (TIME). Nevertheless, translational strategies to trigger collaboration of NK cells and macrophages to initiate sufficient anti-tumor cytoxicity remain scarce and are urgently needed.

**Methods:**

In this study, TCGA datasets was used to confirm the prognosis value of the expression level of NLR family pyrin domain containing 4 (NLRP4) in NSCLC and the tumor tissues microarray was used to further check its clinical-relevance at protein-level. Subsequently, a tumor cell line with stable NLRP4 overexpression was established and subcutaneous tumor models in C57BL/6J mice were used to validate the anti-tumor characteristics of NLRP4. After analyzing the tumor microenvironment using flow cytometry and multiplex immunofluorescence, we further validated our findings through co-culture transwell assays and TCGA analysis. Utilizing bulk-RNA sequencing, proteomics, and mass spectrometry of mouse tumor tissues, we innovatively identified the downstream pathways of NLRP4 and verified them through co-immunoprecipitation (co-IP) and Western blot (WB) experiments.

**Results:**

NLRP4 could trigger a distinct anti-tumor ecosystem organized by TIGIT^+^TNFA^+^ NK and iNOS^+^ M1 in lung cancer, discovered in TCGA analysis and verified in murine model. NLRP4-eco exerted tumor-suppression capacity through chemokine reprogramming including CCL5 and CXCL2. Meanwhile, the cytoxicity of NK could be facilitated by iNOS^+^M1. Mechanistically, NLRP4 stimulated PI3K/Akt-NF-kB axis through suppression of the activity of PP2A. Besides, knockdown of CCL5 and blockade of CXCL2-CXCR2 axis abolished chemotaxis of TIGIT^+^TNFA^+^ NK and iNOS^+^ M1 respectively, as well as for LB-100, a PP2A inhibitor.

**Conclusion:**

Altogether, we delineated NLRP4’s unexplored facets and discovered an NLRP4-driven anti-tumor ecosystem composed of TIGIT^+^TNFA^+^ NK and iNOS^+^ M1. Finally, targeting PP2A by its inhibitor successfully mimicked the anti-tumor capacity of the overexpression of NLRP4.

**Supplementary Information:**

The online version contains supplementary material available at 10.1186/s40364-025-00756-4.

## Background

Combination strategies, encompassing nanomedicine [1], CAR-T [2] and normalization immunotherapies [3], have yielded substantial enhancements to constrained response rates of anti-PD1/L1 blockade [4] (ICIs-com). Owing to their antigen-specific potency, T cells have garnered significant attention in ICIs-com, with a primary focus on the tumor immune microenvironment (TIME) T cells reactivation [5]. However, the duality of T cells behavior introduces complexity and reflexivity. Notably, Zou et al. have illuminated how IFNγ from TIME T cells could drive hyperprogression through the PKM2-β-catenin axis [6]. Concurrently, as an evolving plan B, efforts are being directed toward liberating alternate immune subpopulations residing within TIME, such as fibroblasts [1, 7], neutrophils [8, 9], macrophages [10, 11], and NK cells [12, 13]. However, ICIs-com that targeted immune subtypes beyond T cells are still at dawn [14, 15].

Besides, such paradigm highlighted the importance of considering TIME as a co-evolving multicellular ecosystem. Luca et al. thus delineated 10 distinct tumor ecosystems composed of 69 cell states, transcending conventional categorization of TIME into simplistic cell subtypes in TIME [16]. Wang et al. further proposed that each tumor ecosystem finally attained its stable configuration through a unique stromal network, a concept defined as the “lock-and-key” model [17]. Viewing each tumor ecosystem as a distinct “lock”, mechanisms serving as the respective “keys” warrant comprehensive investigation. Chemokines hold the potential to act as these “keys”. A case in point is the rescue of ICIs resistance through CCL5 delivery, which facilitated the recruitment of CD49a^+^ CXCR6^+^ NK cells, thereby stimulating collaborative cDC1-CD8 interaction [18]. Additionally, the overriding of SPP1^+^ macrophages by CXCL9^+^ macrophages contributed to an anti-tumor milieu [19]. However, impacts of chemokines are intricate, influenced by divergent sources and targets, as well as inter-tumor heterogeneity. For instance, while CXCL9 secreted by senescent endothelial cells accelerated breast cancer aggressiveness [20], macrophage-derived CXCL9 exhibited a positive correlation with T cells expansion in T-cell lymphomas [21]. Therefore, cancer-type specific exploration is imperative for comprehending chemokine-mediated “unlocking” of the TIME ecosystem [22].

Non-small cell lung cancer (NSCLC) has reaped substantial benefits from ICIs-com, with the majority of which centered around T cells reactivation [4]. Conversely, a comprehensive understanding of the roles played by other innate immune constituents of NSCLC ecosystem, including macrophages and NK cells [23], remains an area of insufficient exploration. Notably, Heymach et al. have substantiated that the dysfunctionality of NK and CD8^+^ T cells, driven by IL6, underpins the acquired resistance to tyrosine kinase inhibitors (TKIs) in NSCLC [24]. Furthermore, Denardo et al. have unveiled the pivotal role of macrophages in lung cancer through their impediment of infiltration of CD8^+^ T cells [25]. Nevertheless, given that focuses of these notable discoveries primarily on T cells-centered ecosystems, it remains unclear whether collaboration of NK cells and macrophages can independently initiate sufficient anti-tumor cytoxicity, in the absence of T cells involvement.

Within this research, we presented NLR family pyrin domain containing 4 (NLRP4) as a potential “key” capable of unlocking the NK and macrophages-centered TIME ecosystem. NLRP4 is a member of the NLRPs protein family. Takeshita et al. have identified NLRP4 as a negative regulator of autophagosome maturation, attributed to its interaction with BECN1 [26]. Additionally, Wang et al. have demonstrated that NLRP4 knockdown could activate IRF3 by disrupting K48-linked ubiquitination of TBK1 [27]. Despite these insights, its role within cancer biology, particularly in the context of NSCLC, remains largely unexplored.

Herein we demonstrated that NLRP4 overexpression (NLRP4-OE) served as the “key” to trigger a distinct anti-tumor ecosystem marked by the presence of TIGIT^+^TNFA^+^ NK cells and iNOS^+^ M1-polarised macrophages, a beneficial “lock” not reliant upon T cells (NLRP4-eco). Mechanistically, NLRP4 directly interacted with PP2A to initialize the PI3K/Akt-NF-kB axis to reprogram chemokines secretome, resulting in heightened expression of CCL5 and CXCL2. Furthermore, chemotactic effects of CXCL2-CXCR2 and CCL5-CCR5 axes specifically recruited TIGIT^+^TNFA^+^ NK cells and iNOS^+^ M1 respectively. In the era characterized by overwhelming exploration of T cells, we presented an alternative avenue with focus on TIME ecosystem centered around NK cells and macrophages unlocked by NLRP4, holding significant promises for next-generation TIME-based immunotherapies.

## Methods

### Genomics analysis for NLRP4

LUAD (Lung Adenocarcinoma (TCGA, PanCancer Atlas)) and LUSC (Lung Squamous Cell Carcinoma (CPTAC, Cell 2021); Lung Squamous Cell Carcinoma (TCGA, Firehose Legacy); Lung Squamous Cell Carcinoma (TCGA, Nature 2012); Lung Squamous Cell Carcinoma (TCGA, PanCancer Atlas)) data and clinical information were downloaded using the cBioPortal (API) in R (4.3.0) [28]. Genomic profiling used mRNA expression z-scores relative to all samples (log RNA Seq V2 RSEM) to deduce mRNA expression. Non-redundant samples were picked out before survival and mRNA DEGs analysis. Tumor mutation burden (TMB) data was obtained from the TCGA database (TCGA: https://www.cancer.gov/tcga) and the “maftools” R package was used to analyze the TMB. Additionally, survival analysis was performed using GEO datasets and other curated datasets mentioned in Table S1.

### Analysis of immune infiltration in tumors

The immune scores for the NLRP4-high and NLRP4-low groups were evaluated using the ‘ESTIMATE’ package [29]. Single-sample gene-set enrichment analysis (ssGSEA) was adopted to immune cells proportion deduction from TCGA LUNG using the “GSVA” R package [30]. Immune signatures from previously published studies were utilized for this analysis [31].

### Animal experiments

C57BL/6 mice or nude mice, aged between 6 and 8 weeks, were housed in a specific pathogen-free environment, with equal representation of both female and male mice. Based on existing literature, we employed varying cell inoculation numbers tailored to specific cell types (detailed in Table S14), administering them into the right flank of either C57BL/6 mice or nude mice via subcutaneous injection. To generate tumor growth curves, we monitored the survival of mice for a duration ranging from 17 days (LLC, MC38, B16, CMT167) to 27 days (SJTU1601). Tumor dimensions were assessed periodically using calipers, and tumor volume was determined by applying the formula: (length × width^2) / 2. To profile TME, spleen and TDLN by mIF, mFC, bulk-RNA-seq, we sacrificed mice at a specific timepoint (earlier than day17) when tumor volumes were comparable across different experimental groups, to mitigate confounding factors such as tumor-volume-induced systemic inflammation (detailed in corresponding figure ligands). In addition, we adopted an additional strategy to validate our findings in cases where identifying a suitable timepoint to sacrifice all mice simultaneously proved challenging: we sacrificed them at various timepoints when tumor volumes were comparable for different treatment groups (detailed in Table S14). For the implementation of anti-PD-L1 blockade, anti-PD-L1 and PBS were administered intraperitoneally (i.p.) at a dose of 200 µg per mouse on day 7 following tumor cell inoculation. Subsequently, this treatment was repeated every 3 days throughout the experiment duration. To facilitate the depletion of CD8, NK, and CSF1R cells, specific antibodies were employed. Anti-CD8 antibodies were administered at a dose of 60 µg per mouse, anti-NK1.1 antibodies at 100 µg per mouse, and anti-CSF1R antibodies at 200 µg per mouse, all delivered intraperitoneally (i.p.). These antibody treatments were initiated prior to tumor implantation (days–2) and continued with twice-weekly administrations for the duration of the study. In order to dissect paramount immune cell-types specifically in mice tissues, we employed the Seq-ImmuCC method as described in previous literature [32].

### mIF and IHC

Human NSCLC samples were obtained with approval from the Ethics Committee of Shanghai Chest Hospital. All patients provided informed consent. Animal tumor specimens were processed as previously described and received approval from the Animal Ethics Committee of Shanghai Chest Hospital. Human samples were incorporated into tissue microarrays (TMA) with clinical information outlined in Table S12-S13. They underwent multiplex immunofluorescence (mIF), immunohistochemistry (IHC), or organoid culture procedures according to the methodology detailed elsewhere (Method). Tumor specimens from animals or humans were fixed in 10% neutral-buffered formalin (Sigma-Aldrich) for 24 h. They were then transitioned to 70% ethanol or frozen in optimal cutting temperature (OCT) compound. For immunofluorescence, paraffin-embedded slides were subjected to antigen retrieval by boiling in sodium citrate buffer (pH 6.0) for 15 min. After blocking with a solution of 1% BSA and 5% FCS in PBS, slides were incubated overnight at 4 °C with primary antibodies. Subsequent to three PBS washes, the slides were exposed to secondary antibodies in the dark at room temperature for 1 h and mounted with DAPI. Immunohistochemistry staining for tumor cell smears involved fixing them with formalin and washing in PBS. Following a 5-minute permeabilization step, the smears were blocked for 30 min. They were then incubated for 1 h with primary antibody, followed by detection using horseradish peroxidase (HRP)-conjugated secondary antibody. Comprehensive information regarding the antibodies used in these analyses is available in Table S2.

### CCK-8 assay

To perform CCK-8 assays, cells were plated into 96-well plates at a density of 1 × 10^3^ cells per well and cultured in complete medium. Subsequently, CCK-8 assays were conducted following the manufacturer’s instructions (DOJINDO; cat: CK04).

### Tumor interstitial fluid (TIF) collection

TIF were collected and processed as has been described [33]. Briefly speaking, we adopted a non-enzymatically dissociation method to exclude any influence from chain reactions caused by enzymes, and better preserve integrity of surface expressing antigen epitopes of TIL or TIM [34]. For all tumor bulks dissected, tumor weights were recorded and then minced entirely. Every sample was handled with the same amount of PBS (1 ml/each tumor), thus the protein concentration of each TIF was in positive correlation with the respective tumor weight. After quantification of protein concentration by PiercePCA (Thermo; cat: 23225), TIF were processed as described in Luminex and ELISA methods.

### Culture of BMDMs

For the cultivation of BMDMs, bone marrow cells were extracted from the femur’s bone marrow cavity of mice. These cells were then passed through a 70-µm cell strainer (Millipore) and subsequently centrifuged. The collected bone marrow cells underwent treatment with ACK lysis buffer (Gibco; cat: A1049201) to eliminate red blood cells. Afterward, these cells were differentiated into macrophages via a 7-day culture period in DMEM supplemented with 10% FBS and 50 ng ml^− 1^ M-CSF. Verification of macrophage differentiation was conducted using flow cytometry.

### Transwell-based co-culture

BMDMs were isolated as described previously. For the chemokine assay, 5 × 10^5^ spleen cells or BMDMs were seeded into the top chambers with an 8.0-µm PET membrane and 1 × 10^5^ tumor cells were seeded into the bottom wells in complete medium with the addition of 200 ng ml^− 1^ CXCL2/CCL5/CXCL10/CXCL14 recombinant murine proteins or mCXCL2 antibody (anti-CXCL2). For CXCR1/CXCR2/CXCR4 blockade, CXCR1/CXCR2/CXCR4 antibody was incubated with cells in upper chambers for 6 h. Afterwards, antibodies were washed for 3 times to exclude potential blockade effects upon tumor cells. After 48 h co-culture, all cells in the lower chamber were collected for flow cytometry analysis. The concentration and category numbers of proteins and antibodies mentioned above were listed in Table S4. Flow cytometry (Fortessa X20) was used to count the numbers and frequencies of migrated cells.

### 2D coculture of tumor cells with murine spleen cells

Murine spleens were mechanically grinded and filtered through 70 μm cell strainers. After red cell lysis by ACK lysis buffer and washing for 3 times, 2 × 10^6^ spleen cells were incubated with 1 × 10^5^ tumor cells for 6 hours or 24 h in RPMI, 10% FBS and 1% penicillin/streptomycin at 37 °C. Then cells were harvested and analyzed by flow cytometry.

### Flow cytometry and high-dimensional analysis

All reagents and antibodies for flow cytometric analysis were purchased from Biolegend/BD. For surface staining, cells were harvested and washed by FACS (PBS containing 2% FBS) 2 times, then stained in FACS with antibodies at 1:400 dilution on ice for 30 min in the dark and then washed twice with FACS prior to Flow Cytometry analysis. For intracellular cytokines staining, cells were harvested and washed 2 times with FACS, permeabilized and stained with Intracellular Fixation & Permeabilization Buffer set (Biolegend; cat: 426803) according to the manufacturer’s protocol. Stained cells were washed 2 times with FACS. For intranuclear cytokines, cells were harvested and washed for 2 times with FACS, permeabilized and stained with transcription factor staining buffer set (Biolegend, Cat: 424401) according to the manufacturer’s protocol. Stained cells were washed 2 times with FACS before analysis. BD LSR Fortessa X20 (Beckton Dickson) for FACS data collection. Information pertaining to all antibodies used in this study is shown in Table S2. Data analysis was carried out using FlowJo (v10.0.7). For high-dimensional analysis, Toolkits are listed below (https://www.flowjo.com/exchange/#/): *DownSample* to normalize fluctuations in collected cell numbers in different samples; *UMAP and FitSNE* used for dimensionality reduction, with the later in major lineage immune subpopulations gating and the former in subsequent more delicate immune subpopulations exploration; *ClusterExplorer* for definition, visualization and discrimination of specific subpopulations across different conditions.

### FACS sorting of M1 macrophages and NK cells

Inspired by CD107A and GZMA/B internalization in CD8^+^ T cells [35, 36], which has been adopted for dyeing during organoid-T-cells coculture [37], we developed analogous method to mark macrophages with NOS2 and TNFA without 2% PFA fixation or penetration. TNFA and NOS2 both underwent a recycle process from membrane-bound form to cytosolic form finally to secretion form [38–41], thus, we incubated polarized macrophages with respect antibodies overnight (> 12 h) with specific stimulators (Table S7). Different antibodies targeting TNFA and NOS2 (Table S7) were tested for their internalization efficacy. Afterwards, cells were immediately sorted within 2 h to avoid rapid secretion.

### Co-culture of NK cells, macrophages and tumor cells in transwell plates

NK cells were FACS sorted, while iNOS^+^ M1, TNFA^+^ M1 and M2 were polarized and sorted as described above. Macrophages were polarized 48 h before the transwell assay (reagents in Table S7). At the experiment day of co-culture, the murine spleen was handled as described above, with NK cells sorted as CD45^+^NK1.1^+^CD3^−^ cells. Then polarized macrophages were sorted (iNOS^+^ M1: CD11B^+^F4/80^+^CD80^+^ NOS2^+^ cells; TNFA^+^ M1: CD11B^+^F4/80^+^CD80^+^ TNFA^+^ cells; M2: CD11B^+^F4/80^+^CD80^−^ cells). The tumor cells were placed in the lower chamber of the transwell plate (12-well plate, 1 × 10^5^ per well). According to specific group settings, macrophages alone or with NK cells were seeded on the upper chamber. The absolute cell number in the upper chamber was 5 × 10^5^ (macrophages alone: 5 × 10^5^ macrophages; with NK cells: 2.5 × 10^5^ macrophages and 2.5 × 10^5^ NK cells). After 48 h co-culture, all cells (including tumor cells) in the lower chamber were collected and analyzed by flow cytometry.

### RNA sequencing

The entire RNA was withdrawn from both mouse tissues and tumor with the TRIzol Reagent (Invitrogen), according to what the manufacture explained. The status of the RNA was assessed using a devise named Agilent 2100 (purchased from Agilent Technologies). mRNA was subsequently enriched using Oligo(dT) beads. mRNA was then enriched, and fragmented into shorter fragments utilizing the so-called buffer of fragmentation and afterwards reverse transcribed into cDNA employing the One-Step PrimeScript™ RT-PCR Kit (Takara, cat: RR064A). Then we performed purification of ligation reaction with AMPure XP Beads (1.0X). After completing electrophoresis of agarose gel and amplification of polymerase chain reaction (PCR), we finished the sequencing of cDNA library using Illumina Novaseq6000. Low-quality reads were further filtered by fastp [42] and HISAT2.2.4 [43] with “-rna-strandness RF” and other parameters set as a default was used for alignment with the reference genome. Then the quantification of gene abundance was calculated by StringTie v1.3.1 [44] in a reference-based approach.

### Gene set enrichment analysis (GSEA)

For GSEA, all differentially expressed genes (DEGs) arranged by log2FC (calculated by the R package “EdgeR” [45]) were used. The HALLMARK and IMMUNESIGDB genesets from the Molecular Signatures Database (MSigDB; v7.3) [46] were utilized as input for the GSEA process, performed using the GSEA function from the R package “clusterprofiler“ [47]. Gene sets with a p.adjust value smaller compared to 0.05 were considered to have significance and were visualized through “ggplot2” R package.

### Reverse Transcription-Polymerase chain reaction (RT-PCR)

Total RNA was isolated from cells using the TRIzol (Sigma) reagent and then reverse transcribed into complementary DNA (cDNA) using a reverse transcription kit (Takara). Quantitative real-time PCR was conducted using SYBR Green PCR Master Mix (Takara) along with specific primers. The reactions were carried out on an ABI 7500 System (Applied Biosystems). The resulting cDNA was amplified through real-time PCR, and the data were analyzed using the 2^−ΔΔCt^ method. The amplification primers used are provided in Table S8.

### Luminex

Chemokines in the supernatant of cells and TIF was detected by Luminex (Luminex, X-200) according to the manufacturer’s instructions (R&D systems; cat: LXSAMSM-07; Bio-rad; cat: 12009159). The panel for luminex assay was listed in Table S4.

### ELISA

Cell culture supernatants and TIF were collected and enzyme-linked immunosorbent assay (ELISA) was performed using Mouse CXCL14/BRAK ELISA Kit (Colorimetric; Novus; cat: NBP2-70015), following protocol’s instructions. For sequential-HYGITIC or transwell-based co-culture as described, cell supernatants were collected, together with cell lyses from sorted BMDM or NK, to perform ELISA using Mouse/Rat Visfatin ELISA Kit (Abcam; cat: ab267799), with extracellular NAMPT (eNAMPT) representing NAMPT in supernatants and intracellular NAMPT (iNAMPT) representing NAMPT in cell lyses.

### Seahorse assay

M1omega and NKomega were sorted as iNOS^+^ M1 and TIGIT^+^ NK cells, respectively, following the procedures outlined in the *CISS* (Method). The XF96 Extracellular Flux Analyzer (Seahorse Bioscience) was used for measuring the basal extracellular acidification rate (ECAR) and oxygen consumption rate (OCAR) after seeding cells at 2 × 10^4^ cells/well. In groups “NKomega_FK866” and “M1omega_FK866,” FK866 (100 µM) was utilized. After an overnight incubation at 37 °C to allow M1omega cells to adhere and NKomega cells to settle, the growth media was replaced with Seahorse Phenol Red-free DMEM. For glycolysis assessment, ECAR was quantified, followed by the supplement of rotenone (0.5 mM) and antimycin A (0.5 mM) to block mitochondrial complex 1 and 3 activity, separately. Afterwards, 2-DG (50 mM) was added. To scrutinize respiration of mitochondrial, we measured basal oxygen consumption, followed by the sequential supplement of oligomycin (2 mM), protonophore carbonyl cyanide-4-(trifluoromethoxy)-phenylhydrazone (FCCP) (1 mM), and 0.5 mM rotenone together with 0.5 mM antimycin A.

### Killing assays

INOS^+^M1, TNFA^+^M1, and M2 macrophages were sorted as described in *CISS* method, whereas NK cells were isolated from mouse spleen cells using EasySep™ Mouse NK Cell Enrichment Kit (Stemcell; cat: 19755). These sorted cells were then incubated with tumor cells either alone (transwell-based co-culture) or in the presence of immune cells. Floating cells and cells in tumoroids were collected and stained using the fluorescein isothiocyanate (FITC) Annexin V Apoptosis Detection Kit I (BD; cat: 556547). Tumor cells were gated by negative gating of CD45 using the Attune NxT acoustic focusing flow cytometer.

### RNA interference

HANBIO (Shanghai, China) provided murine CCL5-siRNA and non-silencing scrambled control (SCR) siRNA. The specific siRNA sequences were as follows: CCL5-siRNA: 5’-UCUUGAUUCUGACCCUGUAUATT-3’, 3’-UAUACAGGGUCAGAAUCAAGATT-5’. SCR-siRNA: 5’-UUCUCCGAACGUGUCACGUTT-3’, 3’-ACGUGACACGUUCGGAGAATT-5’. Serum-free medium was adopted for cells transfection period and we transfected with a mixture of siRNA and Lipofectamine RNAiMAX Reagent (Invitrogen; cat: 13778150), according to the manufacturer’s explanation. After cells have been incubated in 37℃ for exact 5 h, afterwards we used fresh culture medium (10% FBS) to exchange for serum-free medium, and the desired treatment was conducted.

### Western blot analysis

For immunoblotting analysis, cell lysis were acquired through incubation in RIPA buffer (epizyme; cat: PC101) containing protease inhibitor (epizyme; cat: GRF101) and phosphatase inhibitor (epizyme; cat: GRF102) on ice for 30 min. Lysates were subsequently centrifuged at 15,000 × rpm at 4 °C for 20 min. Protein concentrations were determined using the BCA kit (Beyotime Biotechnology), and the denaturalization of lysates were completed in loading buffer. Sodium dodecyl sulfate-polyacrylamide gel electrophoresis (SDS-PAGE) were used to separate equal amounts of lysates, and afterwards proteins were transferred onto polyvinylidene fluoride (PVDF) membranes (Millipore). Blocking was performed using 5% Bovine Serum Albumin (BSA) at room temperature (RT) for 1 h. Membranes were then incubated overnight at 4 °C with primary antibodies, followed by incubation with HRP-conjugated secondary antibodies at RT for 1 h. Chemiluminescence was detected using an Amersham Imager 600 (GE) with western HRP Substrate (Tanon). Antibody details used for western blotting are listed in Table S2.

### Cell culture

Lewis lung carcinoma (LLC), Mouse Colon Cancer Cells (MC38) and HEK293T cells were procured from the Shanghai Institute for Biological Sciences Chinese Academy of Sciences (Shanghai, China). The complete medium was formulated as follows: Dulbecco’s modified eagle medium (DMEM) with high glucose (Hyclone; cat: SH30027.02) for LLC and HEK293T. All cell lines were authenticated and maintained in a humidified 5% CO2 incubator at 37 °C. LY294002 (LY; Beyotime Biotechnology; cat: S1737) was added to the cell culture medium at concentrations of 20 and 50 µM L-1 for 4 h, and Okadaic acid (OA; Beyotime Biotechnology; cat: S1786) was added at concentrations of 0.05 and 0.1 µM L-1 for 4 h before cell harvesting.

### Stable transfection or knockdown of NLRP4

- The gene ID of mouse NLRP4 is 446,099. Plasmid of NLRP4-OE is as follows:
- pcSLenti-EF1-mCherry-P2A-Puro-CMV-NIrp4e-3xFLAG-WPRE;
- Plasmid of NLRP4-NCOE is as follows:
- pcSLenti-EF1-mCherry-P2A-Puro-CMV-MCS-3xFLAG-WPRE;
- Plasmid of NLRP4-KD is as follows:
- pSLenti-U6-shRNA(NIrp4e)-CMV-mCherry-F2A-Puro-WPRE;
- Plasmid of NLRP4-NCKD is as follows:
- pSLenti-U6-shRNA(NC)-CMV-mCherry-F2A-Puro-WPRE;

Vector backbones were purchased from obiosh (Shanghai, China). We designed three different NLRP4-KD vectors with different shRNAs, and conducted following experiments in parallel. Through RT-PCR and WB verification, this NLRP4-KD shRNA target region was finally chosen for stable transfection: CAAAGATCAAGATTCCCTTAAGCAGAAATTTACCCAGGATG (448–468). DH5α competent cells were purchase from Thermo Fisher (cat: EC0112), and utilized for lentivirus plasmids as well as helper plasmids (pSPAX2 (Addgene, cat: 12260), pMD2.G (Addgene, cat: 12259)) amplification. PureLink™ HiPure Plasmid Miniprep Kit (Thermo Fisher, cat: K210002) was adopted for plasmids extraction (> 200ng/µl, A260/280 between 1.8 and 1.9). HEK293T cells before passage 4 were employed for lentivirus packaging. Helper plasmids, along with *nlrp4e* or its control, were co-transfected into HET293T cells. DMEM + 1%FBS complete medium was used. After 16 h, medium was half-changed, and after an additional 48 h, supernatant from HEK293T cells was collected, ultra-centrifugated (13000 g/min, 90 min). Viruses titer (TU/mL) = number of cells * percentage of mCherry (viable cells) * MOI * virus dilution factor * 10^3. For all C57BL/6J cell-lines (LLC, MC38), we all used the same NLRP4-OE and NLRP4-KD lentiviruses, as well as their control. 80 MOI was chosen for transfection. After 72 h, cells were collected for quantification of transfection efficacy (> 40%) via RT-PCR and WB. To establish stable transfected cell lines, 2 µg/ml puromycin (Beyotime Biotechnology; cat: ST551)) was added to the culture medium for 16–28 days until mCherry-positive cells exceeded 90%. Monoclonal cells were isolated using the limiting dilution cloning method, and stable transfected cell lines were passaged and expanded for downstream experiments.

To investigate into mechanisms of NLRP4-eco, we further constructed stable knockdown of CCL5, CXCL2 and AKT1 in NLRP4-OE LLC cell-line separately, using the same experiment pipeline as aforementioned above. To find out the responsible domain of NLRP4 for direct interaction with PP2A. We purchased mouse NLRP4 lentivirus and plasmids expressing different NLRP4 fragments (PYD, NOD, and LRR) from obiosh (Shanghai, China). We chose transient transfection here. LLC or HEK293T cells were transfected with plasmids using Lipofectamine 2000 (Invitrogen; cat: 11668019) following the manufacturer’s instructions. Cells were harvested for downstream experiments after incubation at 37 °C for 24–48 h.

### Immunoprecipitation assays

The flag antibody (Beyotime Biotechnology; cat: AF2852) or mouse IgA isotype control was incubated with 4 mg of protein lysate collected from LLC OE cells and incubated rotating overnight at 4 °C. Pierce™ Protein A/G Magnetic Beads (Thermo Fisher; cat: 88802) were washed three times using RIPA buffer supplemented with protease inhibitor and phosphatase inhibitor, then incubated with antigen-antibody complex at 4 °C for 2 h. The immunoprecipitants were eluted from the beads with 1X SDS loading buffer and incubation at 95 °C for 10 min, separated on 10% SDS-PAGE gels, and immunoblotted with the indicated antibodies or verified by LC/MS.

### Label-free proteomics

The LC-MS/MS detection system employed for label-free proteomic analysis featured a nanoflow high-performance liquid chromatograph (HPLC) instrument: Easy-nLC1000 System (Thermo Fisher). In a concise synopsis of the process, a peptide mixture amounting to 1–2 µg was expertly resolved in buffer A (0.1% formic acid (FA)) and meticulously loaded onto a 2-cm self-packed trap column (100-µm inner diameter, ReproSil-Pur C18-AQ, 1.9 μm; Dr Maisch). The peptides were separated on a 75-µm-inner-diameter column with a length of 20 cm (ReproSil-Pur C18-AQ, 1.9 μm; Dr Maisch), spanning an orchestrated 78-minute gradient (buffer A, 0.1% FA in water; buffer B, 0.1% FA in ACN) at a meticulously controlled flow rate of 200 nl/min. Operating in the Data Dependent Acquisition (DDA) mode, the Orbitrap Fusion was finely tuned to perform a full mass spectrometry survey scan. The target value was set at 5 × 105, with scans spanning from 350 to 1,600 m/z. Subsequent analysis of the mass spectrometry data involved Proteome Discoverer (V2.4) software, featuring the Percolator database search algorithm. To enable a targeted search, the reference protein database employed was UniProt_Mouse_20190908.fasta, aligning with the mouse proteome in UniProt. To ensure robustness and accuracy, a filtering step was implemented, with a threshold of ≥ 0.05 for the Maximum Delta Cn and Maximum Rank of peptide-spectrum matches (PSMs). Proteins meeting stringent criteria for differential expression were defined based on two factors: (1) fold change between groups exceeding or equaling 2 or falling below or equaling 0.5 (i.e., 1/1.2); and (2) a requisite of at least 2 unique peptides supporting the identification.

### Liquid chromatography tandem mass spectrometry analysis (LC-MS/MS)

LC-MS/MS encompassed stages: bleaching, alkyl reduction, enzymatic digestion, peptide extraction, and desalting. The reconstitution of the processed samples was accomplished through a 0.1% FA solution, maintaining sample integrity while preparing for separation. Employing an EASY-nLC 1000 system (Thermo Fisher), the subsequent separation was executed using an analytical column, fine-tuned for a meticulous flow rate of 500 nL/min. To ensure optimal spectral acquisition, the full scan resolution was set at 60,000 (FWHM), extending over a mass-to-charge ratio (m/z) range of 350–1800. During the acquisition process, automatic gain control (AGC) values were diligently set to 4 × 10^5^, coupled with a scan window of 0.7 m/z and a maximum ion injection time of 50 ms. To facilitate controlled fragmentation for subsequent analysis, higher-energy collisional dissociation (HCD) was deployed. A collision energy setting of 30% ensured precise fragmentation of ions. The resolution for secondary mass spectrometry was adeptly set at 15,000, with an AGC value of 1 × 10^5^ and a maximum ion injection time of 22 ms. Analysis was orchestrated by Proteome Discoverer (V2.4) software.

### Structure predictions of point-mutated proteins

PPP2CB and NLRP4 *fasta* sequences were obtained from NCBI database (https://www.ncbi.nlm.nih.gov/protein/). Point mutations information of NLRP4 of NSCLC patients were collected from cBioPortal database (https://www.cbioportal.org/). Point-mutated *fasta* sequences were uploaded to I-TASSER-MTD [48], and results were visualized by ChimeraX (version: 1.6.1). Annotated PDB files derived were then submitted for further analysis by ScanNet and Cluspro2.0.

### Predictions of protein binding sites

PDB files of wildtype PPP2CB and NLRP4 were obtained from Alphaphold (https://alphafold.ebi.ac.uk/entry/) with accession number P62714 and Q96MN2. Point-mutated PDB files were acquired from I-TASSER-MTD as aforementioned. After docking analysis through clusPro2.0 [49], docked protein complex PPP2CB-NLRP4 (chain1-chain2) were also downloaded as PDB files. All these PDB files were uploaded to ScanNet [50] for deep-learning based binding sites prediction. For PPP2CB and NLRP4, binding site probability scores were ranked by amino acids at each domain, and those beyond 0.3 as well as participated in docking, were grouped to become hotspots. Afterwards, for docked protein complex PPP2CB-PP2A, binding sites at each chain were matched to respective protein, and those with no corresponding binding sites at the interface were excluded.

### Single-cell dataset collection

Datasets were collected and curated from published datasets. The accession numbers were listed in Table S3.

### NSCLC NK/M cells atlas construction

The R package Seurat (version 4.3.0) [51] was used for the dataset integration. Briefly, we performed normalization using SCTransform with an additional flag vst.flavor="v2” to invoke the v2 regularization, then performed integration using pearson residuals [52]. After that, we used the standard analysis pipeline of the R package Seurat. To extract NK and M, we got immune cells by PTPRC expression, and here we should mention that myeloid cells have lower level of PTPTRC as has been described [53]. We negatively selected T cells by CD3E, CD8A and CD4. Afterwards, NKG7 and GNLY were used to examine purity of NK cells after RunUMAP and FindClusters methods (Seurat), and CD68 for myeloid cells. Different expression genes (DEGs) were accessed through the FindMarkers functions in Seurat with the “MAST” method for each cluster. Significant marker genes, as determined by their log2 fold change (log2FC) greater than 1 and adjusted p-value (p.adjust) less than 0.05, were used to make annotations for each cell type based on relevant information available in the literature (Table S5, S9).

### Monocle2-based trajectory analysis

To investigate the trajectory of NK cells, an unsupervised pseudotime analysis was conducted using Monocle2 [54]. Besides, we developed a pipeline to screen for targeted NK subsets based upon Monocle2: progressively-narrowing monocle2 (n-monocle). Basically, we would re-run monocle pipeline until we achieved the resolution demand based on biological information, and herein we have re-run for three times using a reversed graph embedding algorithm (DDRTree). Next, the BEAM function was employed to identify key genes involved in the differentiation of branches. The default parameters were used for this analysis. To visualize the identified genes, Complexheatmap [55] was utilized. We embedded 11 NK subsets for the first-round monocle, and identified clear differentiation trajectories for each subset. In accordance with published results, ITGA4_NK and CX3CR1_NK laid at rather earlier pseudotime coordinates (state5), followed by TCF7_CD27_NK (state3). CCL4L1_NK tended to distracted into rather terminal states (state2 and state1), and TIGIT_NK were dominant by state1. To be mentioned, FOSL2_NK was the only NK subset with evenly distributed states across the trajectory, strongly indicating that it was a heterogeneous subset, as could be verified in UMAP embeddings either. Besides, we could not find definitive prerequisite knowledge in literature regarding this subset. On the contrary, as aforementioned, CX3CR1_NK, TCF7_CD27_NK, CCL4L1_NK, KLRF1_NK and TIGIT_NK were relatively more well-characterized, biologically-informative and dominant by no more than 2 states in the first-round monocle analysis, thus we included them into the second-round monocle analysis further. Astonishingly, CCL5 manifested the most expression at terminally differentiated state5, falling into a similar trajectory as TIGIT. Besides, KLRF1 and CX3CR1 were both considered as markers for NK cells at earlier states, as has been illuminated here (state1). Although GZMA and TIGIT were all enriched at state5, state5 was hybridized by cells from TIGIT_NK and CCL4L1_NK, which explained the reason that the rather-gentle up-regulation trend of TIGIT. Indeed, monocle2 was somewhat limited by its low resolution for too many subsets embedded [54], thus we chose CCL4L1_NK and TIGIT_NK to re-run monocle2 for the third-round, in an attempt to distinguish them and dissect their characteristics.

### DEG and pathway analysis

Pathway analysis was performed by “clusterprofiler” with all DEGs and “C7”, “C8”, “REACTOME” and “KEGG” gene sets obtained from MSigDB. Significant pathways were visualized by ggplot2.

### Expression signature score

The “irGSEA” method was utilized to calculate the signature scores of different states of tumor-associated macrophages (TAMs) originating from published articles (Table S6) with “singscore” methods.

### Cell-cell communication

We used NicheNet [56] to predict ligand-receptor interactions that might drive TIGIT_NK and INOS2_mono/macro gene expression changes. The default parameters were set to predict cell–cell interactions.

## Results

### Favorable OS detonated by NLRP4 overexpression in NSCLC

**Fig. 1:**
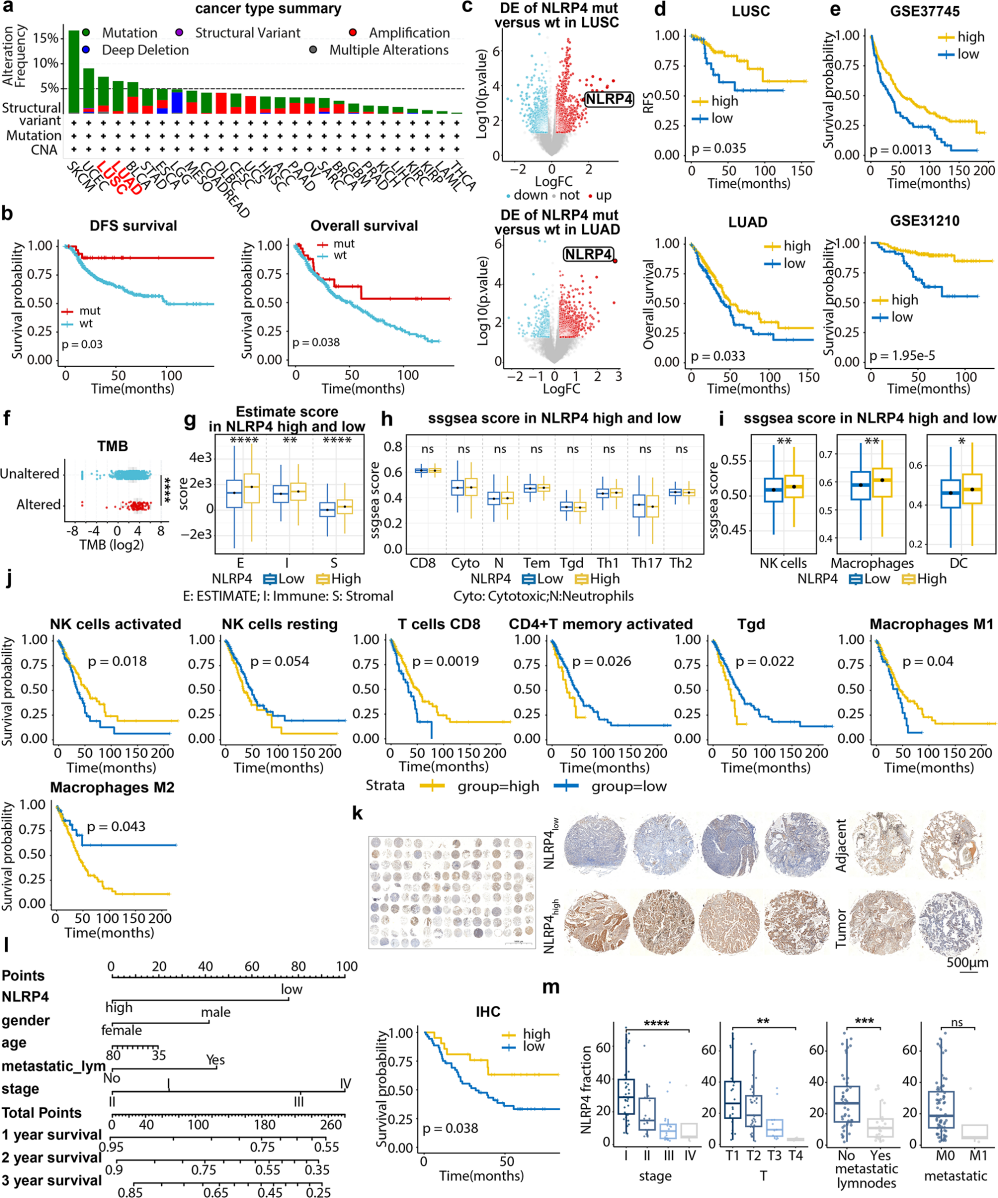
NLRP4 overexpression correlates with improved OS and specialized hot TIME in NSCLC The data were produced utilizing publicly-available or in-house human specimens. **a**. Cancer type summary for NLRP4 alterations. **b**. Kaplan-Meier curves analysis showing that NLRP4 alteration associated with better DFS in TCGA_LUSC (upper, HR (95%CI): 0.3 (0.16, 0.58)) and better OS in TCGA_LUAD cohorts (lower, HR (95%CI): 0.65 (0.46, 0.91)). **c**. Volcano plot showing the differentially expressed genes (DEGs) in NLRP4 alteration versus WT in TCGA LUSC (upper) and TCGA LUAD cohort (lower). **d**. Survival curve showing that the expression of NLRP4 was associated with better RFS in stage I/II TCGA LUSC (HR (95%CI): 0.43 (0.17, 1.11)) and better OS in TCGA LUAD cohort (HR (95%CI): 0.72 (0.53, 0.99)). **e**. Survival curve showing that the expression of NLRP4 associated with better OS in the NSCLC cohort. **f**. Boxplots showing TMB in NLRP4 altered group and unaltered group. p-value calculated by t-tests and corrected by Bonferroni. **g**. Immune infiltration score calculated by Estimate in NLRP4 high and low expression group. **h**. Ssgsea scores of adaptive immune cells in NLRP4 high and low expression group. **i**. Ssgsea scores of innate immune cells in NLRP4 high and low expression group. **j**. Survival curve of immune populations deconvoluted by CYBERSORT in TCGA LUAD cohort (from left to right: HR(95%CI): 0.66 (0.45, 0.94); 1.43 (0.97, 2.11); 0.54 (0.33, 0.87); 1.72 (0.95, 3.14); 1.71 (0.97, 3.03); 0.6 (0.34, 1.07); 1.99 (1.18, 3.34)). **k**. Immunohistochemistry (IHC) staining results of human NSCLC tissue microarray (NSCLC-TMA) for NLRP4. **l**. Forest plot of NSCLC-TMA and survival curve showing the association of score of NLRP4’s expression with better OS (HR (95%CI): 0.44 (0.22, 0.84)). **m**. Fraction of NLRP4 in patients from different clinical stages and TNM stages. Data represent mean ± SEM; ns *p* > 0.05, *p < 0.05, **p < 0.01, ***p < 0.001,and ****p < 0.0001 from unpaired Student’s t-test or one-way ANOVA. The p-value of Kaplan-Meier curves was determined by log-rank test (b, d, e, j, l).

We screened genomic alterations in TCGA-LUNG for genes with > 5% alterations, and conducted disease-free survival (DFS) analysis for clinically-relevant candidate genes, within which NLRP4 (6.93% in 1053 cases) was selected. NSCLC appeared to be the third most frequent cancer-type for NLRP4 genomic alterations, among which mutations (5.89%) were dominant (Fig. 1a). Evaluating separately for LUAD and LUSC, alterations in NLRP4 were associated with better DFS and overall survival (OS) respectively (Fig. 1b).

We then query whether mutations in NLRP4 were gain-of-function or deleterious. NLRP4 genomic mutations were correlated with over-expression in mRNA level either in LUAD or LUSC (Fig. 1c). Higher NLRP4 was relevant to improved OS in LUAD and relapse-free survival (RFS) in stageI/II LUSC (Fig. 1d, S1a). We used a set of GEO-based NSCLC datasets for further validation, and a consistent better OS was depicted (Fig. 1e, S1b). We constructed a NSCLC-specimen microarray (NSCLC-TMA) and further checked its clinical-relevance at protein-level (Fig. 1k). NLRP4 protein emerged as a favorable factor to OS as well (Fig. 1l), and exhibited more expression in stageI/II patients (Fig. 1m). Besides, patients with no lymphnode or distant metastases displayed augmented NLRP4 expression. Although no pronounced disparity was discerned between tumor and adjacent-tissue (Fig.S1c), a notable distinction was detected between stage III/IV specimens and corresponding adjacent-tissue (Fig. S1d), alluding to the distinctive clinical implications of NLRP4.

Afterwards, we profiled putative TMB quantification in NLRP4-altered samples. TMB pretended to be significantly up-regulated in NLRP4-altered group (Fig. 1f). Ricciuti et al. have proved that higher TMB was correlated with a “hotter” TIME in NSCLC [57]. Additionally, *estimate* score has commonly been adopted to portrait the extent of overall immune infiltration [29]. Intriguingly, elevated NLRP4 was linked to a “hotter” immune contexture (Fig. 1g). Subsequently, we set out to identify the related immune cells subtypes. T cells are responsible for up-leading anti-tumor capacity in “hot” TIME [57]. However, neither CD8^+^ T cells or any CD4^+^ T cells subtypes showed differences (Fig. 1h). Strikingly, NK cells, macrophages as well as dendritic cells (DC cells) pretended to be enriched in NLRP4-high group (Fig. 1i). Besides, except for CD8^+^ T cells, activated NK cells as well as M1-polarized macrophages were linked with better OS in TCGA-LUAD (Fig. 1j). In conclusion, we uncovered a specific TIME ecosystem correlated with higher NLRP4 expression in NSCLC (NLRP4-eco), which was linked with better OS.

### NLRP4-triggered an idiographic ecosystem composed of NK and M1 macrophages

**Fig. 2:**
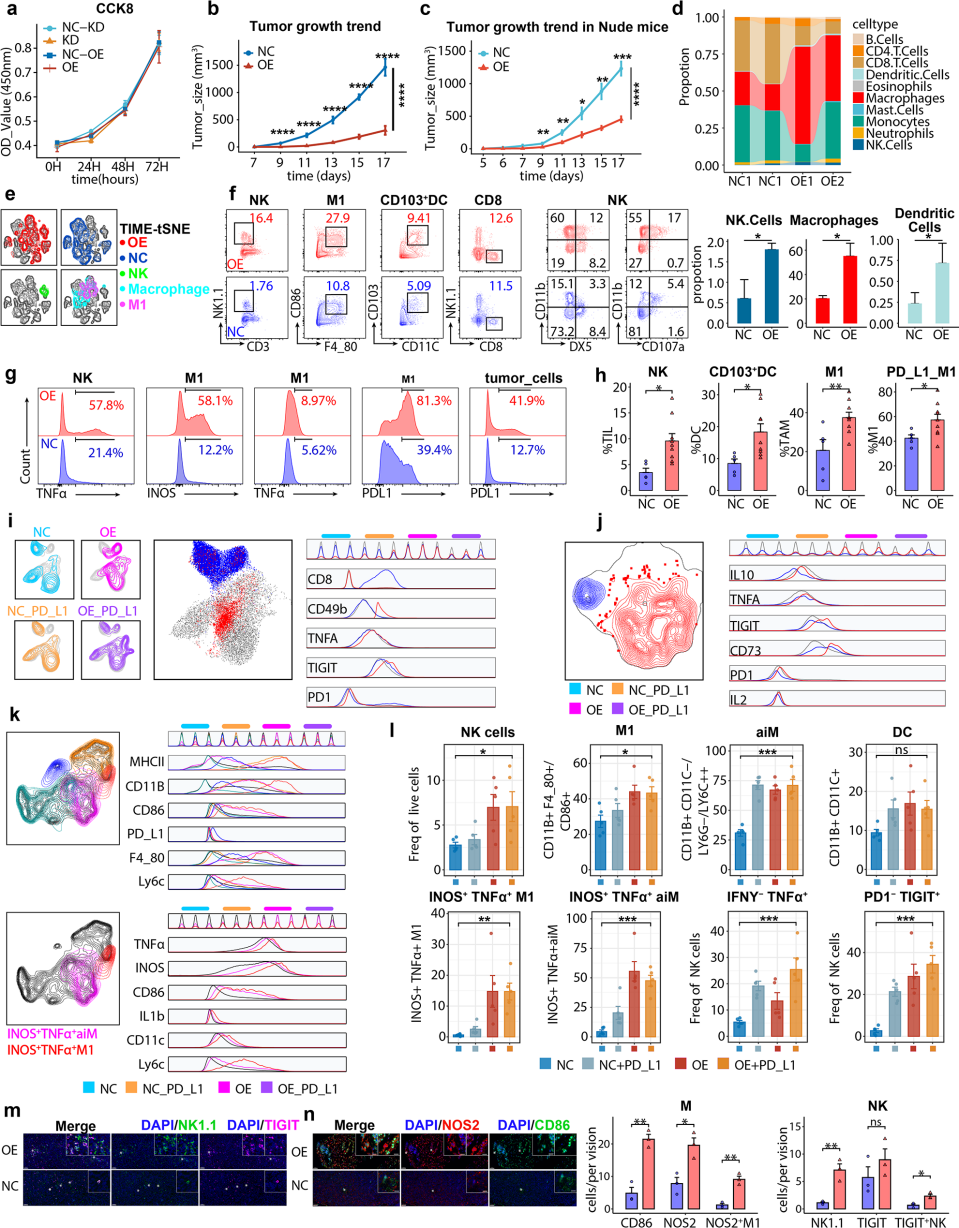
NLRP4 overexpression promotes the infiltration of iNOS^+^ M1 and TIGIT^+^TNFA^+^NK cells into the TIME The data were produced utilizing in-house murine specimens. **a**. Cell growth curve of NLRP4-KD, NLRP4-NCKD, NLPR4-NCOE, NLPR4-OE. (*n* = 9) **b**. Growth of LLC NLRP4-NCOE and NLRP4-OE in immunocompetent C57BL/6 mice. (*n* = 5–8 mice per group, representative of three independent experiments). **c**. Growth of LLC NLRP4-NCOE and NLRP4-OE in nude mice. (*n* = 5–8 mice per group, representative of three independent experiments). **d**. Proportions of immune populations deconvoluted from bulk-RNA seq of tumor tissues. **e**. tSNE plot showing TIME in NLRP4-OE and NLRP4-NCOE. **f**. Frequency of intratumoral NK cells, M1, CD103^+^DC and CD8^+^ T cells from mice bearing NLRP4-NC or NLRP4-OE LLC. Data was pooled from three independent experiments. **g**. TNFA, INOS, PDL1 levels on immune populations of mice bearing NLRP4-NC or NLRP4-OE LLC. Data was pooled from three independent experiments. **h**. Quantification of intertumoral NK cells, M1, CD103^+^DC and PDL1^+^M1. Data was pooled from three independent experiments. **i**. UMAP plot showing CD8 and NK’s distribution (left) and functional markers (right). **j**. UMAP plot showing NK’s distribution (left) and functional markers (right). **k**. UMAP plot showing tumor-infiltrating myeloid-cells distribution (left) and functional markers (right). **l**. Quantification of intertumoral NK cells, M1, aiM, DC and their TNFA, IFNY and INOS expression from mice bearing NLRP4-NC or NLRP4-OE LLC treated by PD-L1 or not (*n* = 5 mice per group, pooled from three independent experiments). **m-n**. Representative example of NLRP4-NC and NLRP4-OE tumors. Tumor staining by multiplexed IHC shows the spatial distributions of NOS2^+^ macrophage (m) and TIGIT ^+^ NK (n). Images are representative of at least three images for individual mice. DAPI, 4,6-diamidino-2-phenylindole. Data represent mean ± SEM; ns *p* > 0.05, *p < 0.05, **p < 0.01, ***p < 0.001,and ****p < 0.0001 from unpaired Student’s t-test, one-way ANOVA and two-way ANOVA followed by Tukey’s HSD post - hoc test for pairwise comparisons. Tumor growth was assessed using two-way ANOVA test.

We hypothesized that NLRP4-eco was exclusively mediated by innate immune subpopulations (NK and macrophages), independently of T cells. Firstly, neither NLRP4 overexpression (NLRP4-OE) nor knockdown (NLRP4-KD) in Lewis lung carcinoma (LLC) affected cell growth velocity proved by CCK-8 assay (Fig. 2a), and on the contrary, NLRP4-OE led to significant tumor shrinking in immunocompetent C57BL/6 mice (Fig. 2b, Fig. S2b), underpinning immune infiltration responsible for NLRP4-OE anti-tumor capacity. Furthermore, NLRP4-OE retained its phenotype in nude mice as well (Fig. 2c), thus demonstrating innate immune cells fully sufficient for mediating anti-tumor effects of NLRP4. For dissecting specific subpopulations, we conducted deconvolution analysis by seq-ImmunCC on bulk RNA-seq of NLRP-OE tumor tissues. Macrophages, NK and DC dominated in murine NLRP4-OE, in accordance with TCGA-LUNG (Fig. 2d). Besides, tSNE analysis portraited a different immune landscape in NLRP4-OE, with up-regulated NK, M1 macrophages (M1) and CD103^+^ resident DC cells (Fig. 2d-e). Surprisingly, CD8^+^ T cells have not manifested any variation (Fig. 2e, Fig. S2c), a finding further corroborated by mIF analysis and single-cell RNA sequencing (Fig. S3a-c, Fig. S4j). Since CD103^+^ DC were considered as mediator rather than executioner of anti-tumor cytotoxicity [58], we further examined functional behavior of M1 and NK. Except for up-regulation of TNFα within NK and M1, iNOS, a classical marker of M1 secretome also outstood, accounting for 58.1% M1 in NLRP4-OE with merely 12.2% in NLRP4-NCOE (NC) (Fig. 2g).

Immuno-stimulators like bioengineered nanoparticles often synergized with anti-PD-L1 therapies through collateral enhancing expression of PD-L1 [59, 60]. We here observed significant up-regulation of PD-L1 on M1 as well as tumor cells (Fig. 2g-h, Fig. S2c), thus taking anti-PD-L1 as a potential combination strategy. Although PD-L1 blockade (αPD-L1) could not further fuel the anti-tumor capacity of NLRP4, we confirmed an outstanding tumor-suppressive capacity of NLRP4-eco, which was far more intensive than αPD-L1 reprogrammed TIME (αPD-L1-eco) (Fig. S2a). Taken a closer look at NLRP4-eco by *UMAP* analysis of TIL, we noticed that NK (CD49b^+^ cells) were induced in NLRP4-eco specifically, marked by enhanced expression of TNFA and TIGIT compared to CD8^+^ T cells (Fig. 2i). Besides, these TIGIT^+^TNFA^+^ NK cells grouped into a NLRP4-OE characteristic subpopulation (Fig. 2j, m-n). In the context of NK differentiation, NK in NLRP4-OE gathered into CD11b^+^ mature NK cells, with significant boosting of CD107α, suggesting enhanced degranulation (Fig. 2f, S2a). Compared with IFNγ, TNFA in NLRP4-OE tumor interstitial fluid (TIF) manifested consistent up-regulation regardless of CD8^+^ T cells deletion (Fig. S4g).

Additionally, *UMAP* analysis manifested significant INOS^+^TNFA^+^ M1 enrichment in NLRP4-eco either, which were unrepresented in NC (Fig. 2k-n). Accordingly, αPD-L1 had no effects upon neither NK nor iNOS^+^TNFA^+^ M1. Taken together, we considered TIGIT^+^TNFA^+^ NK and iNOS^+^ M1 as NLRP4-eco representative subpopulations (Fig. 2m-n). To verify whether NLRP4-eco was cancer-type conserved, we examined NLRP4-OE in MC38 cell-line, and confirmed the same anti-tumor capacity mediated by iNOS^+^ M1, TIGIT^+^TNFA^+^ NK and CD103^+^ DC (Fig. S2e-f), along with favorable OS (Fig. S2g), further emphasizing the unique landscape of NLRP4-eco across different cancer types.

### NLRP4-eco exhibiting TIME-restricted, CD8 T cells-independent features

Afterwards, we leveraged CD8^+^ T cells, NK cells and macrophages in-vivo depletion experiments separately to corroborate the mechanisms of NLRP4-eco. Apparently, NK cells and CD8^+^ T cells were both anti-tumor executioners in NC, with αCD8A and αNK1.1 significantly accelerating tumor growth, while αCSF1R retarded tumor growth conversely, which could be explained by immune-suppressive M2 macrophages dominancy in NC as has been deliberated [61]. Strikingly, αCD8A had no effects upon NLRP4-OE (Fig. S4a, S5a), while αNK1.1 and αCSF1R significantly dampened NLRP4-eco, supporting the leading roles of NK and M in NLRP4-eco except for CD8^+^ T cells (Figs. S4b-c, S5b-c).

Recently, systemic remodeling of macroenvironment has been considered indispensable for tumor-induced immunosuppression [62, 63]. Accordingly, we utilized multiplexed-flow-cytometry (mFC) to portrait immune composition in TIME, tumor-draining lymphnodes (TDLN) and spleen. Indeed, αCD8A depleted CD8^+^ T cells unanimously in TME, TDLN and spleen, but it did not abrogate NLRP-eco anti-tumor capacity (Fig. S4d, S5a). Furthermore, αCD8A had no effects upon M1 or their iNOS secretion in TIME, TDLN and spleen in the NLRP4-OE group (Fig. S5a). In contrast, αCD8A down-regulated iNOS^+^ M1 in TIME of NC (Fig. S6a). Additionally, αNK1.1 diminished NK cells in TIME, TDLN and spleen, without influencing CD8^+^ T cells (Fig. S6b). Strikingly, M1 and DC, another two characteristics of NLRP4-eco, both experienced shrinkage in TIME and TDLN (Fig. S5b), indicating a codependent, microenvironment-restricted network activated by NLRP4-OE, which was absent in NC (Fig. S6b). Coincidentally, αCSF1R reprogrammed NLRP4-eco to “cold” phenotype, with collateral damage to TIME NK, DC and even CD8^+^ T cells, but causing little disturbance to TDLN and spleen (Fig. S5c, S6c). Taken together, we concluded that NLRP4-eco was restricted to TIME dependent upon NK and M1, while instead CD8^+^ T cells occupied the cooperation with M1 in NC. We also noticed that while NK and M1 showed consistent up-regulation, M1 have shifted from TNFA secretion to iNOS production and NK gradually transitioned to TNFA^+^ TIGIT^+^ NK from spleen to TIME (Fig. S4e-f). Noticeably, iNOS^+^TNFA^−^M1 peaked, with NK fully functionalized in TIME compared to TDLN and spleen (Fig. S4g-i). We thus proposed that TIME-dependent chemotaxis of NK and M1 by NLRP-OE might be indispensable, and used NKomega and M1omega to represent them.

However, murine models do not always recapitulate human immune biology. Therefore, we further verified our findings in human specimens. Using autologous PBMC-humanized NSG mice (Fig. S7a), we found that NSCLC tumor tissues with higher NLRP4 expression (NLRP4-high, determined by IHC) showed significant tumor suppression in vivo (Fig. S7b-c). mFC analysis revealed that NKomega and M1omega were dominant in NLRP4-high tissues (Fig. S7d). Using organoid-PBMC co-culture platforms, we also found greater apoptosis in NLRP4-high organoids ex-vivo (Fig. S7e-f). Overall, we conclude that NLRP4 mediates an anti-tumor ecosystem in human tissues similar to that in mice.

### NKomega and M1omega in NLRP4-eco dominant by chemotaxes reprogramming

**Fig. 3:**
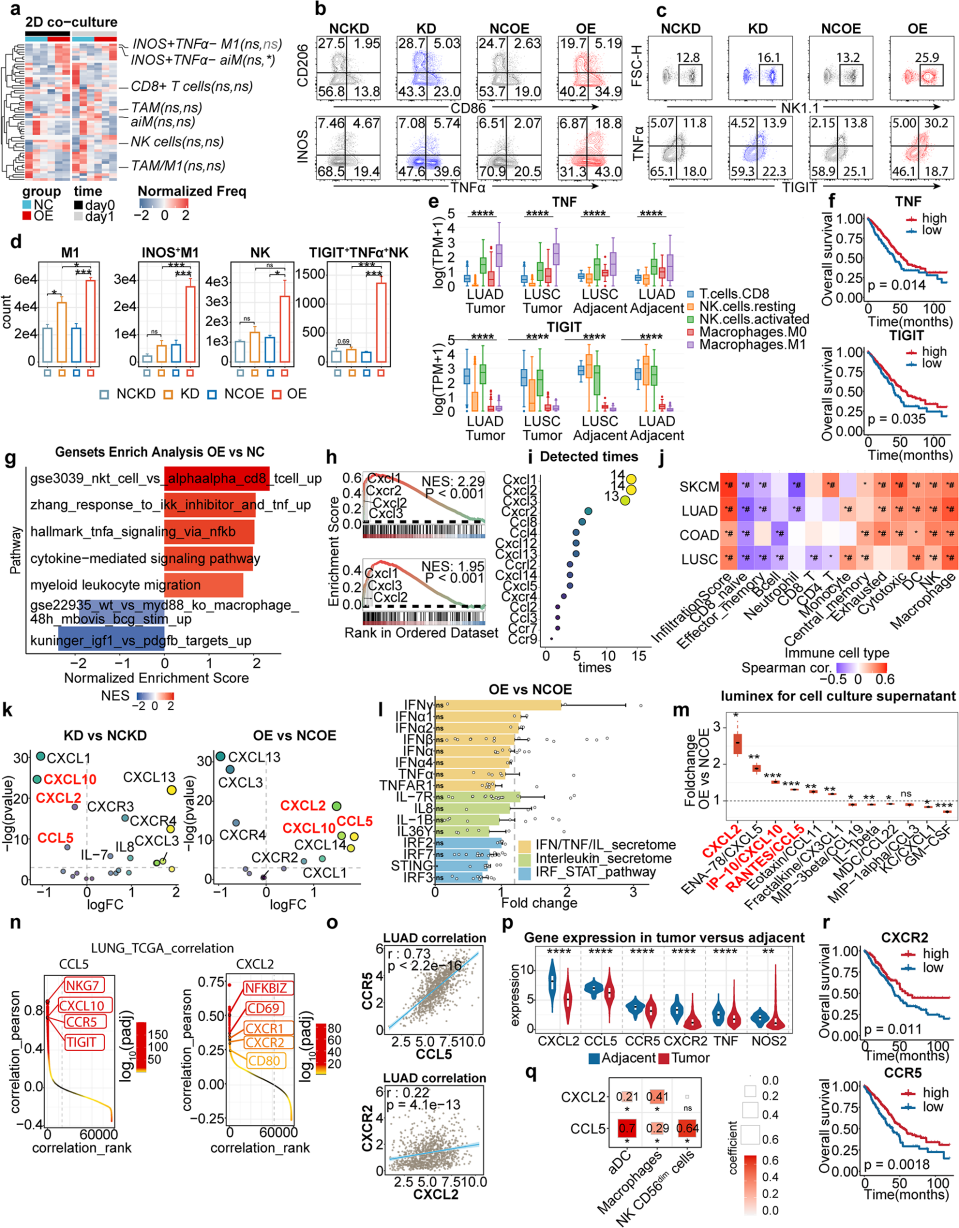
NLRP4 overexpression in tumor cells mediates the reprogramming of chemokines The data were produced utilizing publicly-available human specimens (**e-j**, **n-i**) or in-house murine specimens (**a-d**, **k-m**). For **a-d**, murine spleen cells isolated by Ficoll and LLC tumor cells (LLC) were used; for k-m, LLC were used. **a**. Heatmap plot showing immune populations’ proportion after co-culturing with NC or OE for 6 h (day0) or 24 h (day1). The left side in parentheses is p-value for day0 and the right for day1. **b-c**. Representative flow cytometric analysis of M1 and NK cells in transwell co-culture systems. **d**. Quantification of transitional M1, INOS^+^M1, NK and TIGIT^+^TNFA^+^NK in the lower chambers of transwell plates. (*n* = 3) **e**. The expression of TNF and TIGIT in different cell lineages from TCGA-LUSC and TCGA-LUAD. **f. **Survival curve showing that the expression of TNF and TIGIT associated with better OS in TCGA LUAD cohort (HR (95%CI): 0.69 (0.5, 0.95) for TIGIT, HR (95%CI): 0.73 (0.53, 0.99) for TNFα). **g-h**. Gene set enrichment analysis (GSEA) plot of significant pathways enriched in NLRP4-OE tumors, with the GSE3039 NKT CELL VS ALPHAALPHA CD8 TCELL UP pathway (upper in h) and Hallmark TNFA Signaling Via NFkB pathway (lower in h) listed out in h. **i**. Detected times of chemokines in GSEA analysis from (g). **j**. Heatmap showing Spearman correlations of chemokines from (i) with cell lineages in TCGA LUAD, LUSC, COAD and SKCM datasets. **k**. Volcano plot showing differently expressed genes in NLRP4-KD versus NLRP4-NCKD cells (left) and NLRP4-OE versus NLRP4-NCOE cells (right) detected by qPCR. Data was pooled from three independent experiments. **l**. Bar plot showing differently expressed genes in NLRP4-OE versus NLRP4-NCOE detected by qPCR. Data was pooled from three independent experiments. **m**. Boxplot showing the concentration of chemokines and cytokines in cell culture supernatant from NLRP-OE versus NLRP4-NCOE cell lines. **n**. Genes correlated to CXCL2 and CCL5 in TCGA LUNG. Genes were ranked by the Pearson correlation coefficient and colored by adjusted p-value. **o**. Relationship of CXCL2 with CXCR2 (upper) and CCL5 with CCR5 (lower) in TCGA-LUAD. P values were determined by a two-sided linear regression t-test. **p**. Violin plot showing expression of certain genes in tumor versus adjacent from TCGA-LUAD datasets. **q**. Relationship of CXCL2 and CCL5 with immune cell lineages in TCGA-LUNG. P values were determined by a two-sided linear regression t-test. l. Survival curve showing that the expression of CXCR2 and CCR5 associated with better OS in TCGA-LUAD cohort. Data represent mean ± SEM; ns *p* > 0.05, *p < 0.05, **p < 0.01, ***p < 0.001,and ****p < 0.0001 from unpaired Student’s t-test, one-way ANOVA. The p-value of Kaplan-Meier curves was determined by the log-rank test and those of gene enrichment analysis were calculated by R package GSEA

We next set out to dissect mechanisms behind NLRP4-OE anti-tumor capacity. We firstly questioned whether NLRP4-OE could stimulate NK or M1 proliferation and activation in a direct-contact manner. However, 2D coculture only captured moderate enrichment of INOS^+^TNFA^−^ M1/, and failed to recapitulate NK enhancement (Fig. 3a). We further utilized transwell-based co-culture system to thoroughly profile NLRP4-induced immune re-distribution. NK, M1 and CD103^+^ DC chemotaxis were exclusively induced by NLRP4-OE (Fig. 3b-d, S8a-b). More importantly, NKomega (TIGIT^+^TNFA^+^ NK) and M1omega (iNOS^+^ M1) exhibited as characteristic subpopulations within NLRP4-eco, with up to 7-fold enrichment.

Afterwards, we utilized deconvolution analysis in TCGA-LUNG [64] to clarify features of TIGIT and TNFA. Astonishingly, TIGIT and TNFA showed significant up-regulation on activated NK versus resting NK (Fig. 3e). Furthermore, TIGIT on activated NK possessed TIME-specific, CD8^+^ T-comparable features in TCGA-LUNG compared to adjacent tissue and other T-cells-lineage cells (Fig. S8c). When it came to macrophages, TNFA also manifested enhancement on M1 compared to M0 in a TIME-restricted manner (Fig. 3e). What’s more, NOS2 was revealed as marker of M1 versus M2 or TAN (Fig. S8d), and functioned as an activation hallmark compared to M0 or other major cytotoxic TIL in a TIME-specific way (Fig. S8e).

We lately explored tumor-intrinsic features responsible for NLRP4-eco fabrication. Through GSEA analysis, we identified MyD88-dependent Arg1-inhibition of NO [65] was down-regulated in NLRP4-OE, accompanied by M2-secreted IGF1 [66] vanishment (Fig. 3g-h). We further performed analysis to grasp high-frequency genes appeared in significantly enriched GSEA pathways, and generated genesets-variation-analysis (GSVA) score based upon them (chemo-sig). Astonishingly, CXCL1/2/3 along with CXCR2 predominantly occupied primacy (Fig. 3i). Besides, we observed a consistent and significant correlation between heightened infiltration of M1, NK and DC with chemo-sig across various cancer-types.

Afterwards, we screened for genes that up-regulated in NLRP4-OE but down-regulated in NLRP4-KD, in order to further narrow down obtained high-frequency genes above mentioned. Interestingly, CXCL2, but not CXCL1/3, stood out along with CCL5 and CXCL10 (Fig. 3k). Since NLRP4-KD has been reported to manipulate interferon/interleukins pathways (Table S10), we examined published downstream signaling mediators (IRF2/7, STING, STAT1/2, etc.) as well as other secretome molecules (TNFA, IL1B, IFNA, etc.) in NLRP4-OE, and there appeared to be no influences (Fig. 3l). Afterwards, we utilized Luminex assay to screen for any possible secretome modulations. Astonishingly, CXCL2, CCL5 and CXCL10 entered top four on protein level again (Fig. 3m), forcing us to consider them as core chemokines leveraging M1omega and NKomega.

We proceeded to examine TCGA-LUNG to investigate the chemokine receptor profiles. CXCR3, the CXCL10 receptor, was essential for T cells infiltration [67, 68], but lacked conclusive evidence of its expression on NK. Besides, correlation analysis revealed CCR5, instead of CXCR3, as the first highly-related receptor for CXCL10 (Fig. 3n-o). CXCL10 cooperated with CXCL9/11 to recruiting CD8^+^ T cells [68, 69], implicated by CD8A ranked before NKG7 (Fig. S8h). On the contrary, CCL5 was the major chemokine responsible for NK recruitment. However, CXCR1/2/4 showed similar correlation with CXCL2 (*r* = 0.34/0.22/0.29) (Fig. 3n-o, S8f), which have all been reported as its receptors [70, 71]. Meanwhile, CXCR2- CXCL2, along with CCR5-CCL5 obviously up-regulated in tumor-adjacent tissue (Fig. 3p), and they are linked with better OS (Fig. 3l, S8g), except for CXCL10 (Fig.S8h). Moreover, CXCL2 manifested biased inclination to macrophages in TCGA-LUNG, as well as for CCL5 to CD56^dim^ NK over CD8^+^ T cells (Fig. 3q, S8i). Consistently, CXCL2 correlation analysis demonstrated the prominent association of M1 markers (CD80, CD69).

Based on findings aforementioned, we formulated a hypothesis proposing the existence of two potential axes, CXCL2-CXCR1/2/4 and CCL5/CXCL10-CCR5, which might serve as crucial mediators in shaping the NLRP4-eco.

### Exclusive modulation of M1omega and NKomega by CXCL2 and CCL5 respectively

**Fig. 4:**
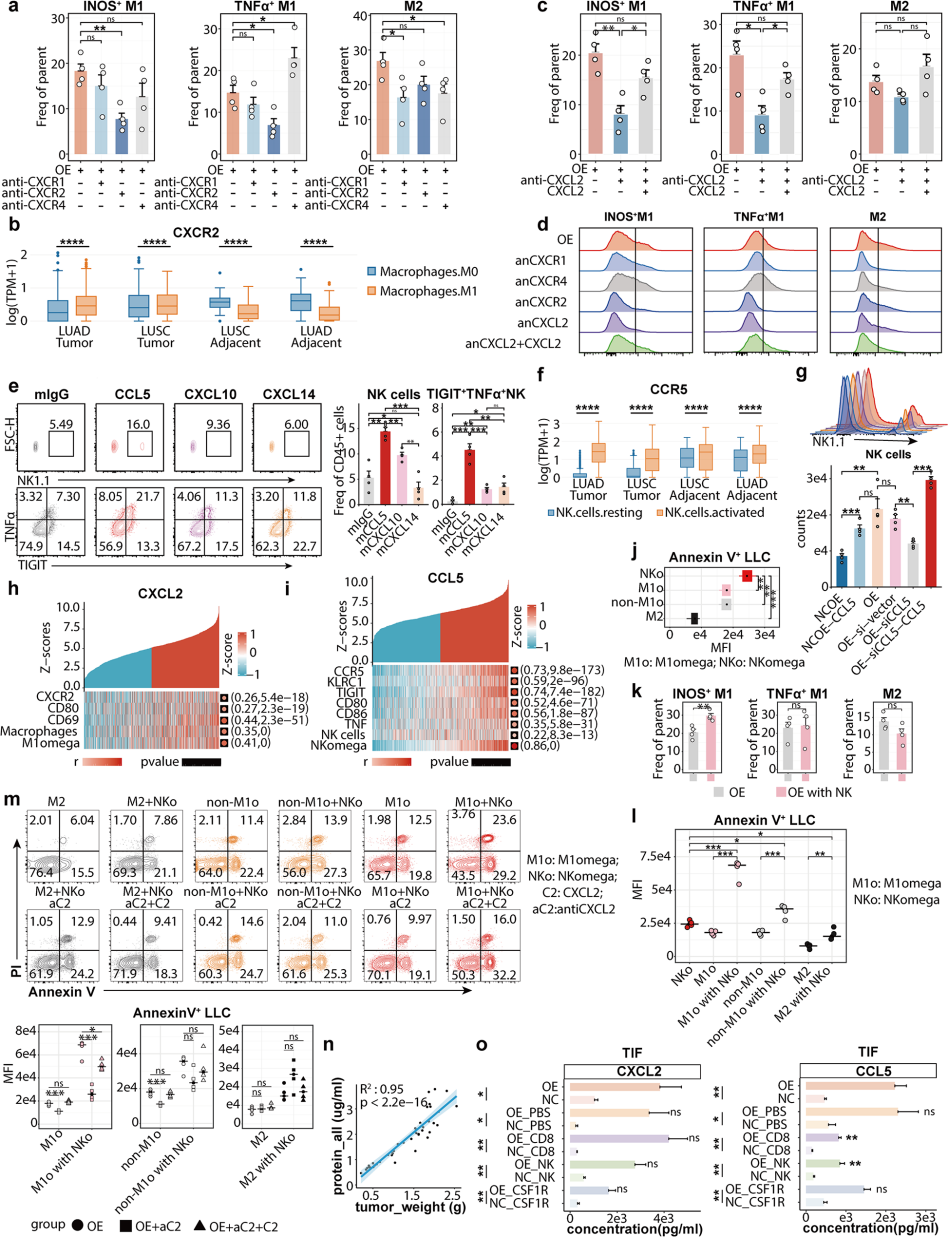
The synergistic effect between NKomega and M1omega depends upon the CXCL2-CXCR2 axis. The data were produced utilizing publicly-available human specimens (**h**, **f**, **i**) or in-house murine specimens (**a-e**, **g**, **j-o**). For **a-d**, murine spleen cells isolated by Ficoll and LLC tumor cells (LLC) were used; for e and g, NK cells sorted from murine spleen cells were used; for j to i, LLC, sorted NKomega (NKo) / non-NKo, and sorted M1omega (M1o) / non-M1o from murine spleen cells were used. **a**. Proportion of migrating macrophages with anti-CXCR1, anti-CXCR2 and anti-CXCR4 interventions in transwell co-culture system (*n* = 4). Macrophages (BMDM) at the upper chamber of the transwell plate, with tumor cells in the lower chamber. “OE” meaning NLRP4-OE LLC cells in the lower chamber. Blocking antibodies were added to the upper chamber and incubated with macrophages for 6 h before co-culturing with tumor cells. Later, blocking antibodies were washed and after 48 h co-culture with tumor cells, all cells in the lower chamber were collected and analyzed. **b**. Expression of CXCR2 in M0 and M1 from TCGA LUSC and LUAD datasets. **c**. Proportion of migrating macrophages with anti-CXCL2 and CXCL2 supplement in transwell co-culture system (*n* = 4). “OE” meaning NLRP4-OE LLC cells in the lower chamber. The experimental protocol and settings same as in a, while anti-CXCL2/CXCL2 were added and incubated with tumor cells in the lower chamber before co-culture. **d**. Representative ridge plot of macrophages’ migration in transwell co-culture system. “OE” meaning NLRP4-OE LLC cells in the lower chamber. Data was pooled from three independent experiments. **e**. Representative flow cytometric analysis (left) and statistical barplot (right) for migration of NK cells and TIGIT^+^TNFA^+^NK with CCL5 or CXCL10/14 supplement in transwell system (*n* = 4). For this migration assay, CCL5 or CXCL10/14 were added to the lower chamber, with FACS sorted NK cells from mouse spleen at the upper chamber (5*10^5 cells per well). After 48 h, migrated NK cells in the lower chamber were collected and analyzed. **f**. Expression of CCR5 in resting and activated NK cells from TCGA LUSC and LUAD datasets. **g**. Representative ridge plot (left) and statistical barplot (right) for migration of NK cells triggered by tumor cells with CCL5 supplement or silenced by siRNA in transwell co-culture system (*n* = 4). NCOE meaning NLRP4-NCOE LLC cells, and OE meaning NLRP4-OE LLC cells. OE-si-vector meaning scrambled siRNA transduced NLRP4-OE LLC cells, with OE-siCCL5 meaning siCCL5 siRNA transduced NLRP4-OE cells. OE-siCCL5 + CCL5 meaning recombinant murine CCL5 supplement after seeding OE-siCCL5 cells in the lower chamber. Tumor cells were placed in the lower chamber as in a and c, with sorted NK cells at the upper chamber. After 48 h, all cells in the lower chamber were collected and analyzed as described above in a. **h**. Expression of certain genes and ssgsea scores of Macrophages and M1omega ranked by the Z-scores of CXCL2 in TCGA LUAD dataset. The left side in parentheses is the Spearman correlation coefficient, and the right side is the p-value determined by a two-sided nonlinear regression t-test. **i**. Expression of certain genes and ssgsea scores of NK cells and NKomega ranked by the Z-scores of CCL5 in TCGA LUAD dataset. The left side in parentheses is the Spearman correlation coefficient, and the right side is the p-value determined by a two-sided nonlinear regression t-test. **j**. MFI of Annexin V^ +^ LLC induced by sorted NK cells, M1o (M1omega), non-M1o and M2. (*n* = 4) NK cells, M1o, non-M1o and M2 were FACS sorted by: NK cells: CD45^+^NK1.1^+^CD3^−^ cells, M1o: CD11B^+^F4/80^+^CD80^+^ NOS2^+^ cells; non-M1o: CD11B^+^F4/80^+^CD80^+^ TNFA^+^ cells; M2: CD11B^+^F4/80^+^CD80^−^ cells. NK cells sorted directly from mouse spleen cells. Macrophages were polarized from BMDM firstly then sorted. After sorting, they were co-cultured (at the upper chamber, 5*10^5 cells per well) with NLRP4-OE LLC cells (in the lower chamber, 2*10^5 cells per well) for 48 h in transwell plates (membrane pore: 0.4 μm) before apoptosis assay. The apoptosis assay was conducted on LLC cells in the lower chamber (FITC-Annexin V and PE-PI). **k**. Proportion of migrating M1o, non-M1o and M2 with co-existed NK. (*n* = 4) M1o, non-M1o and M2 were sorted and co-cultured with NLRP4-OE LLC cells as in j, but the transwell plate membrane was 8 μm to conduct migration assay. OE meaning that only NLRP4-OE LLC cells in the lower chamber (5*10^5 cells); OE with NK meaning NK cells together with NLRP4-OE cells in the lower chamber (LLC: 2.5*10^5 cells; NK: 2.5*10^5 cells). After 48 h, all cells in the lower chamber were collected and analyzed. **l**. MFI of Annexin V^ +^ LLC induced by sorted NK cells, M1o, non-M1o and M2 with NK or not. (*n* = 4) Experiment settings were the same as in k. The apoptosis was measured in NLRP4-OE LLC cells (gated from PE-CY7 CD45^−^) in the lower chamber. Experiment settings were the same as in k. **m**. Representative flow cytometric analysis (left) and statistical plot (right) for MFI of Annexin V + LLC induced by sorted iNOS^+^M1, TNFA^ +^ M1 and M2 with NK, CXCL2, anti-CXCL2 supplement or not. (*n* = 4) Experiment settings were the same as in l. **n**. Relationship of tumor weight of total protein concentration in TIF. P value was determined by a two-sided nonlinear regression t-test. **o**. The concentration of CXCL2 and CCL5 in TIF. Data represent mean ± SEM; ns *p* > 0.05, *p < 0.05, **p < 0.01, ***p < 0.001,and ****p < 0.0001 from unpaired Student’s t-tests or two-sided nonlinear regression t-tests

Chemokine secretome was extremely complicated by its intertwined many-to-many ligand-receptor interactions under various conditions [72]. Investigating factors responsible for M1omega chemotaxis, we co-cultured murine bone-marrow derived macrophages (BMDM) with tumor cells. Surprisingly, anti-CXCR1/4 rather than anti-CXCR2 significantly diminished the chemoattracted M2 macrophages, while anti-CXCR2 greatly reduced fractions of iNOS^+^ M1 (M1omega) and TNFA^+^ M1, with the former experiencing an even more pronounced reduction (Fig. 4a, S9a). Consistent with transwell assay, our sub-expression analysis in TCGA-LUNG revealed that CXCR2 was the only up-regulated receptor on M1 compared to M0 in a TIME-specific manner (Fig. 4b, S9d). Furthermore, anti-CXCL2 significantly blocked iNOS^+^/TNFA^+^ M1 migration, while showing weaker effects upon M2 (Fig. 4c). CXCL2 supplement successfully rescued blockade effects of anti-CXCL2, indicating that CXCL2-CXCR2, rather than CXCL2-CXCR1/4, specifically regulated iNOS^+^/TNFA^+^ M1 attraction to NLRP4-OE (Fig. 4d).

We initially ensured that CXCL2 had no impact upon NK (Fig. S9b, f), followed by the comparison of CXCL10/14 and CCL5 using transwell assays. Remarkably, CCL5 exhibited better chemotaxis ability for NK compared to CXCL10, whereas CXCL14 showed minimal effects (Fig. 4e). While CXCL10 did reveal certain extent of chemotaxis, it was CCL5 that specifically mediated recruitment of NKomega (TIGIT^+^TNFA^+^ NK) (Fig. 4f). Knockdown of CCL5 and subsequent rescue through CCL5 supplement further emphasized its indispensable role in NLRP4-OE mediated chemotaxis of NK (Fig. 4g). Additionally, direct complement of CCL5 to NC reprogrammed it into mediator for NK attraction (Fig. 4g). Indeed, M1omega and NKomega signatures (Table S5) demonstrated improved correlation power with CXCL2 and CCL5 respectively, compared to macrophages and NK (Fig. 4h-i). Notably, CCL5 exhibited co-expression pattern with M1 markers (CD80, CD86) as well (Fig. 4i).

Subsequently, we collected TIF (Method) and confirmed a strong lineage correlation between tumor weight and protein concentration in corresponding TIF (R^2^ = 0.95) (Fig. 4n). We thus normalized CCL5, CXCL10 and CXCL2 concentration in TIF, and found out that CCL5 along with CXCL2 manifested consistent up-regulation in NLRP4-OE than NC (Fig. 4o). In contrast, CXCL10 appeared susceptible to various sources including CD8^+^ T cells, NK and M (Fig. S9c). In conclusion, we further verified indispensable roles of CCL5 and CXCL2 in-vivo.

### Enhanced anti-tumor capacity induced by collaboration between NKomega and M1omega

As aforementioned, CCL5-CCR5 axis specifically regulated NKomega migration. In contrast, both M1omega and non-M1omega could be significantly recruited by CXCL2-CXCR2 axis. We first utilized the previously-described single-cell sequencing data to validate the exclusive chemokine axes of CCL5-CCR5 and CXCL2-CXCR2 responsible for NKomega and M1omega.

We first wondered whether there could be any difference between their cytotoxicity against tumor cells. However, although tumor apoptosis induced by them outperformed M2, there appeared no difference between them (Fig. 4j). To explore functional characteristics of M1omega and NKomega, we FACS-sorted them out from murine tumor tissues and co-cultured them respectively with tumor cells (Fig. S10a). Surprisingly, M1omega, rather than non-M1omega or M2, experienced significant enhanced migration with co-existed NKomega (Fig. 4k). Consistently, M1omega + NKomega outperformed in the ability to trigger apoptosis of tumor cells (Fig. 4l, Fig. S10b-c). Interestingly, such collaboration could be abolished by anti-CXCL2 and rescued by CXCL2 supplement afterwards (Fig. 4m). We further examined their cytokines secretion during the co-culture. NKomega and M1omega not only exhibited greater production of IFNγ, TNFα and INOS respectively, but also manifested enhanced longevity when added together, suggesting a potential collaborative interaction between NKomega and M1omega (Fig. S10d-f).

On the other hand, chemokine signaling within the TIME is intricate, involving multiple feedback loops and competing signals. To comprehensively profile the chemokine pathways in vivo, we employed the CellChat algorithm to analyze the scRNA-seq data in detail (Fig. S11a). Compared to the NC, the Ccl5-Ccr5 and Cxcl2-Cxcr2 ligand-receptor pairs (L-R pairs) were significantly upregulated in the NLRP4-OE TIME (Fig. S11a), while other chemokine L-R pairs remained unchanged. Notably, tumor cells were the exclusive source of overexpressed Ccl5 and Cxcl2, while Ccr5 + NK cells and Cxcr2 + macrophages were enriched in the NLRP4-OE TIME, a pattern that persisted throughout tumor progression (Fig. S11b-c). This was further validated by Luminex assays on TIF (Fig. S11d). To assess the functional relevance of these findings, we tested whether knockdown of CCL5 and CXCL2 in tumor cells (OE_shCCL5_shCXCL2) could abrogate the anti-tumor effects of NLRP4-OE. Indeed, OE_shCCL5_shCXCL2 significantly reduced NKomega and M1omega infiltration into the TIME, accelerating tumor growth (Fig. S12).

Taken together, we not only uncovered NLRP4-OE mediated CXCL2-CXCR2 and CCL5-CCR5 axes responsible for NLRP4-eco organization, but also depicted the specific cooperation mode between NKomega and M1omega.

### NLRP4-PP2A-PI3K/Akt-NF-kB axis regulation of CCL5 and CXCL2

**Fig. 5:**
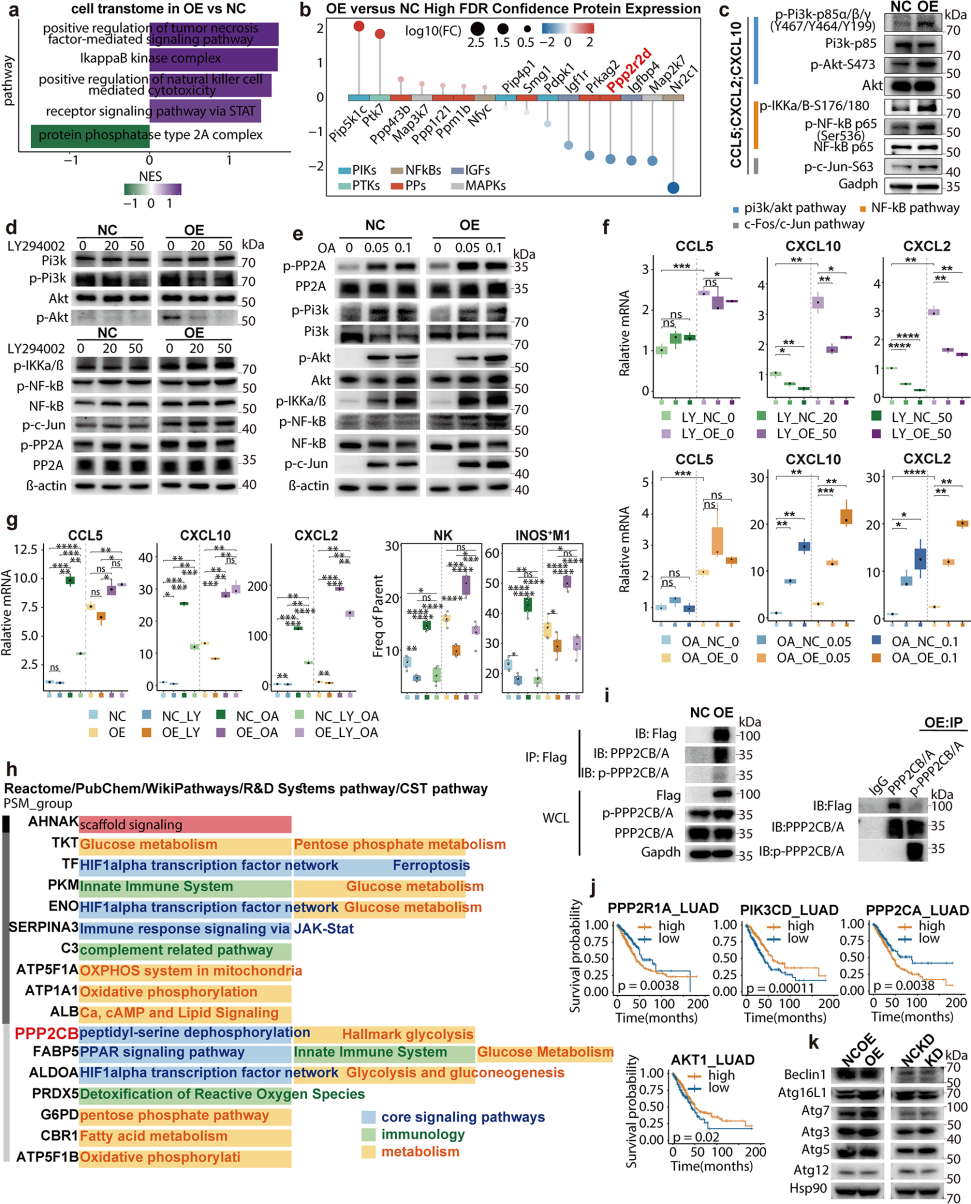
NLRP4-PP2A interaction promotes PI3K/Akt-NF-κB phosphorylation to induce the secretion of CXCL2/CCL5. The data were produced utilizing publicly-available human specimens (**j**) or in-house murine specimens (**a-i**, **k**). For a-i and k, LLC cells were used. **a**. GSEA results of cell transcriptome. **b**. High FDR confidence proteins in NLRP4-OE versus NLRP4-NC from label-free proteomics. **c**. The protein expression levels of p-PI3K, p-AKT, p-IKKαβ, p-NF-kB and p-c-Jun of NLRP4-NC and NLRP4-OE cell lines were measured by Western blot analysis. Data was pooled from three independent experiments. **d**. The protein expression levels in the content of LY294002 were measured by Western blot analysis. (*n* = 3) **e**. The protein expression levels in okadaic acid (OA) content were measured by Western blot analysis. (*n* = 3) **f**. Expression of CXCL2/10 and CCL5 of NLRP4-NC and NLRP4-OE cell lines with PI3K inhibitor (LY) or PP2A inhibitor (OA) or both accessed by qPCR. Data was pooled from three independent experiments. **g**. Expression of CXCL2/10 and CCL5 of NLRP4-NC and NLRP4-OE cell lines with PI3K inhibitor (LY) and PP2A inhibitor (OA) accessed by qPCR (left) and quantification of transitional M1 and NK. Data was pooled from three independent experiments. **h**. Proteins and annotations detected from LS/MS. **i**. Western blot analysis after immunoprecipitation of flag (left), PP2A and p-PP2A (right) in NLRP4-NC and NLRP4-OE cell lines. **j**. Survival curve showing that the expressions of PPP2R1A, PIK3CD, PPP2CA and AKT1 were associated with better OS in TCGA LUAD cohort (HR (95%CI): PPP2R1A: 1.65 (1.21, 1.34); PIK3CD: 0.56 (0.42, 0.75); PPP2CA: 1.58 (1.19, 2.14); AKT1: 0.71 (0.51, 0.97)). **k**. The protein expression levels from autophagy pathway in NLRP4-NC and NLRP4-OE cell lines were measured by Western blot analysis. Data represent mean ± SEM; ns *p* > 0.05, *p < 0.05, **p < 0.01, ***p < 0.001,and ****p < 0.0001 from unpaired Student’s t-tests. The p-value of Kaplan-Meier curves was determined by log-rank test and those of gene enrichment analysis were calculated by R package GSEA

Deconvolution analysis of TIME bulk-RNA seq was based upon its nature of chimeric cell composition, which conversely disturbed the anatomy of tumor-intrinsic features. We thus conducted RNA-seq and label-free proteomics upon NLRP4-OE cell-line. Consistently, positive regulation of TNF and NK-mediated cytoxicity pathways were enriched in RNA-seq (Fig. 5a). Besides, ikappaB kinase-related pathway (NF-kB pathway), which served as the pivotal immunomodulatory axis inducing both CCL5 and CXCL2 expression [73–76], was enriched in RNA-seq either. Astonishingly, at the crossroads there appeared PP2A pathway, which was negatively correlated with NLRP4-OE in RNA-seq and proteomics data (PPP2R2D) simultaneously (Fig. 5b). Moreover, Hoesel et al. highlighted Akt, triggered by PI3K (PI3K/Akt pathway) and directly dephosphorylated by PP2A holoenzyme [77], was one of the major activator of NF-kB in cancer biology [78]. We thus proposed the hypothesis that NLRP4 mediates chemokine reprogramming through PP2A-PI3K/Akt-NF-kB pathway.

As expected, NLRP4 enhanced phosphorylation of PI3K-p85α/β/γ and S473 of Akt, without affecting amounts of PI3K-p85 or Akt, which was the same case as for S536 of NF-kB-p65 and S176/180 of IKKα/β (Fig. 5c). Afterwards, we adopted LY294002, a PI3K inhibitor, to examine dependency of NLRP4 upon PI3K/Akt. LY294002 significantly inhibited activation of PI3K/Akt pathway, represented by diminished p-Akt and p-PI3K (Fig. 5d). CXCL2 along with CXCL10 could also be substantially suppressed even with NLRP4-OE (Fig. 5f). CCL5 demonstrated relatively moderate but significant decline with larger amount of LY294002. Considering alteration of NF-kB pathway was minimal under LY294002 intervention (Fig. 5d), we therefore hypothesized that an up-stream mediator, such as PP2A, assumed more responsibility here. Phosphorylation of PP2A (Y307) was its major post-translational modification indicating decreased activity [79], while dephosphorylation of Akt by PP2A suppressed PI3K/Akt pathway. Indeed, NLRP4-OE revealed pronounced p-PP2A up-regulation. Meanwhile, okadaic acid (OA), a PP2A core enzyme inhibitor, remarkably dampened PP2A activity manifested by up-regulated p-PP2A in a concentration-dependent manner, with synchronous liberated expression of p-PI3K, p-Akt and down-stream p-IKKα/β along with p-NF-kB, consistently within NC and NLRP4-OE (Fig. 5e). Noticeably, CCL5 manifested predominant enhancement with OA supplement, in accordance with CXCL2 and CXCL10 (Fig. 5f). Furthermore, LY294002 had no effect upon p-PP2A, suggesting a dependency of PI3K/Akt upon PP2A rather than vise-versa. Indeed, rescue of OA intervention with LY294002 afterwards significantly reversed its stimulation of CCL5 and CXCL2/10 to certain extent, accompanied by consistent tendency in chemotaxis of iNOS^+^ M1 and NK (Fig. 5g, Fig. S13a-b). Therefore, we concluded that NLRP4-OE amplified CXCL2, CCL5 and CXCL10 through PP2A-PI3K/Akt-NF-kB axis by dampening PP2A phosphatase activity.

To facilitate understanding of the exact modulation of NLRP4 upon PP2A, we further utilized co-IP and LC-MS/MS to determine protein-protein interactions of NLRP4. AHNAK, a scaffold protein responsible for possible regulation of PI3K/Akt [80] as well as interruption of PP2A activity [81], was identified, with verification of its moderate strengthened phosphorylation by IHC in NLRP4-OE (Fig. S13c). More importantly, PPP2CB, a beta isoform of the catalytic subunit indispensable for PP2A core-enzyme, was significantly enriched by LC-MS/MS either (Fig. 5h). We set out to perform co-IP of NLRP4 with PPP2CA/B or p-PP2A. It appeared that NLRP4 possessed direct interaction with PPP2CA/B, but dissociated with p-PP2A, leading to accumulation of such disactivated form of PP2A (Fig. 5i). In alignment of our hypothesis, diminished expression of PPP2CA and PPP2R1A, which together assembled the PP2A core enzyme, correlated with better OS in TCGA-LUAD (Fig. 5j). Besides, knockdown of PP2A in tumor cells stimulated the PI3K/Akt pathway as well (Fig. S14a-b). Meanwhile, PIK3CD, AKT1 and IKBKB all demonstrated positive correlation with OS (Fig. S13d-e). In addition, NLRP4 could negatively regulate autophagic process through association with BECN1 [26]. Herein in the context of NLRP4-OE, there appeared no evidence no matter in BECN1 expression or in alteration of major autophagy-related (ATG) protein family members (Fig. 5k).

We next tested whether in vivo inhibition of PP2A could recapitulate the anti-tumor effects of NLRP4. Given PP2A’s broad regulatory role across various cell types, we first examined whether PP2A knockout in mice would impact NLRP4’s anti-tumor activity. Tumor suppression remained effective in PP2A^−/−^ mice (Fig. S12a-c), likely due to beneficial effects of PP2A downregulation in immune cells like T cells and NK cells [82–84]. Additionally, PP2A knockdown in tumor cells (PP2A-KD) resulted in significant tumor shrinkage and increased NKomega and M1omega infiltration (Fig. S14d-e). The PP2A inhibitor LB-100 similarly mimicked these anti-tumor effects (Fig. S14f-h). scRNA-seq confirmed that LB-100 boosted expression of NKomega and M1omega signature genes, and LB-100 treatment also enhanced antigen presentation by DCs (Fig. S15a-e), supporting our mFC findings. In conclusion, PP2A-KD or LB-100 effectively recapitulate NLRP4’s anti-tumor effects, suggesting potential for clinical translation of NLRP4-eco.

### NAMPT intertwined in crosstalk of NKomega and M1omega

As aforementioned, through in-vivo, ex-vivo and in-silico experiments, we have established the definitive presence of iNOS^+^ M1 (M1omega) and TIGIT^+^TNFA^+^ NK (NKomega) as integral constituents of NLRP4-eco. However, phenotypic distinctions of M1omega/NKomega in comparison to other M/NK subtypes in human and murine TIME remained elusive.

**Fig. 6:**
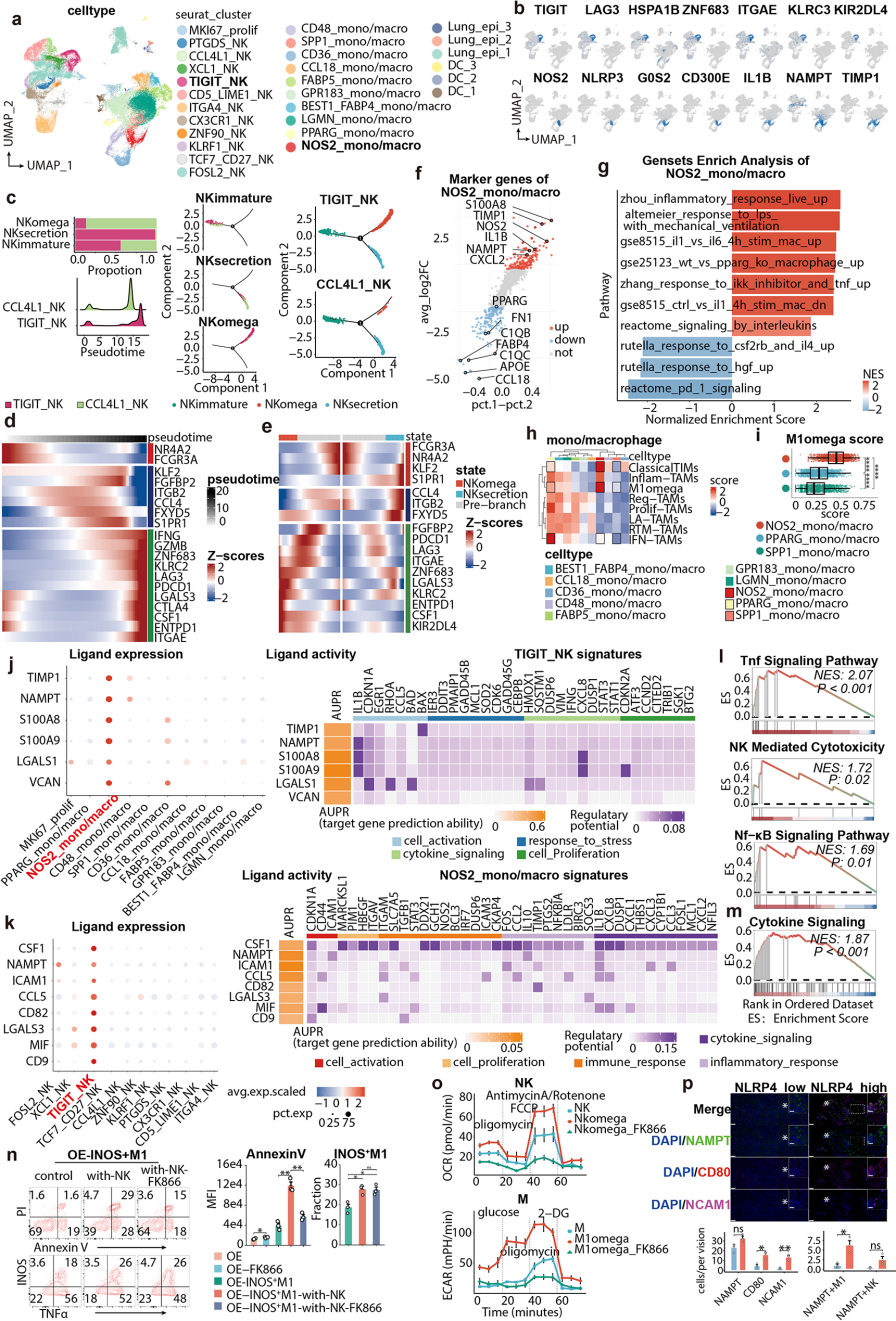
eNAMPT is identified as a NKomega-M1omega axis mediator in human NSCLC through the single-cell data analysis The data were produced utilizing publicly-available (**a-m**) or in-house human specimens (**p**) or in-house murine specimens (**n**, **o**). For **o**, sorted iNOS^+^M1 cells from murine spleen cells were used. **a**. UMAP plot of 50,215 cells colored by clusters in an integrated cohort. **b**. UMAP feature plots showing the expression levels of certain genes. **c**. Distribution of CCL4L1_NK and TIGIT_NK among cell states (upper and right) and along pseudotime (lower). **d**. Heat map of gene expression along pseudotime. **e**. Heatmap of gene expression in different cell states. **f**. Volcano plot showing marker genes of NOS2_mono/macro. **g**. GSEA results from NOS2_mono/macro. **h**. Heat map of literature-derived signatures scores in macrophages. **i**. M1omega score in NOS2_mono/macro, PPARG_mono/macro and SPP1_mono/macro. **j-k**. NicheNet analysis shows the potential ligands expressed by neighboring cells that presumably affected the marker genes of TIGIT_NK or NOS2_mono/macro (left). Ligand activity indicates the ability of each ligand to predict the target genes, and better predictive ligands are thus ranked higher (middle). The regulatory potential score indicates the confidence that a particular ligand can regulate the expression of a particular target gene (right). **l-m**. GSEA plot from potential target genes of TIGIT_NK (l) and NOS2_mono/macro (m). **n**. Representative flow cytometric analysis (left) and statistical plot (middle) for MFI of Annexin V + LLC induced by sorted iNOS^+^M1 with antecedent NK existed or NAMPT inhibitor (FK866) supplement. (*n* = 4) **o**. Bioenergetic profiles of NK, NKomega, M and M1omega with NAMPT inhibitor (FK866) are shown. The Seahorse XF cell Mito stress test was used to define the bioenergetic profiles. The changes in ECAR and OCR for the time indicated during an XF24 extracellular flux analyzer run are plotted. The dash lines indicate the injection time points. FCCP, carbonyl cyanide-4 (trifluoromethoxy) phenylhydrazone. **p**. Representative example of NLRP4-low and NLRP4-high patients. Tumor staining by multiplexed IHC shows the spatial distributions of NAMPT + M1, NAMPT + NK and eNAMPT in tumor interstitial fluid. Images are representative of at least three images for individual mice. DAPI, 4,6-diamidino-2-phenylindole. Data represent mean ± SEM; ns *p* > 0.05, *p < 0.05, **p < 0.01, ***p < 0.001,and ****p < 0.0001 from unpaired Student’s t-tests. The p-values of gene enrichment analysis were calculated by R package GSEA

We first collected, curated and integrated a human NSCLC NK/M cells atlas (Fig. 6a-b) and successfully identified M1omega/NKomega as well (Fig. 6c-i, Fig. S17a). We then proceeded to dissect cell-cell interactions between them. Surprisingly, top target genes in TIGIT^+^ NK (referred to as NKomega-tar) were mediated by ligands that up-regulated in NOS2^+^ M1 and vice versa (M1omega-tar) (Fig. 6j-k). Additionally, NKomega-tar were enriched in TNF signaling pathways (Fig. 6l), whereas M1omega-tar were involved in NOS2/IL1B-contained cytokine signaling axis (Fig. 6m). Notably, NAMPT, the rate-limiting enzyme participated in NAD^+^ salvage pathway, emerged as the intersected gene among the ligands corresponding to NKomega-tar and M1omega-tar. NAMPT exists in both extracellular (eNAMPT) and intracellular (iNAMPT) forms, with eNAMPT boosting TLR4-independent macrophages activation [85], and iNAMPT mediating NK mitochondrial homeostasis [86], suggesting anti-tumor cytoxicity (Fig. S19b). We thus postulated that NAMPT might facilitate communication between NKomega and M1omega.

We further validated our findings in murine tumors using scRNA-seq of tumor-infiltrating NK cells at days 11, 15, and 21, integrating the data to track phenotypic changes (Fig. S20a-c). NK_3 cells showed increased expression of Tigit, Tnfα, Ccr5, and Nampt, resembling NKomega (Fig. S20b-e), supporting the human scRNA-seq results. We also identified NK_5, enriched in NC tumors, with upregulated stress-related genes like HSPA1A/B (Fig. S20b-e), confirming Zhang et al.‘s finding that stressed NK cells, rather than those with exhaustion markers like TIGIT, are linked to immunotherapy resistance [87]. Additionally, we also detected M1omega-like macrophages (M_4) in the TME of NLRP4-OE tumors, uniquely expressing Nampt and Cxcr2 (Fig. S20g-j), while M_2 macrophages, expressing senescence-related genes like Cdkn2a and Cxcr1, were enriched in NC tumors (Fig. S20j-k). Senescent macrophages have been linked to lung cancer progression [88]. In summary, NLRP4-OE selectively induces infiltration of M1omega and NKomega cells, both specifically expressing NAMPT.

We comprehensively assessed iNAMPT and eNAMPT expression in murine primary cells (Fig. 6p). NLRP4-OE didn’t affect iNAMPT/eNAMPT levels in tumor cells, and neither macrophages nor NK could (Fig. S19). Astonishingly, NLRP4-OE significantly increased iNAMPT in both NK and macrophages (Fig. S19). Meanwhile, eNAMPT experienced substantial up-regulation during co-culture. Besides, FK866, a selective NAMPT inhibitor, largely inhibited NLRP4-induced tumor cells apoptosis (Fig. 6n). Mechanistically, NAMPT reprogrammed NK to oxidative phosphorylation (OXPHOS)-addicted phenotype, and elevated glycolysis rates in macrophages as well (Fig. 6o), which could be abolished by FK866, in accordance with previous literature [86, 89].

NAMPT is known to contribute to metabolic reprogramming in various cell types beyond macrophages and NK cells, leaving its therapeutic potential uncertain [86, 90–93]. In this study, we found that while intra-tumor injection of eNAMPT did not suppress tumor growth (Fig. S21a-b)—possibly relevant to upregulated PD-L1 on tumor cells (Fig. S21c-d)—adoptive transfer of NKomega, M1omega, or both significantly enhanced anti-tumor immunity in vivo (Fig. S21e-f). However, knockout of NAMPT in NKomega or M1omega (NAM^−/−^NKo, NAM^−/−^M1o) largely abolished such effects (Fig. S21e-f). NAM^−/−^NKo exhibited a stressed phenotype, while NAM^−/−^M1o showed a senescent phenotype (Fig. S21g). Supplementation with eNAMPT rescued these phenotypic changes, supporting the scRNA-seq findings. We hypothesized that NAMPT transfer between NKomega and M1omega might enhance iNAMPT expression and cytotoxic cytokine secretion. Indeed, co-culture of NAM^−/−^NKo with M1omega increased iNAMPT expression and TNFα secretion compared to NAM^−/−^NKo alone, which is the same case for M1omega (Fig. S21h).

Taken together, we proved that eNAMPT, but not iNAMPT, was directly correlated with enhanced cytotoxicity observed in NLRP4-eco as aforementioned. Unveiled by Nichenet analysis in single-cell datasets, NAMPT was found to play a dual role. It not only underpinned anti-tumor capabilities of NKomega and M1omega in NLRP4-eco (Fig. 6p), but also served as the communication bridge between them, which was independent of the well-established chemokines reprogramming effects.

## Discussion

Genomics-driven cancer biology has ushered in a new era of targeted precision regimens, exemplified by AZD9291 in EGFR-mut NSCLC [94]. The second revolution was sparked by ICIs to set exhausted T cells back on track, which normalized adaptive immunity for effective immune recognition [4]. However, analogous to the emergence of numerous subclone branches during TKIs resistance [95], the complex evolution within TIME brought by ICIs resistance prompted us to adopt a cell-state-centric ecosystem model [17]. Considering the intertwined “lock” of various cell states within TIME, identification of the specific “key” assumed paramount importance. In this study, we unveiled a novel role for NLRP4 within NSCLC TIME, acting as the catalyst to unlock an anti-tumor NLRP4-eco composed of NKomega and M1omega, much like identifying the driver mutation that underpins a particular cancer molecular subtype.

In a broader context, similar to the scenario involving NLRP4, tumor-intrinsic factors can exert diverse impacts on extrinsic TIME. Lou et al. have elucidated that MET amplification in NSCLC induced STING downregulation through UPF1 phosphorylation [96]. Additionally, Heymach et al. demonstrated that LKB1-deficient LUAD mediates resistance to ICIs via MCT4-driven lactate secretion [97]. Herein we have unveiled a hitherto obscure role of NLRP4, which orchestrated direct interaction with PPP2CA/B, subsequently triggering chemokine reprogramming through PI3K-Akt-NF-kB axis and thus curtailing tumor growth in a T-cells-independent manner. Intriguingly, recent work by Merad et al. has revealed that TREM2^+^ macrophages attenuated NK activity via IL18/IL18BP L-R [98]. Furthermore, the reactivation of NK cells combined with TREM2 blockade successfully reversed lung tumor growth, devoid of any T cell participation as well [98]. To sum up, the emergence of this distinctive M-NK ecosystem holds immense translational potential, providing an alternative avenue in cases where T cells-centered ICIs-com strategies falter.

Compared to T cells, NK remained largely unexplored, especially for their differentiation trajectory in TIME. We here identified NKomega residing in NLRP4-eco as TIGIT^+^TNFA^+^ NK. NKomega demonstrated exhaustion and cytoxicity simultaneously, analogous to the multifaceted role of PD-1^+^ TIL. PD-1^mid^CXCR5^+^ CD8^+^ T cells, rather than being dysfunctional, acted as responders to ICIs [99, 100]. Conversely, PD-1high “burned-out” CD8^+^ T cells exhibited resistance to ICIs [101]. Intriguingly, Copik et al. recently also proved that TIGIT was marker for NK activation [102], aligned with the observed behavior of NKomega. Moreover, anti-TIGIT treatment indeed enhanced NK cell even further, as evident in our co-culture experiments, Copik et al.‘s spheroid system [102], and murine models. This paradoxical behavior of TIGIT, reminiscent of PD-1, was ascribed by Copik et al. to prolonged exposure to tumor cells, leading to eventual anergy of NK. Additionally, we posited that continuous chemotaxis facilitated by NLRP4-OE secreted CCL5 along with the synergistic support of iNOS^+^ M1 through NAMPT communication were pivotal for NKomega to preserve their cytotoxicity. Meanwhile, anti-TIGIT therapy served as a “last-ditch” intervention, akin to the role of neoadjuvant ICIs in boosting CXCL13^+^ Tex [103]. However, the precise mechanisms governing TNFA expression via TIGIT signaling, as well as its relationship with NAMPT-mediated NAD^+^ metabolism, still warrant further investigation.

As aforementioned, except for NKomega, iNOS^+^ M1 was another indispensable component of NLRP4-eco. Lal et al. demonstrated that iNOS^+^CD206^−^ M were essential for α-GalCer-mediated NKT cells infiltration [104], corroborating our findings. However, it was noteworthy that T cells have not been excluded by them, and the specific mechanisms governing iNOS^+^ M1 infiltration as well as its interaction with NK instead of NKT cells remained elusive. Innovatively, we confirmed that NLRP4-OE exploited CXCL2-CXCR2 to selectively recruit M1omega, a process that facilitated NKomega cytoxicity, surpassing that induced by TNFA^+^ M1. CXCL2/CXCR2 axis in tumor-infiltrating myeloid-cells was highly context-dependent. On one hand, when secreted by glioblastoma stem cells, CXCL8-CXCR2 polarized M towards M2 [105]. On the other hand, TIL-secreted IL-22 prompted CRC to recruit anti-tumor TAN through CXCL2 [106]. Meanwhile, although CXCL2-CXCR2 signaling has been well-recognized in pro-inflammatory M infiltration in hypertensive retinopathy [107], sciatic nerve [108] and steatohepatitis [109], the selective attraction to M1omega here was an inaugural identification. To be mentioned, we conducted comprehensive experiments to exclude any interference from CXCL1/3 and CXCR1/4, which were often overlooked previously.

The last piece of the puzzle is the communication between NKomega and M1omega. Through single-cell analysis, we revealed eNAMPT as the potential transducer between NKomega and M1omega, a mechanism remained hitherto unexplored. Although we have demonstrated that NAMPT is essential for the cytotoxicity of NKomega and M1omega, the specific mechanisms by which eNAMPT mediates the interaction between these two cell types remain unclear. We plan to further investigate this in our future work.

## Conclusion

This study reveals a role of NLRP4 in shaping a distinct anti-tumor microenvironment, driven by NKomega (TIGIT^+^TNFA^+^ NK cells) and M1omega (iNOS^+^ M1 macrophages). Mechanistically, NLRP4 promoted CCL5 and CXCL2 secretion through its negative control of PP2A. We also observed a potential differentiation trajectory of NKomega into stressed NK cells and M1omega into senescent macrophages, processes that may be modulated by eNAMPT supplementation. In conclusion, we successfully unraveled a previously undisclosed role of NLRP4, which unlocked a TIME ecosystem composed of NKomega and M1omega, highlighting alternative therapeutic avenues in parallel with the tumor-antigen-dependent adaptive immunotherapies.

## Electronic supplementary material

Below is the link to the electronic supplementary material.

Supplementary Material 1

Supplementary Material 2

## Data Availability

No datasets were generated or analysed during the current study.

## Abbreviations

NSCLC: Non-Small Cell Lung Carcinoma
anti-PD1/L1: Programmed cell death 1/ligand 1
TIGIT: T Cell Immunoreceptor With Ig And ITIM Domains
CXCL9: C-X-C Motif Chemokine Ligand 9
CCL5: C-C Motif Chemokine Ligand 5
NAMPT: Nicotinamide Phosphoribosyl transferase
PPP2CB: Protein Phosphatase 2 Catalytic Subunit Beta
sequential-HYGTIC: Sequential tumor-immune co-culture 3D system
αPD-L1-eco: Tumor ecosystem triggered by αPD-L1
NLRP4-eco: Tumor ecosystem triggered by NLRP4
