# Supplementary material for: NLRP4 unlocks an NK/macrophages-centered ecosystem to suppress non-small cell lung cancer
Source: Biomark Res. 2025 Mar 14;13:44. doi: 10.1186/s40364-025-00756-4 (PMC11909883; doi:10.1186/s40364-025-00756-4)
Supplement: Supplementary file 1 — Supplementary Material 1 [file 40364_2025_756_MOESM1_ESM.docx]

Supplementary Materials for

**NLRP4 unlocks an NK/macrophages-centered ecosystem to suppress non-small cell lung cancer**

Zhouwenli Meng#, Jian Li#, Hui Wang#, Zhengqi Cao, Wenqing Lu, Xiaomin Niu, Yi Yang, Ziming Li*, Ying Wang*, Shun Lu* (#Co-first authors, *Co-corresponding authors)

Correspondence to: ywangssmu@shsmu.edu.cn

liziming1980@shsmu.edu.cn

[shunlu@sjtu.edu.cn](mailto:shunlu@sjtu.edu.cn)

**This PDF file includes:**

Figures. S1 to S21

**Figure. S1.**

**
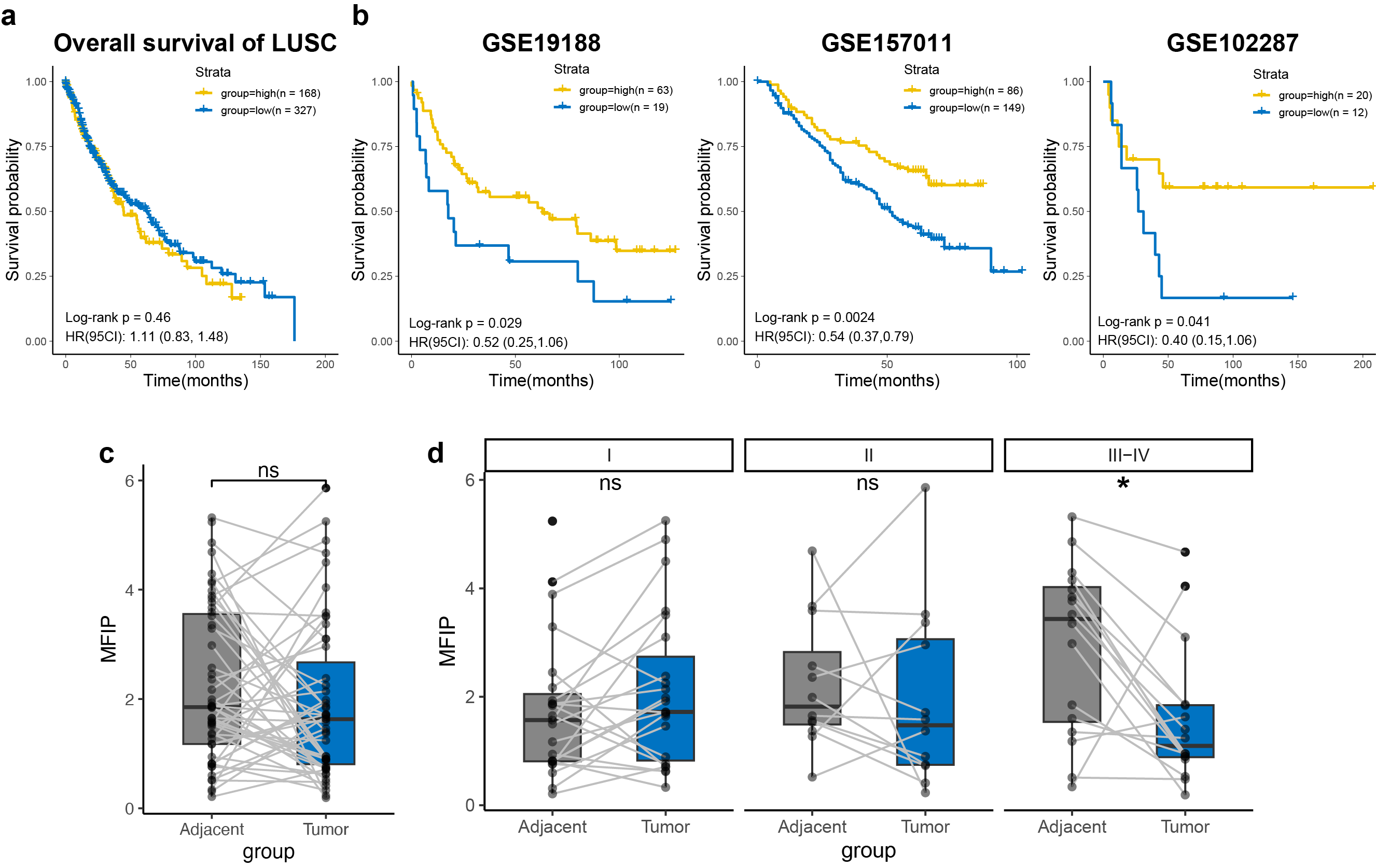
**

**FigS1. NLRP4 expression predicts improved survival in NSCLC while revealing stage-specific variations.**

The data were produced utilizing publicly-available (a, b) or in-house human specimens (c, d).

a. Kaplan-Meier curves analysis showing that NLRP4 associated with better OS in TCGA_LUSC. Two-sided log-rank test.

b. Kaplan-Meier curves analysis showing NLRP4 associated with better OS in GSE19188 (n = 82), GSE157011 (n=235), GSE102287 (n =32). Two-sided log-rank test.

c. MFIP of NLRP4 in tumors with paired adjacent tissue. Paired Student’s t-tests.

d. MFIP of NLRP4 in tumors with paired adjacent tissue in different clinical stages. Paired Student’s t-tests.

ns p > 0.05, *p < 0.05, **p < 0.01, ***p < 0.001 ,and ****p < 0.0001 from unpaired Student’s t-test. The p-value of Kaplan-Meier curves was determined by log-rank test.

**Figure. S2.**


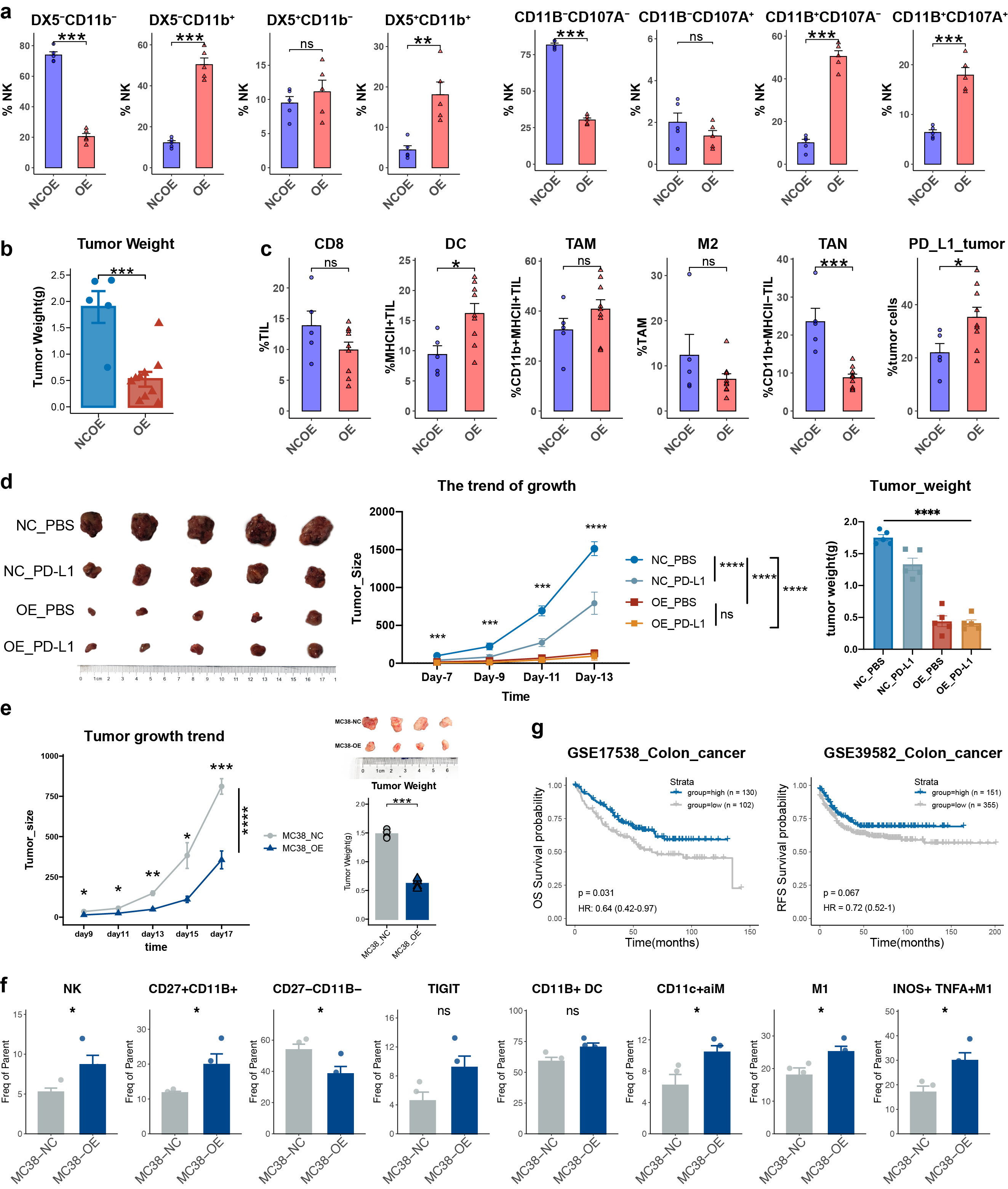


**FigS2. NLRP4-eco outperforms αPD-L1-eco exclusively through interactions with defined non-T cell populations.**

The data were produced utilizing publicly-available human specimens (g) or in-house murine specimens (a-e, f).

a. Proportion of intertumoral subpopulations of NK. Data pooled from three independent experiments.

b. Tumor weights of mice bearing NLRP4-NCOE and NLRP4-OE LLC. (n = 5-8 mice per group, representative of three independent experiments).

c. Proportion of intertumoral immune populations. Data pooled from three independent experiments.

d. Growth of LLC NLRP4-NCOE and NLRP4-OE in immunocompetent C57BL/6 mice, treated with anti-PDL1 or PBS. (n = 5-8 mice per group, representative of two independent experiments). Tumors dissected and photographed (left). Tumor growth curves (middle) and tumor weights (right) of mice bearing tumor cells.

e. Growth of MC38 NLRP4-NCOE and NLRP4-OE in immunocompetent C57BL/6 mice. (n = 5-8 mice per group, representative of two independent experiments). Tumors dissected and photographed (right upper). Tumor growth curves (left) and tumor weights (right lower) of mice bearing tumor cells.

f. Proportion of intertumoral immune populations from mice bearing NLRP4-NCOE and NLRP4-OE MC38. Data pooled from three independent experiments.

g. Kaplan-Meier curves analysis showing NLRP4 associated with better OS and RFS in GSE17538 (n = 232) and GSE39582 (n = 506). Two-sided log-rank test.

Data represent mean ± SEM; *p < 0.05, **p < 0.01, and ***p < 0.001 from unpaired Student’s t-tests. Tumor growth assessed using multiple regression analyses.

Data represent mean ± SEM; ns p > 0.05, *p < 0.05, **p < 0.01, ***p < 0.001 ,and ****p < 0.0001 from unpaired Student’s t-test, one-way ANOVA and two-way ANOVA followed by Tukey’s HSD post - hoc test for pairwise comparisons. Tumor growth was assessed using two-way ANOVA test.

**Figure. S3.**


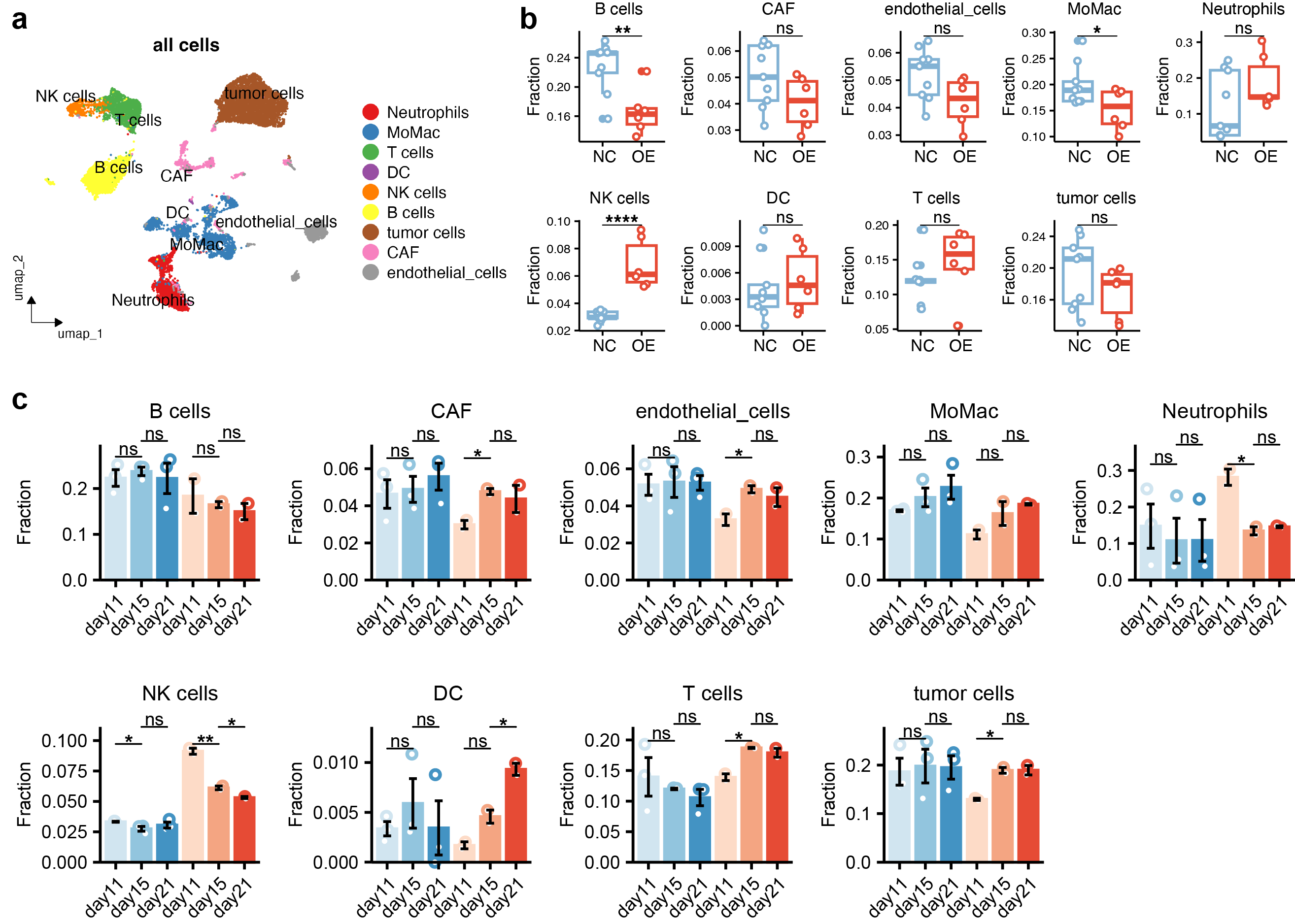


**Fig.S3. NK and M subset exhibit pronounced enrichment within NLRP4-eco at single-cell resolution.**

The data were produced utilizing in-house murine specimens.

a.UMAP plot of all cells colored by clusters from the tumor tissue of mice bearing NLRP4-NC and NLRP4-OE at day11, day15 and day21.

b.Fractions of different clusters in the NLRP4-NC and NLRP4-OE group.

c.Fractions of different clusters in the NLRP4-NC and NLRP4-OE group at different time points.

Data represent mean ± SEM; ns p > 0.05, *p < 0.05, **p < 0.01, ***p < 0.001 ,and ****p < 0.0001 from unpaired Student’s t-test.

**Figure. S4.**

**
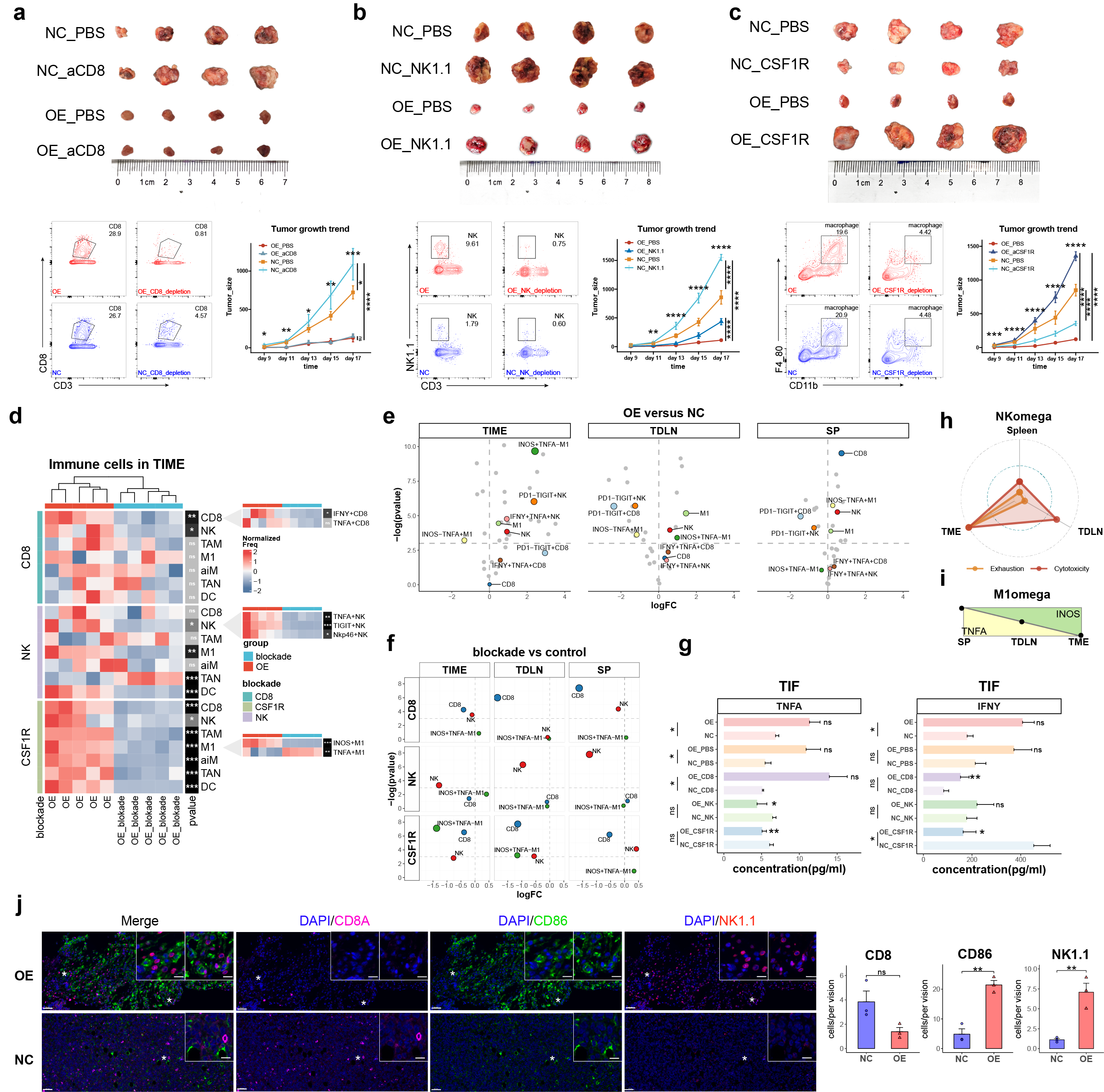
**

**FigS4: NK and M dominate NLRP4-eco independently of CD8+ T cells through spatially heterogeneous mechanisms.**

The data were produced utilizing in-house murine specimens.

a-c. Mice bearing NLRP4-NC and NLRP4-OE LLC cells treated with PBS, anti-CD8, anti-NK1.1 or anti-CSF1R antibodies, with tumors dissected and photographed (upper). Representative flow cytometric analysis of CD8^+^ cells/NK cells/macrophages in tumors of different groups (lower left). Tumor growth curves of mice bearing tumor cells (lower right).

d. Heatmap showing the frequency of intertumoral immune cells (left) and their functional subpopulations (right). (n = 5 mice per group, representative of three independent experiments).

e. Volcano plot showing the different proportion of immune populations in TIME, TDLN and SP from mice bearing NLRP4-NC versus NLRP4-OE LLC. Data pooled from three independent experiments.

f. Volcano plot showing the different proportion of INOS^+^M1, NK and CD8^+^ cells in TIME, TDLN and SP from mice bearing NLRP4-OE LLC treated with PBS/aCD8/NK1.1/aCSF1R. Data pooled from three independent experiments.

g. The concentration of TNFA and IFNY in TIF.

h-i. Pattern diagram for NKomega and M1omega.

j. Representative example of NLRP4-NC and NLRP4-OE tumors. Tumor staining by multiplexed IF showing the spatial distributions of D8^+^ T cells, M1 and NK cells. Images are representative of at least three sections for individual mice. DAPI, 4,6-diamidino-2-phenylindole.

Data represent mean ± SEM; *p < 0.05, **p < 0.01, and ***p < 0.001 from unpaired Student’s t-tests. Tumor growth was assessed using multiple regression analyses.

Data represent mean ± SEM; ns p > 0.05, *p < 0.05, **p < 0.01, ***p < 0.001 ,and ****p < 0.0001 from unpaired Student’s t-test, one-way ANOVA and two-way ANOVA followed by Tukey’s HSD post - hoc test for pairwise comparisons. Tumor growth was assessed using two-way ANOVA test.

**Figure. S5.**


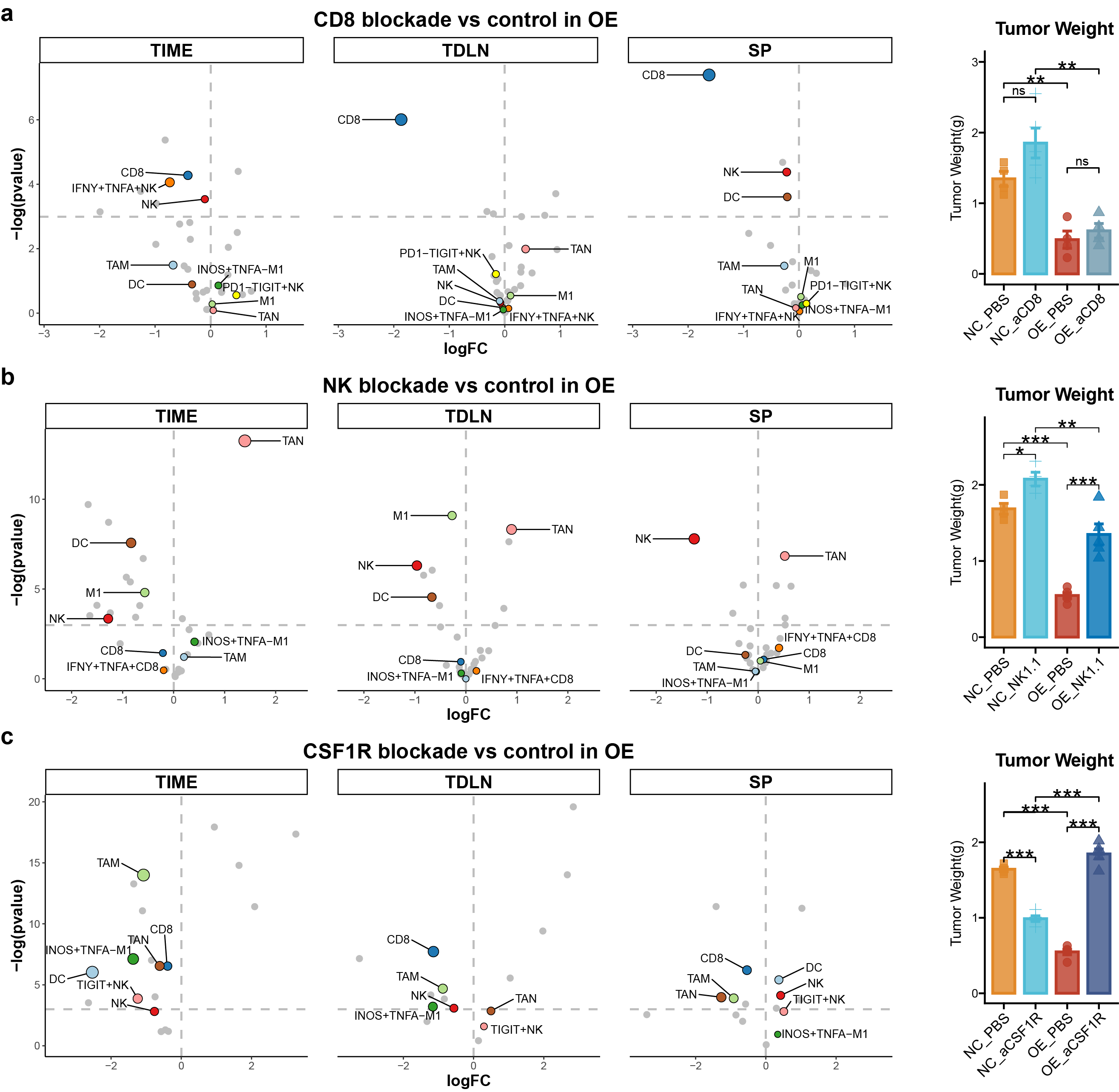


**FigS5. NLRP4-eco exhibits spatially heterogeneous dynamics under depletion of NK, M, and CD8+ T cells.**

The data were produced utilizing in-house murine specimens.

a-c. Different proportions of cell lineage in TIME, TDLN and SP from mice bearing NLRP4-OE LLC treated with PBS/aCD8/NK1.1/aCSF1R (left) and tumor weights from each group (right). Data was pooled from two independent experiments.

Data represent mean ± SEM; ns p > 0.05, *p < 0.05, **p < 0.01, ***p < 0.001 ,and ****p < 0.0001 from unpaired Student’s t-test.

.

**Figure. S6.**


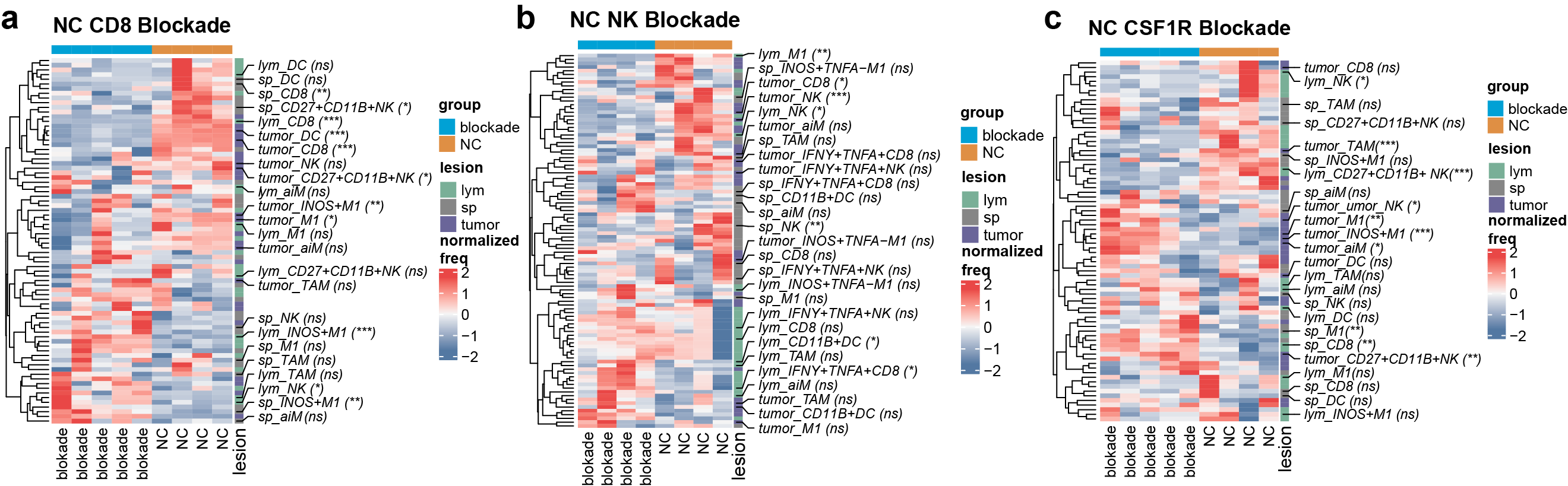


**FigS6. WT-eco exhibits CD8 T cells-dependent features.**

The data were produced utilizing in-house murine specimens.

a-c. Heatmaps showing different proportions of cell lineage in TIME, TDLN and SP from mice bearing NLRP4-NCOE LLC treated with PBS/aCD8/NK1.1/aCSF1R.

Data represent mean ± SEM; ns p > 0.05, *p < 0.05, **p < 0.01, ***p < 0.001 ,and ****p < 0.0001 from unpaired Student’s t-test.

**Figure. S7.**


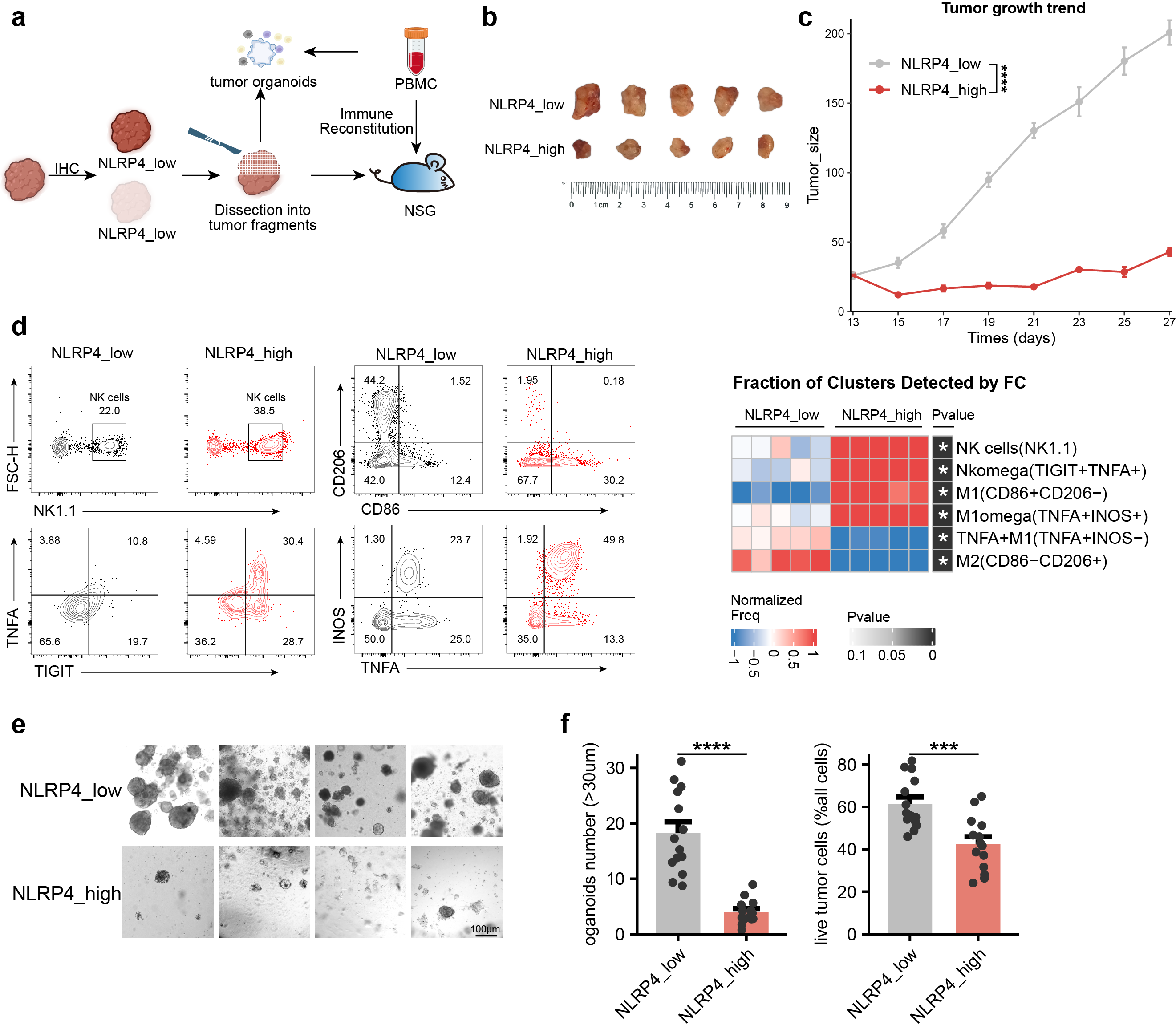


**FigS7. NLRP4 mediates an anti-tumor ecosystem in human tissues similar to that in mice.**

The data were produced utilizing in-house human specimens.

a.Experimental design for validation by human tissues and PBMC in b-f.

b.Tumors dissected from NSG mice bearing NLRP4_low and NLRP4_high human tissue with immune reconstitution by intravenous infusion of immune cells from PBMC.

c.Growth of NLRP4_low and NLRP4_high tissue in NSG mice in b.

d.Representative flow cytometric analysis for NK, M1 and their subsets in the TME of mice in b and (left) and the fractions of them visualized through heatmap (right).

e.Images of organoids cultured from NLRP4_low and NLRP4_high tissues.

f. Statistical plot showing organoid numbers and live tumor cells in NLRP4_low and NLRP4_high organoids.

Data represent mean ± SEM; ns p > 0.05, *p < 0.05, **p < 0.01, ***p < 0.001 ,and ****p < 0.0001 from unpaired Student’s t-test and two-way ANOVA followed by Tukey’s HSD post - hoc test for pairwise comparisons. Tumor growth was assessed using two-way ANOVA test.

**Figure. S8.**


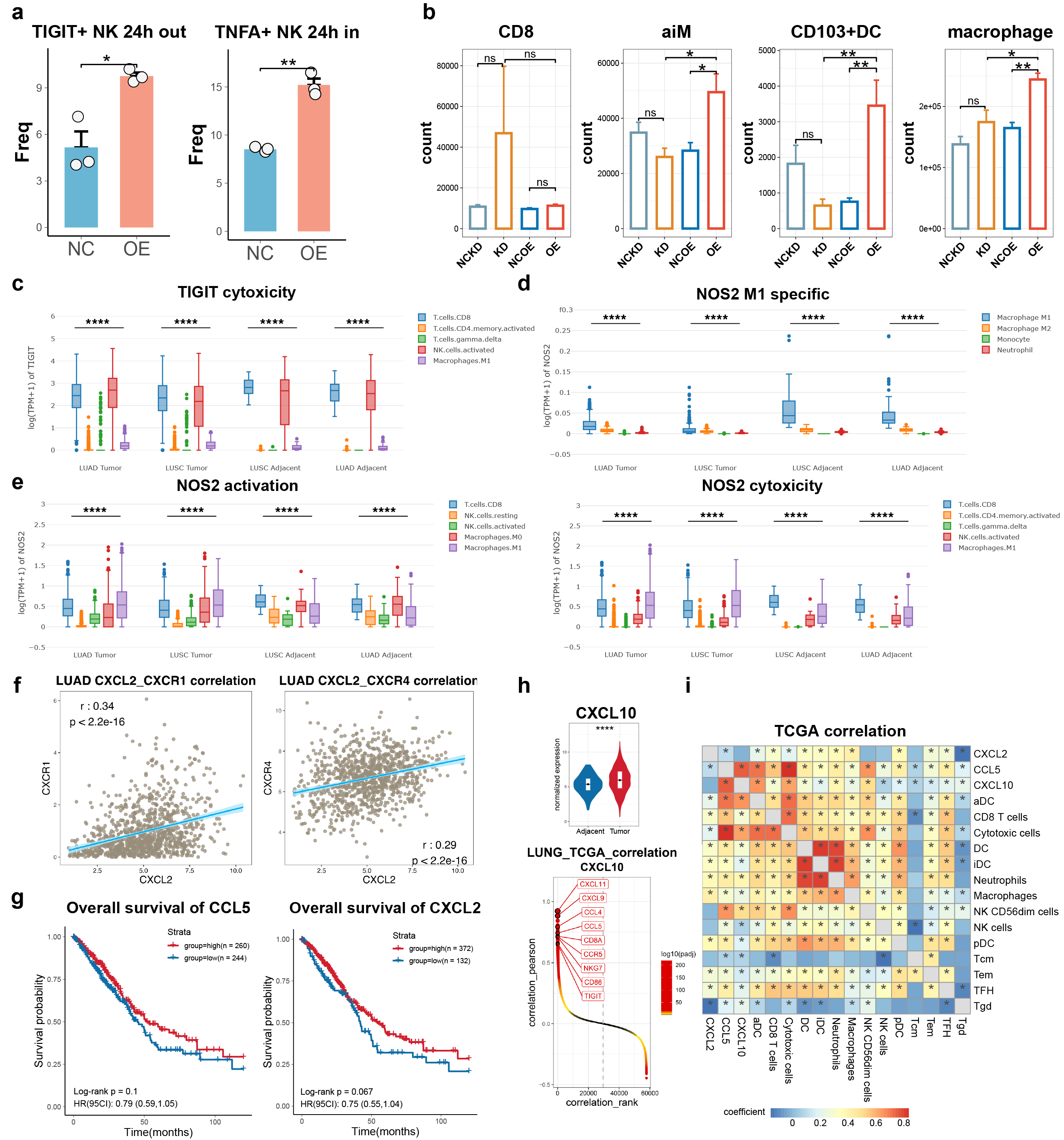


**FigS8. The function of CXCL2, NOS2 and TIGIT are clarified in NSCLC scenario**

The data were produced utilizing publicly-available human specimens (c-i) or in-house murine specimens (a, b).

a. Proportion of TIGIT^+^NK and TNFA^+^NK in tumor-immune co-culture 3D system.

b. Quantification of transitional CD8, aiM, CD103^+^DC and macrophage. (n=4)

c. TIGIT expressions among immune populations in TCGA LUAD datasets.

d. NOS2 expressions in macrophage lineages in TCGA LUAD datasets.

e. NOS2 expressions among immune populations in TCGA LUAD datasets.

f. Relationship of CXCL2 with CXCR1 (left) and CXCL2 with CXCR4 (right) in TCGA datasets (LUAD).

g. Survival curve showing that the expression of CXCL2 and CCL5 was associated with better OS in TCGA LUAD cohort.

h. Expression of CXCL10 in tumor versus adjacent from TCGA LUAD datasets (upper) and genes correlated to CXCL10 in TCGA LUNG (lower). Genes ranked by the Pearson correlation coefficient and colored by adjusted p-value.

i. Heat map showing correlations between CXCL2/10, CCL5 and immune populations in TCGA LUAD cohort.

Data represent mean ± SEM; ns p > 0.05, *p < 0.05, **p < 0.01, ***p < 0.001 ,and ****p < 0.0001 from unpaired Student’s t-tests or one-way ANOVA test. The p-value of Kaplan-Meier curves was determined by log-rank test.

**Figure. S9.**


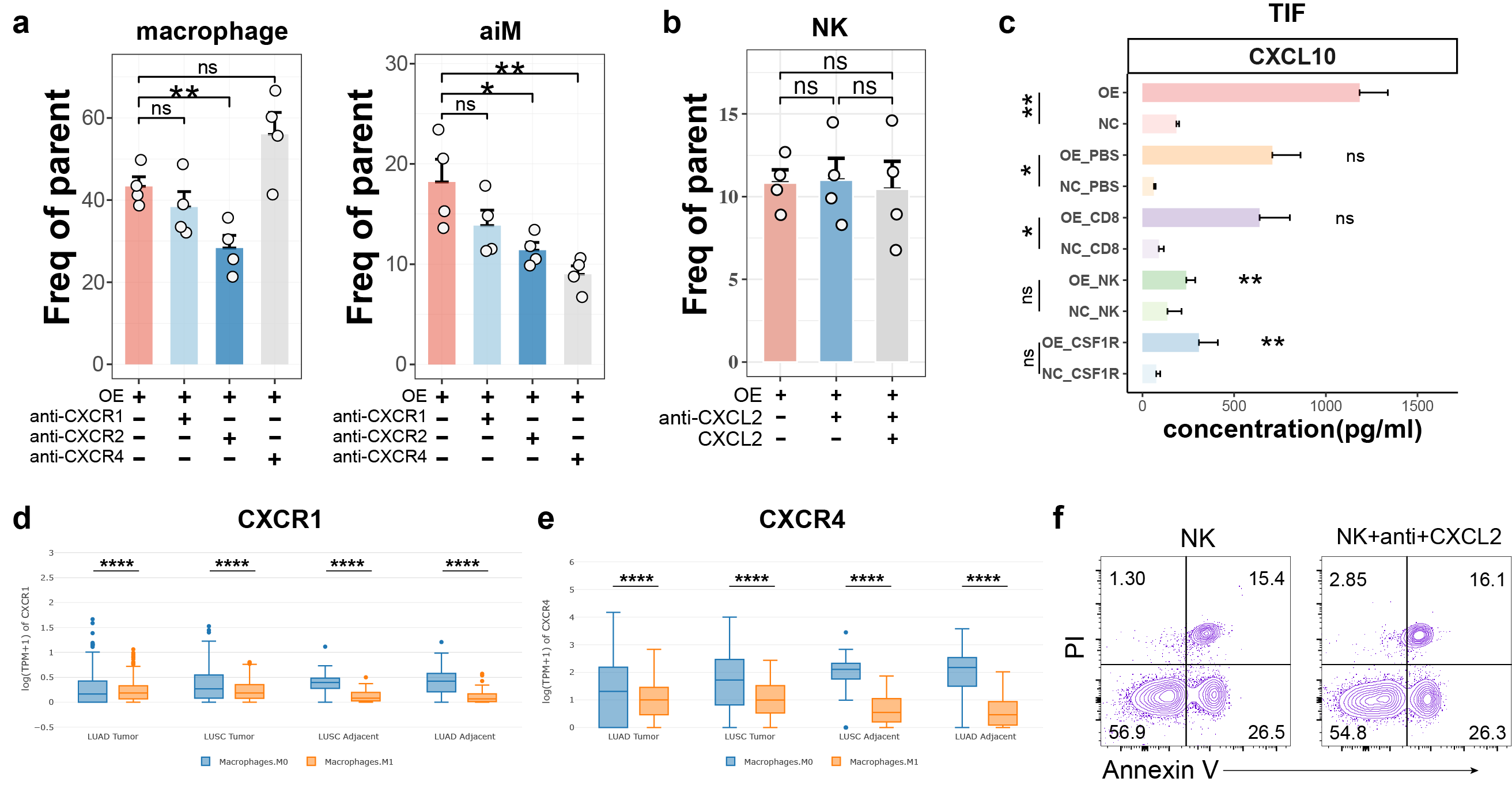


**FigS9. The exclusive receptors of CXCL2 on M1omega and its influence on NK are identified.**

The data were produced utilizing publicly-available human specimens (d) or in-house murine specimens (a-c, f).

a. Proportion of migrating macrophages and aiM in NLRP4-OE with anti-CXCR1, anti-CXCR2 and anti-CXCR4 interventions in transwell co-culture system. (n=4)

b. Proportion of migrating NK cells in NLRP4-OE with anti-CXCL2 and CXCL2 supplement in transwell co-culture system. (n=4)

c. Concentration of CXCL10 in TIF.

d. Expression of CXCR1 in M0 and M1 from TCGA LUSC and LUAD datasets.

e. Expression of CXCR4 in M0 and M1 from TCGA LUSC and LUAD datasets.

f. Representative flow cytometric analysis for MFI of Annexin V+ LLC induced by sorted NK and anti-CXCL2 supplement. (n=4)

Data represent mean ± SEM; ns p > 0.05, *p < 0.05, **p < 0.01, ***p < 0.001 ,and ****p < 0.0001 from unpaired Student’s t tests.

**Figure. S10.**


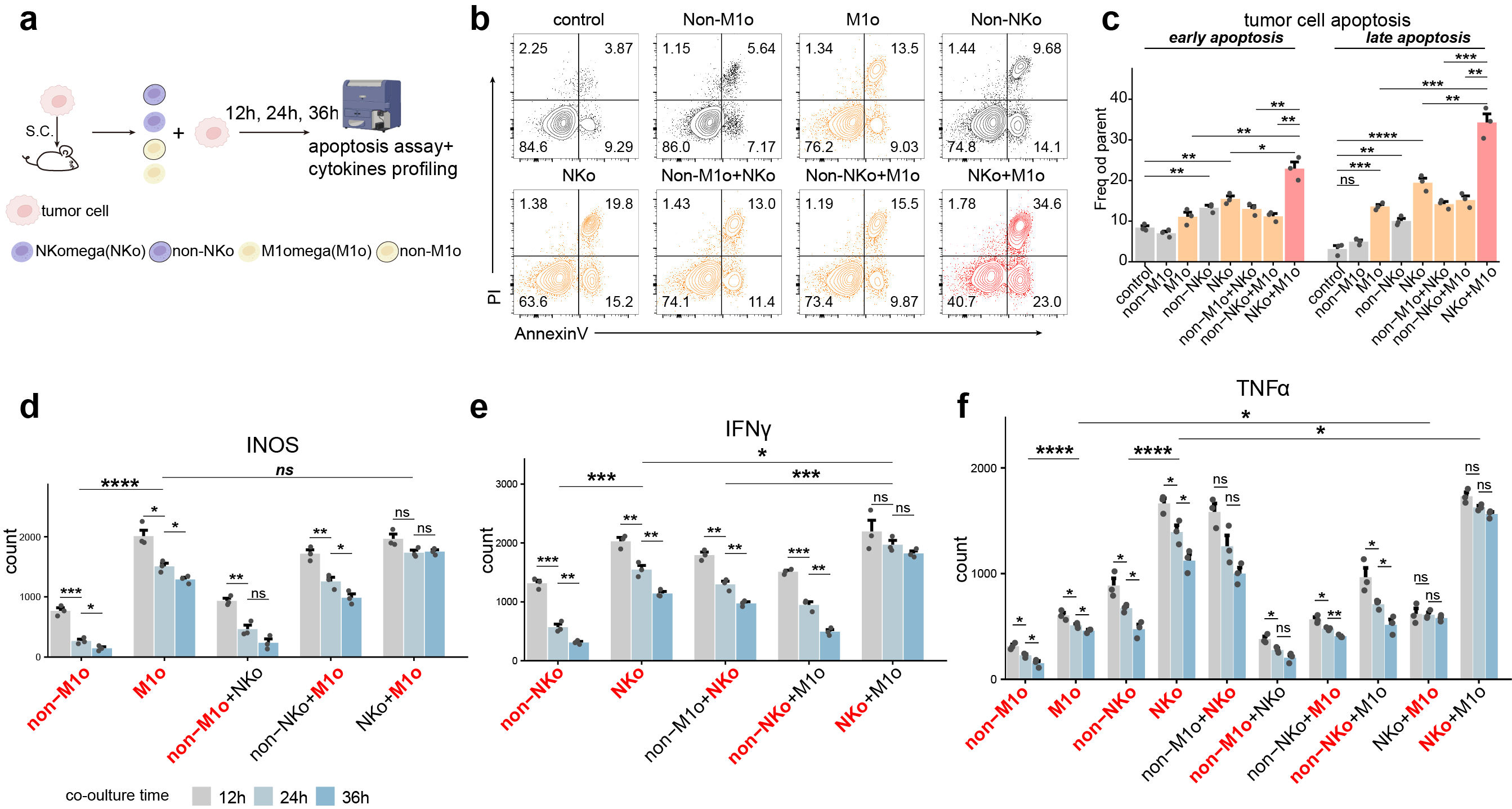


**FigS10. NKomega and M1omega synergize to amplify antitumor immunity.**

The data were produced utilizing in-house murine specimens.

a.Experimental design for apoptosis assay in b-c and cytokine profiling in d-f.

b.Representative flow cytometric analysis for MFI of Annexin V+ LLC induced by sorted M1o (M1omega), non-M1o, NKo (NKomega) non-NKo or both of them. (n=3)

c.Statistical plot for early apoptosis (gated by AnnexinV+PI-) and late apoptosis (gated by AnnexinV+PI-) in b.

d-f. Detection of INOS, IFNγ and TNFα secretion by cells marked red via flow cytometry at 12H, 24H and 36H during co-culturing.

Data represent mean ± SEM; ns p > 0.05, *p < 0.05, **p < 0.01, ***p < 0.001 ,and ****p < 0.0001 from unpaired Student’s t tests.

**Figure. S11.**

**
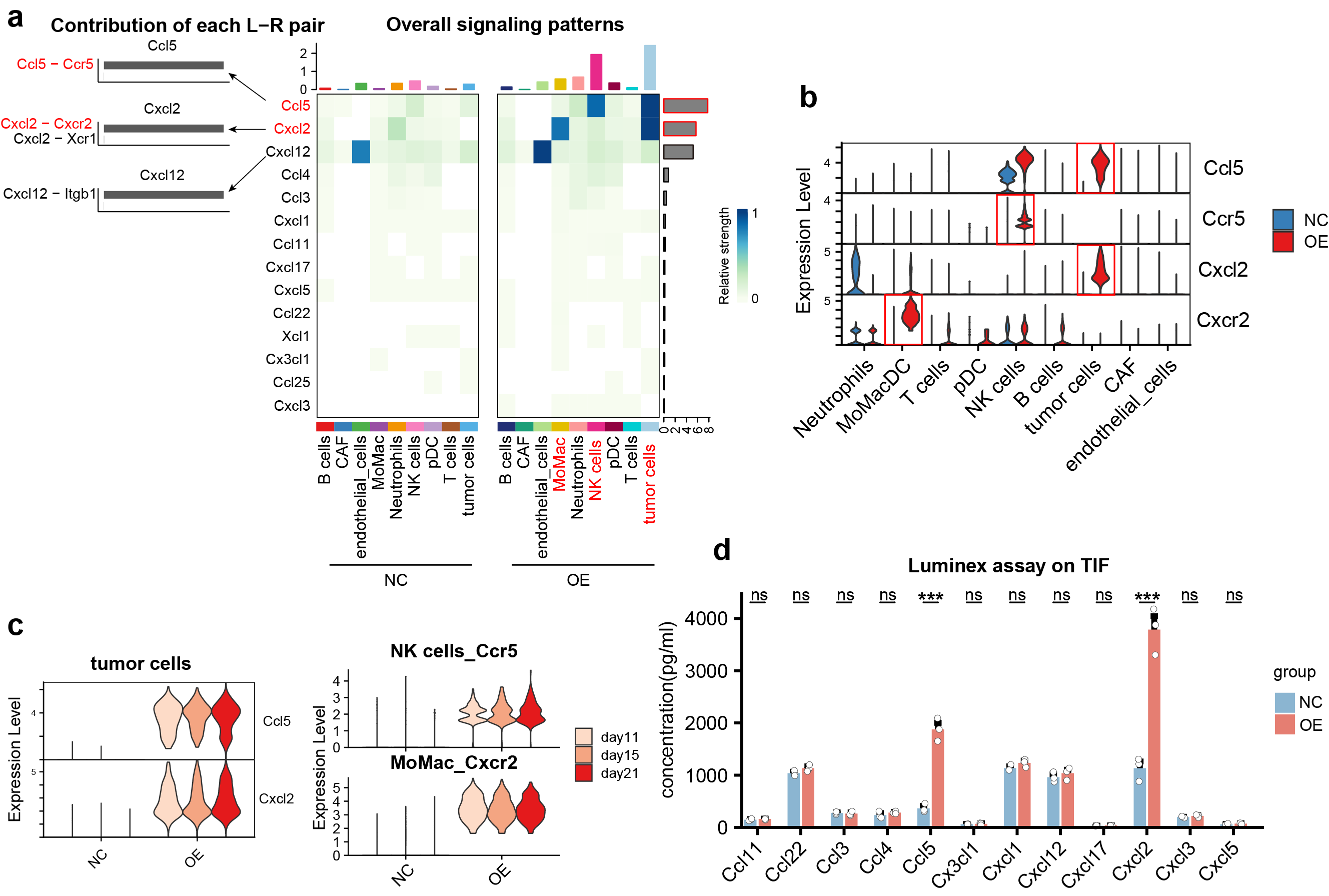
**

**FigS11. Tumor cells in NLRP4-OE TME recruits NK and M through Ccl5-Ccr5 and Cxcl2-Cxcr2, respectively.**

The data were produced utilizing in-house murine specimens.

a.The overall signaling patterns between chemokine pathways and defined cell clusters calculated by Cellchat. The interaction strength reflects the sum of all normalized interactions in each pathway. The top bar graph and the right one summarize the interaction strength per cell type or per pathway respectively. Detailed contribution of ligand-receptor pairs are labeled on the left.

b.Vlnplot showing the expression of Ccl5, Ccr5, Cxcl2, Cxcr2 across clusters in NLRP4-NC and NLRP4-OE group.

c.Vlnplot showing the expression of Ccl5 and Cxcl2 in tumor cells, Cxcr2 in MoMac and Ccr5 in NK cells in NLRP4-NC and NLRP4-OE group at different time points.

d.The concentration of chemokines in TIF from tumor tissue of mice bearing NLRP4-NC and NLRP4-OE LLC detected by Luminex.

Data represent mean ± SEM; ns p > 0.05, *p < 0.05, **p < 0.01, ***p < 0.001 ,and ****p < 0.0001 from unpaired Student’s t tests.

**Figure. S12.**


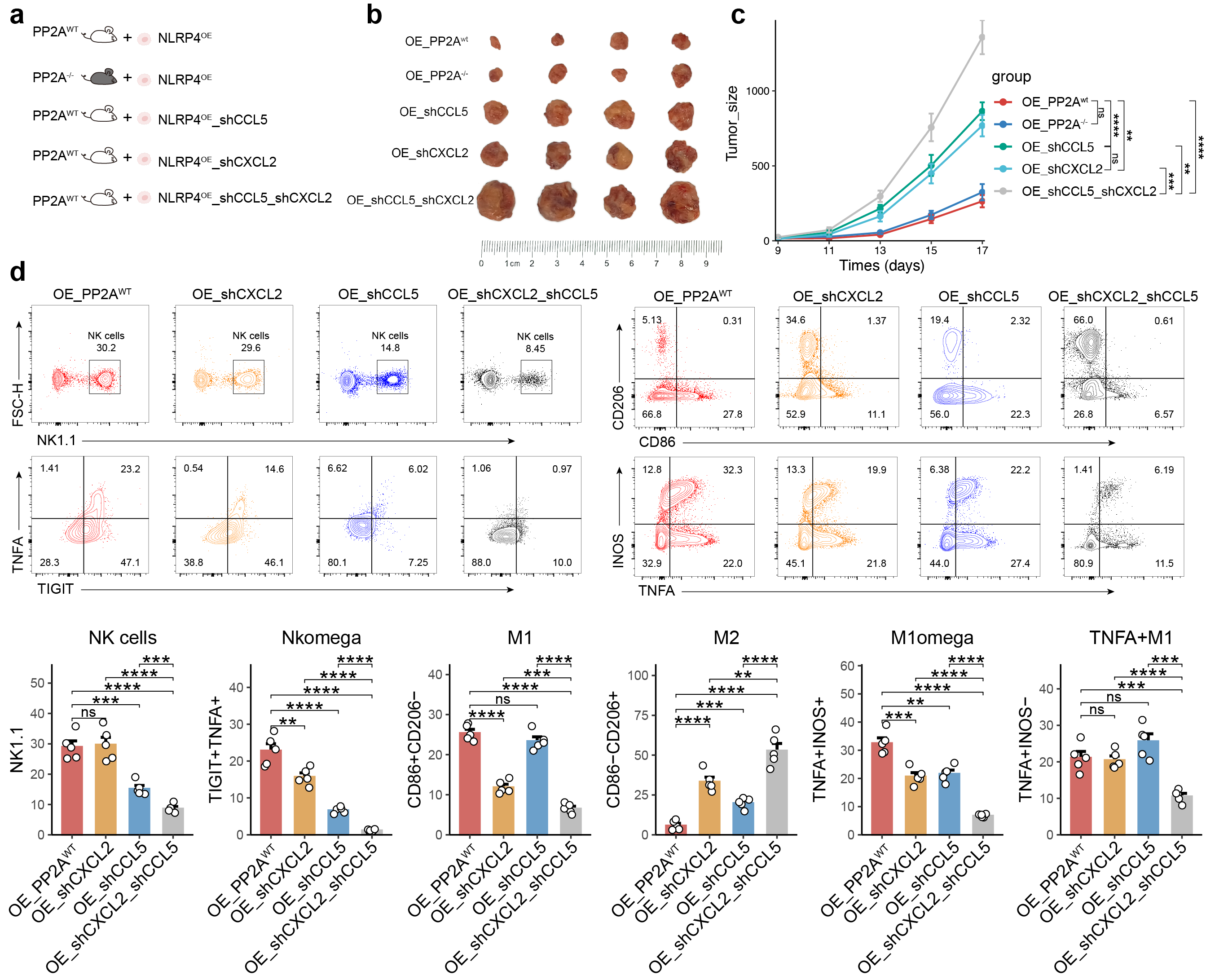


**FigS12. Tumor cells in NLRP4-OE TME recruits NK and M through Ccl5-Ccr5 and Cxcl2-Cxcr4, respectively.**

The data were produced utilizing in-house murine specimens.

a.Experimental design for animal experiments in b-d.

b.Tumors dissected from PP2A-WT or PP2A-KO mice bearing NLRP4-OE with PP2A, CCL5, CXCL2 silence or both.

c.Growth of tumors in PP2A-WT or PP2A-KO mice in b.

d.Representative flow cytometric analysis (upper) and statistical plot (lower) for NK, M1 and their subsets in the TME of mice in b.

Data represent mean ± SEM; ns p > 0.05, *p < 0.05, **p < 0.01, ***p < 0.001 ,and ****p < 0.0001 from unpaired Student’s t-test and two-way ANOVA followed by Tukey’s HSD post - hoc test for pairwise comparisons. Tumor growth was assessed using two-way ANOVA test.

**Figure. S13.**


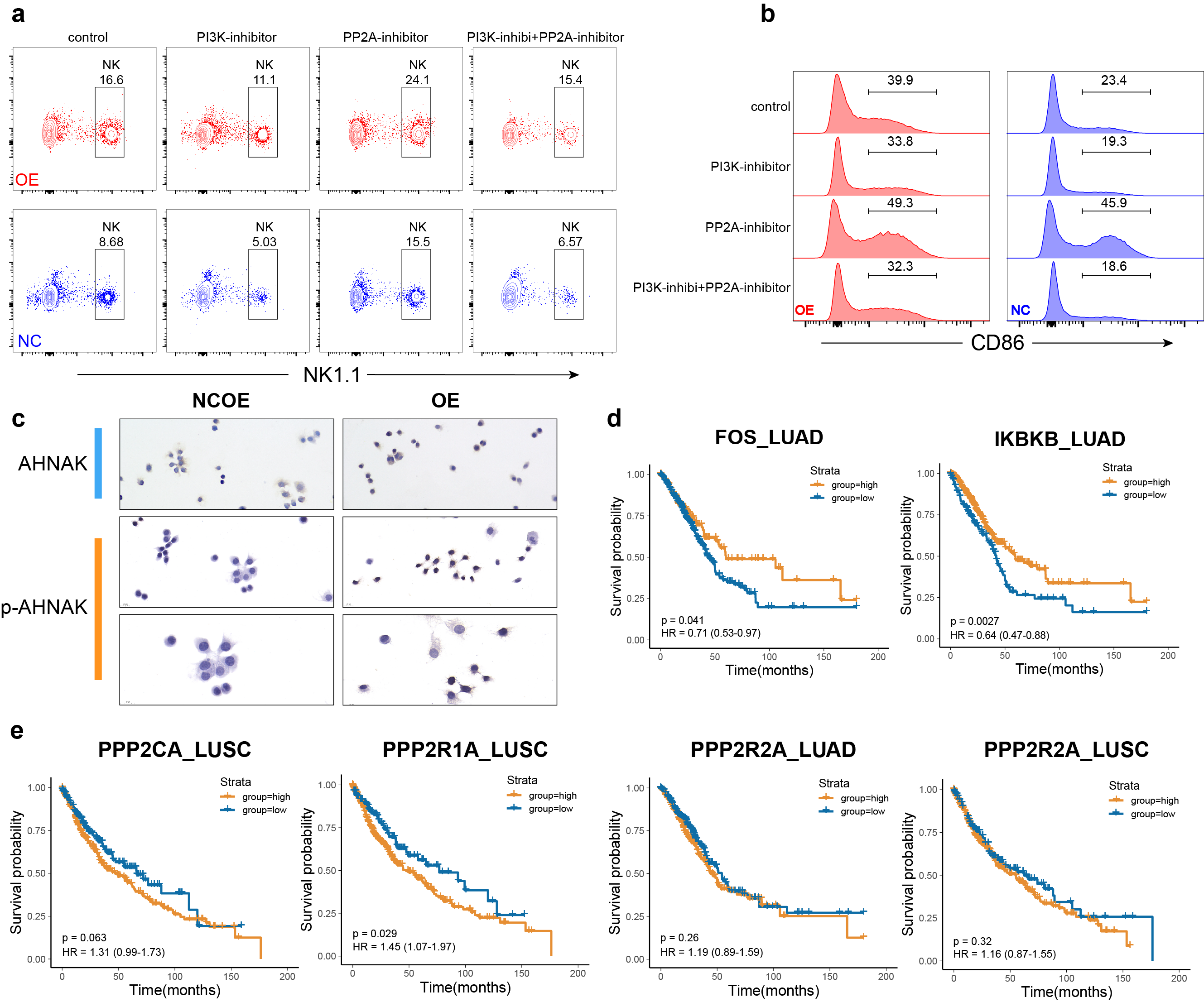


**FigS13. NKomega and M1omega are triggered by PP2A-PI3K/Akt-NF-kB axis**

The data were produced utilizing publicly-available human specimens (d-e) or in-house murine specimens (a-c).

a. Representative flow cytometric analysis for NK cells induced by NLRP4-NC and NLRP4-OE cell lines with PI3K inhibitor and PP2A inhibitor. (n=4)

b. Representative ridge plot of M1 cells induced by NLRP4-NC and NLRP4-OE cell lines with PI3K inhibitor and PP2A inhibitor. (n=4)

c. Representative immunohistochemistry (IHC) images showing the AHNAK and p-AHNAK expression pattern in Cell patch-clamping from NLRP4-NCOE and NLRP4-OE cell lines. Scale bar = 20 μm.

d. Kaplan-Meier curves analysis showing FOS and IKBKB associated with better OS in TCGA_LUAD. Two-sided log-rank test.

e. Kaplan-Meier curves analysis showing catalytic subunit of PP2A associated with better OS while regulatory subunit revealed no impact upon OS in TCGA_LUAD. Two-sided log-rank test.

The p-value of Kaplan-Meier curves was determined by log-rank test.

**Figure. S14.**


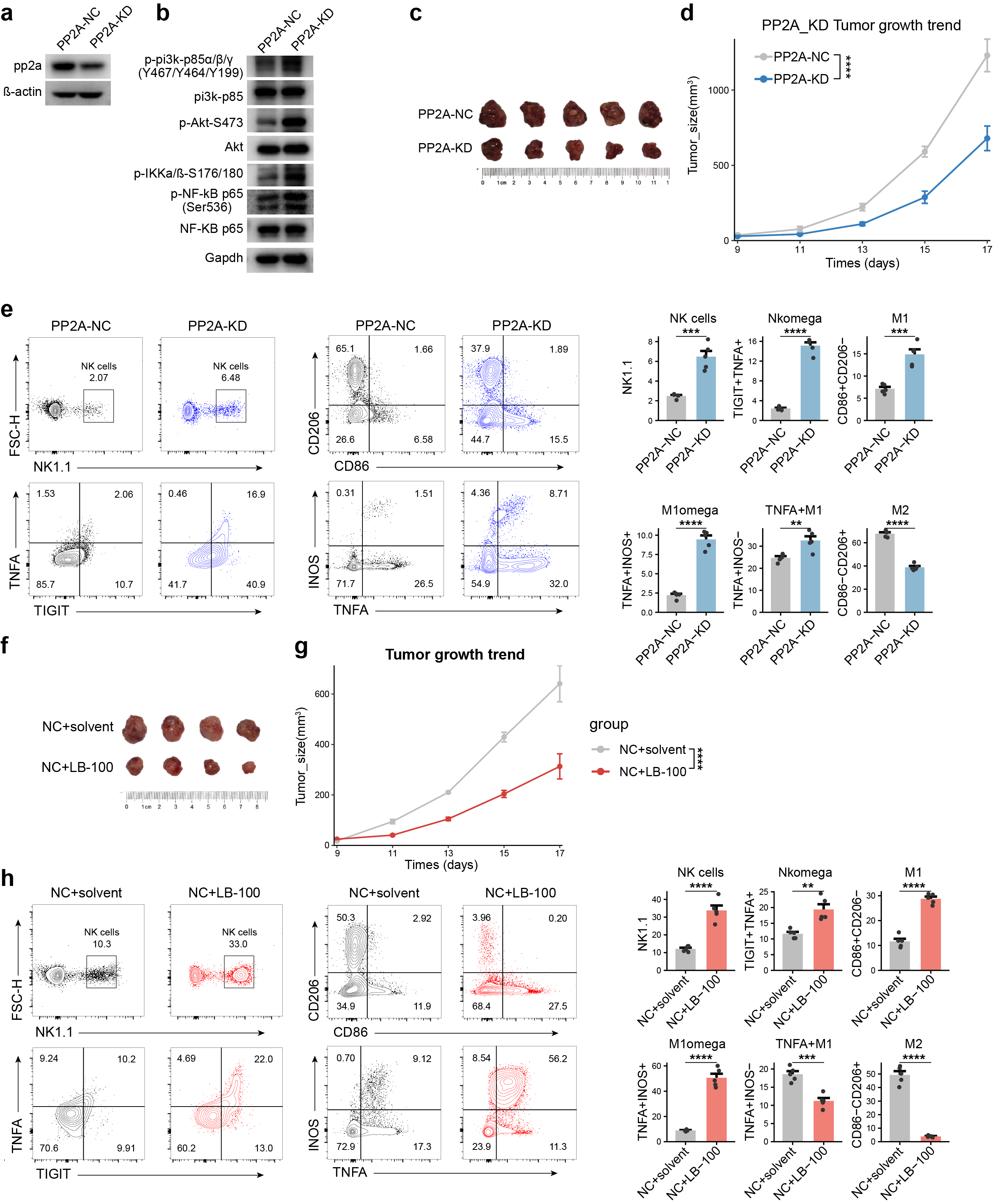


**FigS14. NLRP4-eco reconstructs its immune-promoting niche through PP2A-PI3K/Akt-NF-κB axis.**

The data were produced utilizing in-house murine specimens.

a.Confirmation of successful PP2A knockout by Western blot analysis in PP2A-NC and PP2A-KD cell lines.

b.The protein expression levels of p-PI3K, p-AKT, p-IKKαβ, p-NF-kB and p-c-Jun of PP2A-NC and PP2A-KD cell lines measured by Western blot analysis.

c.Tumors dissected from mice bearing PP2A-NC and PP2A-KD LLC cells.

d.Growth of PP2A-NC and PP2A-KD in immunocompetent C57BL/6 mice.

e.Representative flow cytometric analysis (left) and statistical plot (right) for NK, M1 and their subsets in the TME of PP2A-NC and PP2A-KD.

f.Tumors dissected from mice bearing LLC cells treated with LB-100 or solvent.

g.Growth of LLC in immunocompetent C57BL/6 mice with treatment of LB-100 or solvent.

h.Representative flow cytometric analysis (left) and statistical plot (right) for NK, M1 and their subsets in the TME of mice bearing LLC with treatment of LB-100 or solvent.

Data represent mean ± SEM; ns p > 0.05, *p < 0.05, **p < 0.01, ***p < 0.001 ,and ****p < 0.0001 from unpaired Student’s t-test and two-way ANOVA followed by Tukey’s HSD post - hoc test for pairwise comparisons. Tumor growth was assessed using two-way ANOVA test.

**Figure. S15.**

**
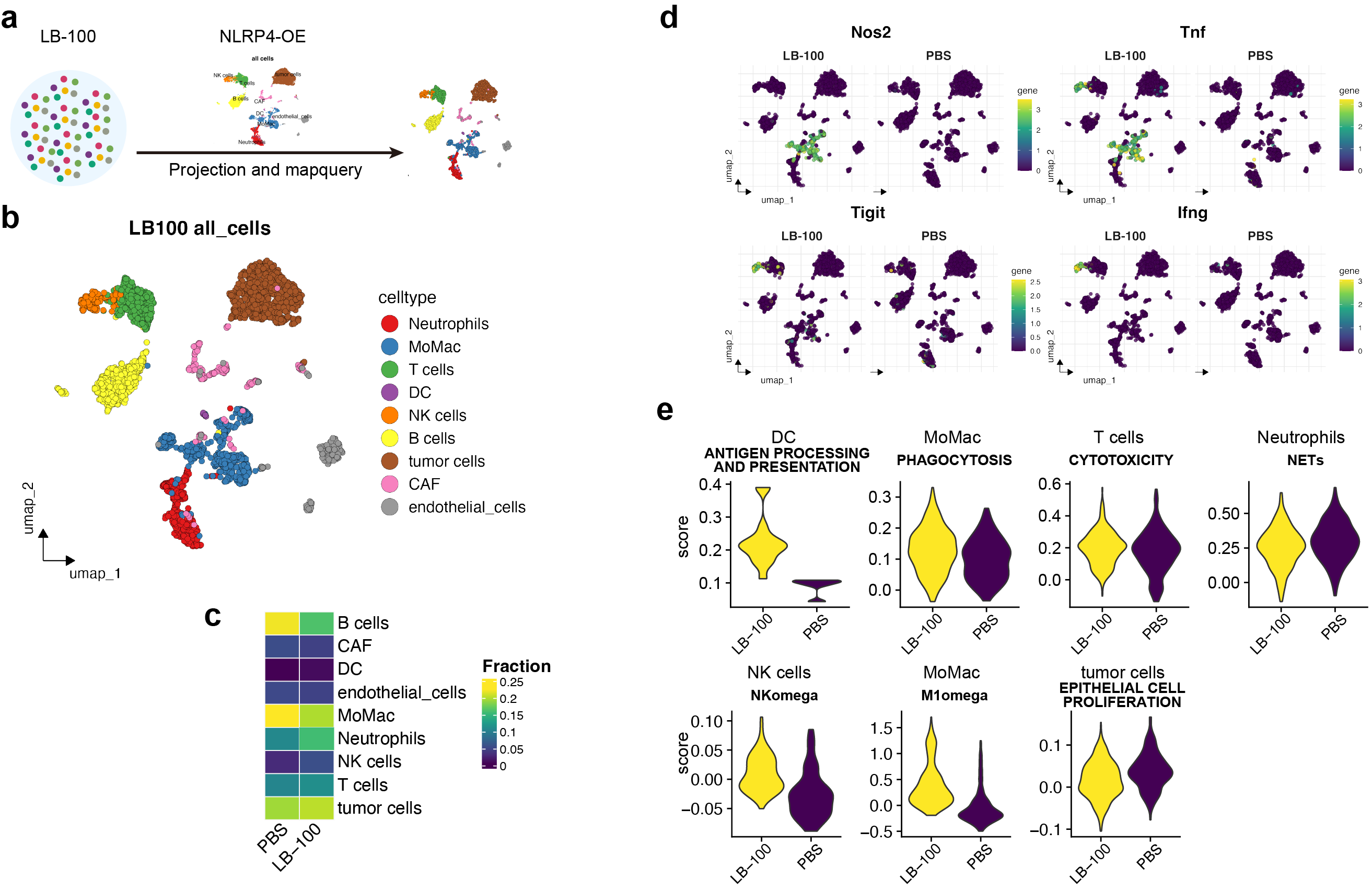
**

**FigS15. LB-100 recapitulate NLRP4's anti-tumor effects.**

The data were produced utilizing in-house murine specimens.

a.Analysis strategy for single-cell data from mouse tumor tissue treated with LB100.

b.UMAP plot of all cells colored by clusters from the tumor tissue of mice bearing LLC treated by LB-100 or PBS.

c. Heatmap showing fractions of different clusters in the LB-100 and PBS group.

d. Feature plot showing the expression distribution of Inos, Tnf, Tigit and Ifng in the LB-100 and PBS group.

e.Expression scores of different gene sets from MsigDB and PMID 35688556 across cells in the LB-100 and PBS groups

**Figure. S16.**

**
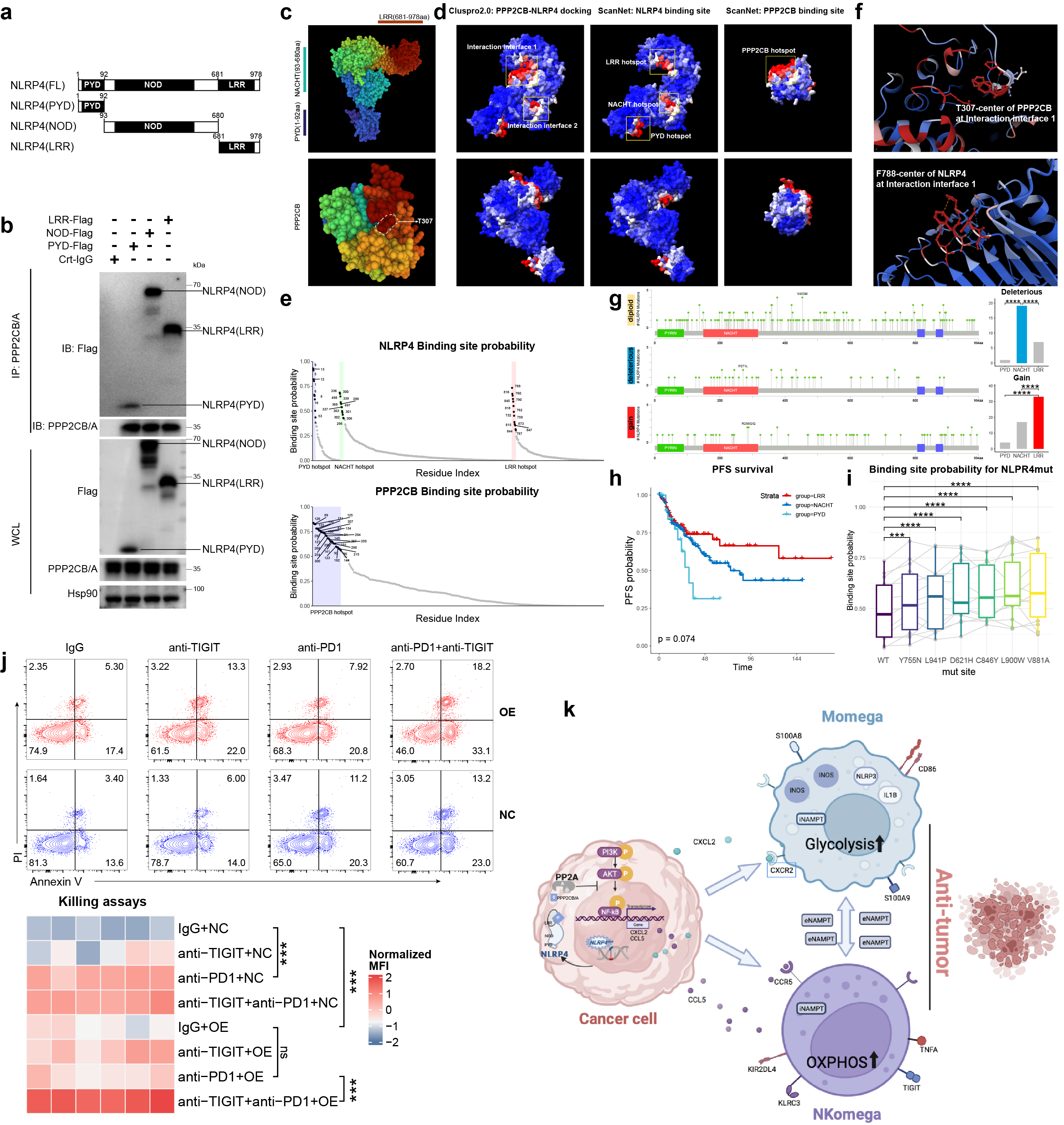
**

**FigS16: The LRR and NACHT domains of NLRP4 interacts with PP2A and anti-TIGIT and anti-PD-1 synergistically enhance the therapeutic efficacy upon NLRP4-OE.**

The data were produced utilizing publicly-available human specimens (c, d, f, e, g, h, k) or in-house murine specimens (a, b, j).

a. Fragment mode diagram of NLRP4.

b. Western blot analysis after immunoprecipitation of PP2A in HEK293T cells.

c. 3D structure of NLRP4 and PP2ACB.

d-f. Computational docking model for NLRP4 and PP2A predicted using ClusPro and ScanNet.

g. NLRP4 mutation site in diploid, deleterious and gain group (left) and statistic bar plots of them (right).

h. Survival curve showing that the LRR and NACHT alterations were associated with better OS in TCGA pan-cancer cohort.

i. Binding site probability for NLPR4mut happening to LRR segment. The p-value was determined by two-sided paired t-tests.

j. Representative flow cytometric analysis (left) and statistical plot (right) for MFI of Annexin V+ LLC with IgG, anti-TIGIT or anti-PD1 antibody. (n=4)

k. Eco-system determined by NLRP4.

Data represent mean ± SEM; ns p > 0.05, *p < 0.05, **p < 0.01, ***p < 0.001 ,and ****p < 0.0001 from paired Student’s t-tests or unpaired Student’s t-tests. The p-value of Kaplan-Meier curves was determined by log-rank test.

**Illustration:**

NLRP4 has been comprehensively characterized in the context of TBK1-mediated IFN signaling, Rho-actin signaling and BECN1-mediated autophagosome formation, all facilitated by the NOD domain. We thus questioned whether NOD domain participated in the direct interaction with PPP2CA/B either. We transfected HEK293T cells with PYD, NOD and LRR domains of NLRP4 separately and subsequently assessed their respective interaction potentials with PPP2CA/B (Method). Surprisingly, NOD and LRR both exhibited significant interactions (Fig.S16a-b). We further employed Alphaphold, ScanNet and Cluspro2.0 (Method) to predict precise binding mechanism of NLRP4 with PPP2CB, which shares 97% sequence similarity with PPP2CA and has previously been identified through co-IP/LC-MS/MS.

Interestingly, NLRP4 exhibited distinct binding regions across its three domains, each representing a binding hotspot. In contrast, PPP2CB featured a solitary binding hotspot centered around its modification site, Y307 (Fig.S16c). Of significant relevance, LRR hotspot and the complementary NOD hotspot, along with the resulting pocket structure, effectively accommodate PPP2CB, which suggested the active participation of both the LRR and NOD domains (Fig.S16d-e). Additionally, it became evident that the LRR hotspot was accountable for direct engagement with the PPP2CB hotspot (interaction interface 1). Notably, within this interface, the Y307-centered region of PPP2CB engaged in π-π interactions with the F788-centered region within the LRR binding hotspot, a distinctive interaction absent from interaction interface 2 (Fig.S16f).

Consequently, we postulated that mutations within the LRR and NOD domains could potentially exert distinct influences. Subsequent analysis of the pan-cancer TCGA atlas revealed a proclivity for gain-of-function associated with LRR mutations, while mutations within the NOD domain displayed a predilection for deleterious effects (Fig.S16g). Additionally, disparate OS prognoses were observed for mutations occurring within the PYD, NOD and LRR domains (Fig.S16h). Remarkably, functional predictions corroborated these findings, underscoring that mutations within the LRR domain engender an augmented binding strength at interaction interface 1 (Fig.S16i). In aggregate, we concluded that PPP2CB engages in direct interaction with LRR and NOD domains of NLRP4, wherein the former prominently assumes the role of facilitating hotspot-mediated direct interactions.

Meanwhile, anti-PDL1-mediated up-regulation of TIGIT expression on NK prompted us to consider whether anti-TIGIT could further liberate NKomega to rescue ineffectiveness of anti-PDL1. Indeed, combination of anti-TIGIT with anti-PDL1 strengthened tumor-suppression behavior of NLRP4-OE intensively, compared to anti-PDL1 alone (Fig.S16j).

**Figure. S17.**


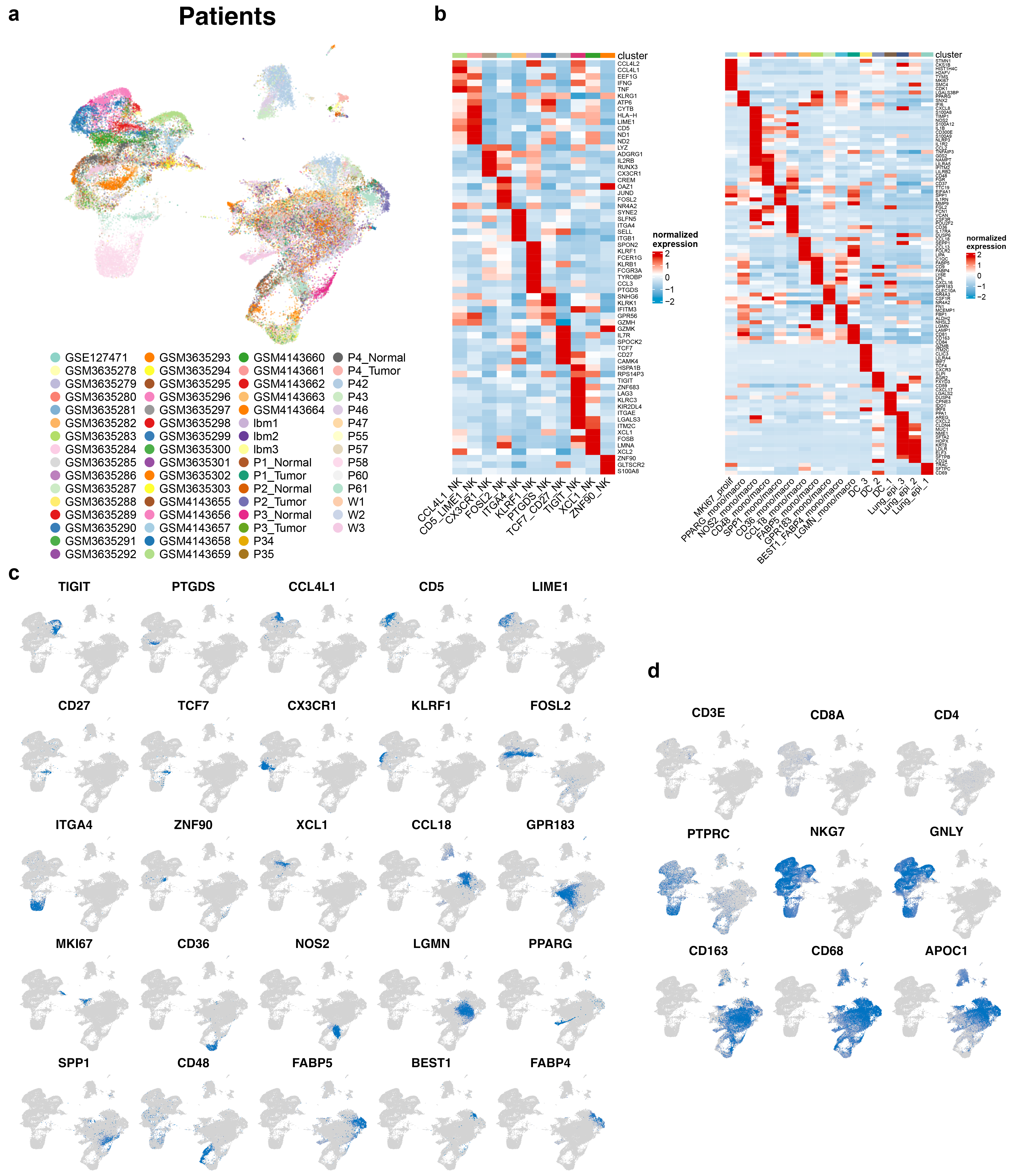


**FigS17. Single-cell RNA sequencing annotates immune cell clusters and reveals their feature genes.**

The data were produced utilizing publicly-available human specimens.

a. UMAP plot of 50,215 cells colored by patients in an integrated cohort.

b. Heat map showing marker genes in each cluster.

c. UMAP feature plots showing the expression levels of certain genes.

d. UMAP feature plots showing the expression levels of certain genes.

Owing to substantial cell count (50,215) and meticulous integration, we managed to distinguish 11 NK subsets and 10 M subsets (Fig.S17b-c), employing comprehensive literature-based artificial annotations (Table S5), with elimination of any T cells contamination beforehand (Fig.6a, S17d).

**Illustration:**

Surprisingly, TIGIT^+^ NK not only revealed extensive expression of activation (KLRC3) and cytoxicity markers (KIR2DL4)[1], but also displayed a stress-responding phenotype[2] (HSPA1B) accompanied by tissue-resident characteristics (ITGAE)[3] (Fig.6b). Sunwoo et al. have identified a similar ITGA1^+^ITGAE^+^ NK subset[4], which possessed the greatest CD107A and IFNY secretion capacity in HNSCC. To further elucidate its characteristics, we employed a progressively-narrowing monocle2 (n-monocle, Method) approach (Fig.S18) to ultimately isolate and analyze two distinct NK subsets: TIGIT^+^ NK and CCL4L2^+^ NK, which were previously considered as indistinguishable cytotoxic anti-tumor subsets in metastatic melanoma[5] and exhibited similar differentiation trajectory from CX3CR1^+^ NK here as well (Fig.6c-d). Interestingly, with high-resolution monocle analysis, we identified a distinct differentiation state of TIGIT^+^ NK. TIGIT^+^ NK co-expressed multiple exhaustion markers, including PDCD1, LAG3 and CTLA4, together with significant up-regulation of cytotoxic molecules, including GZMA/B, IFNY and KIR2DL4, which was exactly the same as NKomega (Fig.6e). On the contrary, CCL4L2^+^ NK manifested a pro-inflammatory secretion state[6], with enhanced expression of CCL4, S1PR1 and ITGB2 (Fig.6e).

NOS2^+^ M1 largely resembled NLRP3^+^ M and IL1B^+^ M defined by Zhang et al[7]. NLRP3^+^ M was represented as pro-inflammatory tissue-resident M (TRMs), and IL1B^+^ M could drive anti-tumor capacity through IL1B-ADRB2 interaction with TNF^+^ mast cells[7]. NOS2^+^ M1 expressed elevated level of calprotectin (S100A8/9) as well (Fig.6f), which served as endogenous agonist of TLR4, initiating inflammation cascade in M1[8].

More importantly, gse25123_wt_vs_pparg_ko_macrophage_up pathway was significantly enriched in NOS2^+^ M1 (Fig.6g). PPARγ pathway was indispensable for protumor-inflammatory polarization through induction of fatty-acid-oxidation (FAO)[9]. Meanwhile, we did identify a PPARG^+^ M subset in our NK/M atlas. To further verify anti-tumor characteristics of NOS2^+^ M versus others, we utilized literature-derived signatures (Table S5) to elucidate them. Astonishingly, classical M1 signature ClassicalTIMs was prominently up-regulated in NOS2^+^ M1, while IFN-TAMs, which manifested immunosuppressive behavior dependent upon tryptophan degradation, was exclusively enriched in PPARG^+^ M1 (Fig.6h). Meanwhile, M1omega signature (Table S3) developed by us indeed peaked in NOS2^+^ M, surpassing PPARG^+^ M and SPP1^+^ M (Fig.6i).

**Figure. S18.**


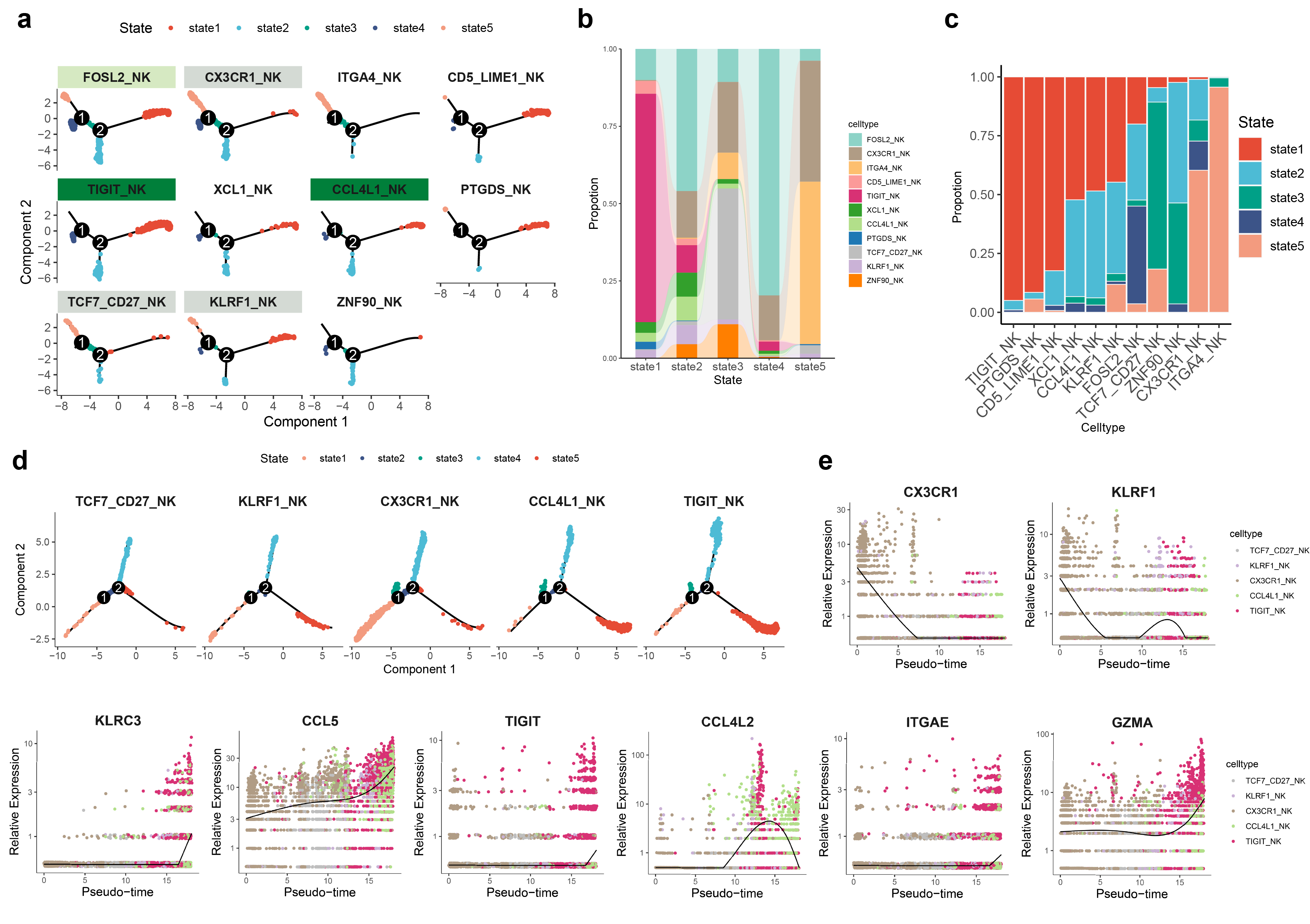


**FigS18. TIGIT^+^ NK is isolated and analyzed by n-monocle.**

The data were produced utilizing publicly-available human specimens.

a. Monocle trajectory inference of all subpopulations of NK cells, colored by their corresponding cell states.

b. Proportion of subpopulations of NK cells in each cell state.

c. Proportion of cell states in each subpopulation.

d. Monocle trajectory inference of certain subpopulations of NK cells, colored by their corresponding cell states.

e. Expression dynamics of representative genes in clusters (color coded), along the pseudotime.

**Figure. S19.**


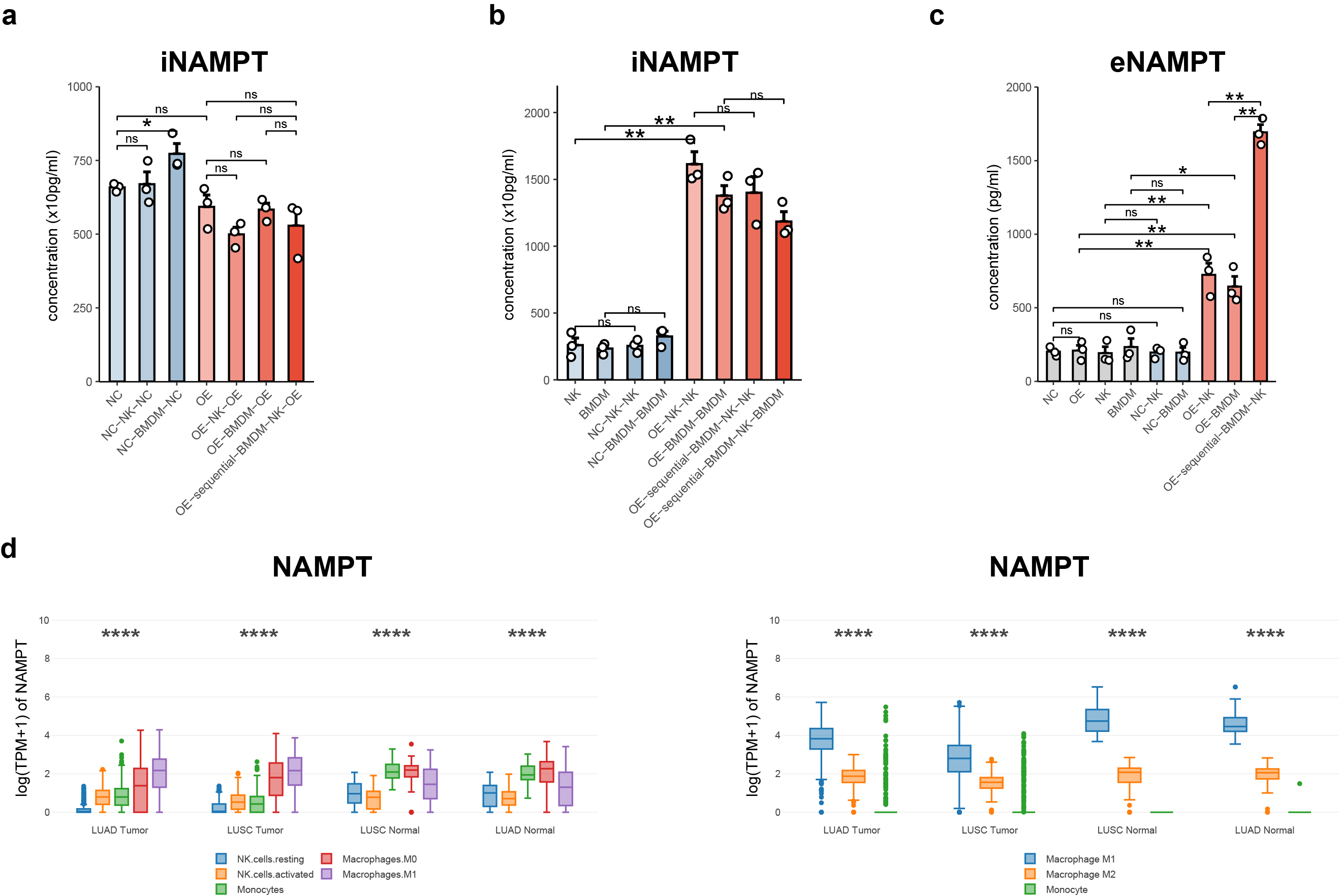


**FigS19. eNAMPT experiences substantial up-regulation.**

The data were produced utilizing publicly-available human specimens (b) or in-house murine specimens (a).

1. c. Concentration of NAMPT detected by ELISA.

d. Expression of NAMPT in NK and macrophages from TCGA LUSC and LUAD datasets.

Data represent mean ± SEM; ns p > 0.05, *p < 0.05, **p < 0.01, ***p < 0.001 ,and ****p < 0.0001 from unpaired Student’s t tests and one-way ANOVA test.

**Figure. S20.**


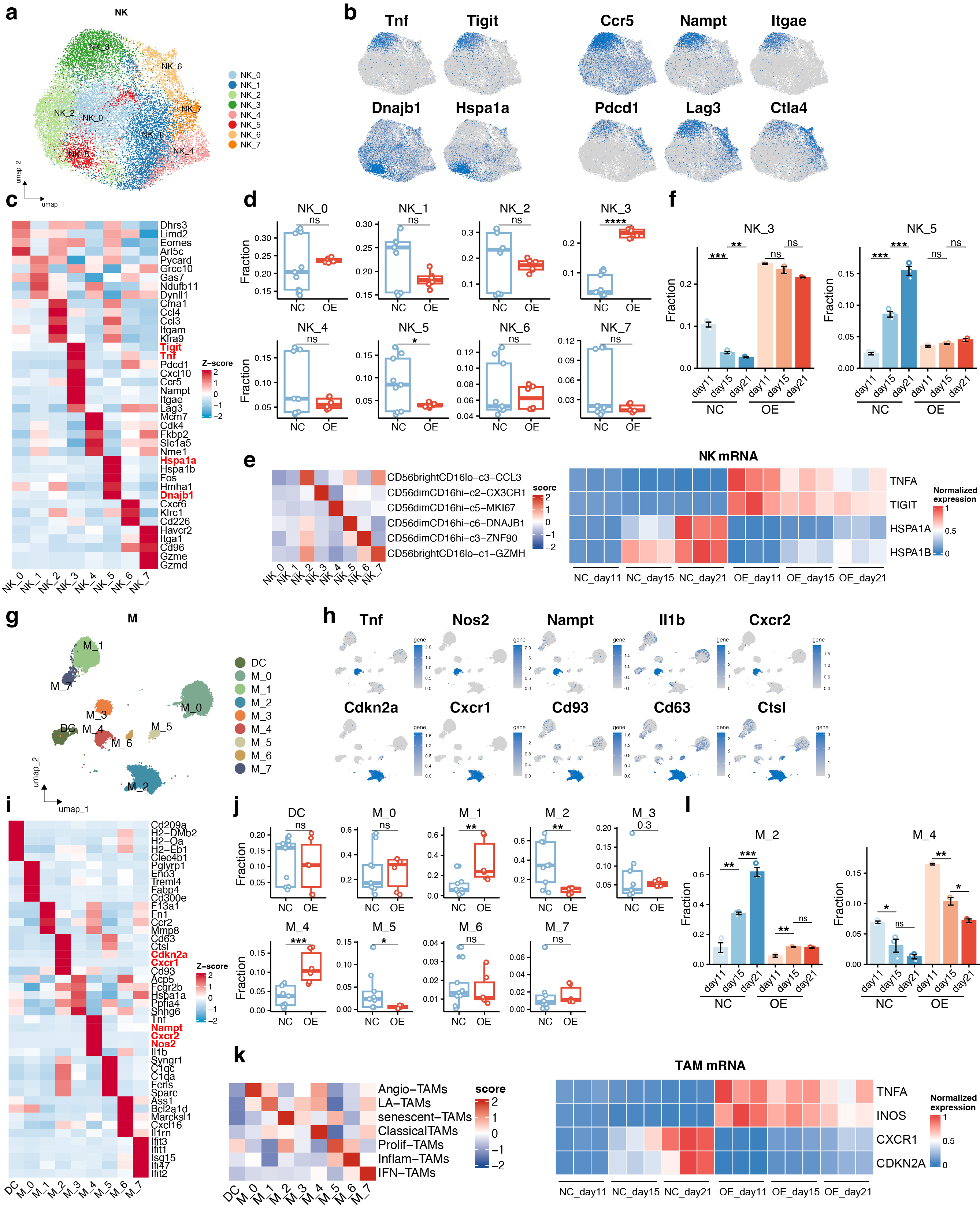


**FigS20. NLRP4-OE selectively recruits M1omega and NKomega cells, both expressing NAMPT.**

The data were produced utilizing in-house murine specimens.

a.UMAP plot of NK cells separated from all cells colored by subpopulations from the tumor tissue of mice bearing NLRP4-NC and NLRP4-OE at day11, day15 and day21.

b.Featureplot showing the expression and distribution of several genes across NK cells.

c.Heatmap showing the marker genes of NK cells’ subpopulations.

d.Fractions of subpopulations in NLRP4-NC and NLRP4-OE group.

e.Heatmap showing the association between our NK subpopulations and those from Prof.Zhang (PMID:37607536).

f.Fractions of NK_3 and NK_5 in NLRP4-NC and NLRP4-OE group at different time point (upper). Their marker genes were validated by qPCR through flow cytometry-sorted NK cells from tumor tissues at different time points (lower).

g.UMAP plot of MoMac cells separated from all cells colored by subpopulations from the tumor tissue of mice bearing NLRP4-NC and NLRP4-OE at day11, day15 and day21.

h.Featureplot showing the expression and distribution of several genes across MoMac.

i.Heatmap showing the marker genes of MoMac’ subpopulations.

j.Fractions of subpopulations in NLRP4-NC and NLRP4-OE group.

k.Heatmap showing the association between MoMac subpopulations and those from literature.

l.Fractions of M_2 and M_4 in NLRP4-NC and NLRP4-OE group at different time point (upper). Their marker genes were validated by qPCR through flow cytometry-sorted macrophages from tumor tissues at different time points (lower).

Data represent mean ± SEM; ns p > 0.05, *p < 0.05, **p < 0.01, ***p < 0.001 ,and ****p < 0.0001 from unpaired Student’s t tests.

**Figure. S21.**

**
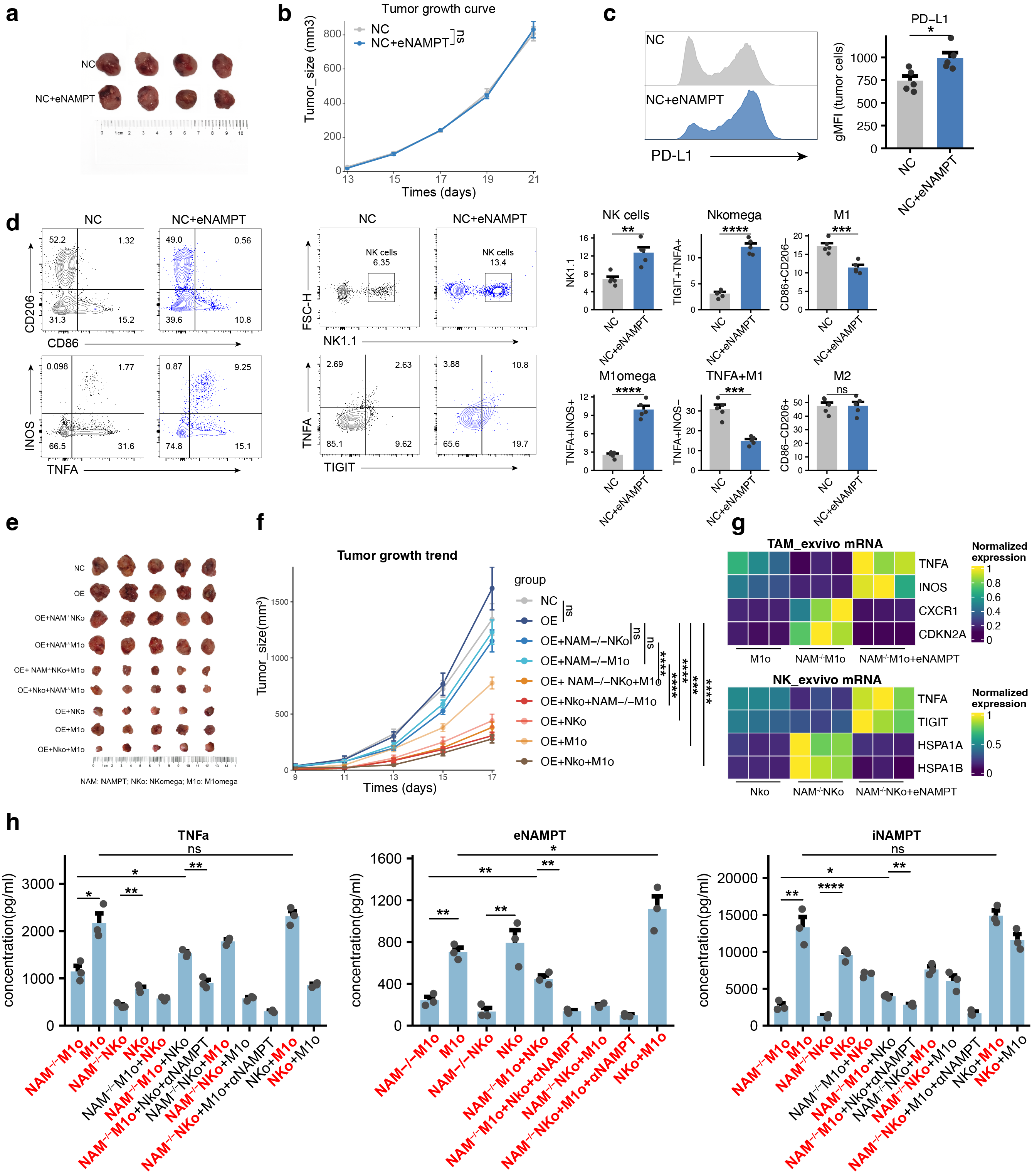
**

**FigS21. NAMPT supports the anti-tumor activities of NKomega and M1omega in NLRP4-eco and acts as a communication bridge between them.**

The data were produced utilizing in-house murine specimens.

a.Tumors dissected from mice bearing LLC with injecting eNAMPT intratumorally or not.

b.Tumor growth curve measured in a.

c.Representative ridge plot for the MFI of PD-L1 expression in tumor cells from a (left) and statistical plot of them (right).

d.Representative flow cytometric analysis (left) and statistical plot (right) for NK, M1 and their subsets in the TME of mice in b.

e.Tumors dissected from mice bearing NLRP4-NC or NLRP4-OE LLC companied by adoptive cell therapy (ACT) with NKomega and M1omega cells, separated from Nampt KO mice or WT mice.

f.Tumor growth curve measured in f.

g.The mRNA expression of marker genes from FigureR7 in TAM and NK sorted by flow cytometry, with eNAMPT or not.

h.Detection of TNFα, eNAMPT and iNAMPT secretion by cells marked red via ELISA.

Data represent mean ± SEM; ns p > 0.05, *p < 0.05, **p < 0.01, ***p < 0.001 ,and ****p < 0.0001 from unpaired Student’s t-test and two-way ANOVA followed by Tukey’s HSD post - hoc test for pairwise comparisons. Tumor growth was assessed using two-way ANOVA test.

**References**

1. Zheng G, Guo Z, Li W, Xi W, Zuo B, Zhang R, et al. Interaction between HLA-G and NK cell receptor KIR2DL4 orchestrates HER2-positive breast cancer resistance to trastuzumab. Signal Transduct Target Ther. 2021;6:1–15.

2. Chu Y, Dai E, Li Y, Han G, Pei G, Ingram DR, et al. Pan-cancer T cell atlas links a cellular stress response state to immunotherapy resistance. Nat Med. 2023;29:1550–62.

3. Franklin M, Connolly E, Hussell T. Recruited and Tissue-Resident Natural Killer Cells in the Lung During Infection and Cancer. Front Immunol. 2022;13:887503.

4. Moreno-Nieves UY, Tay JK, Saumyaa S, Horowitz NB, Shin JH, Mohammad IA, et al. Landscape of innate lymphoid cells in human head and neck cancer reveals divergent NK cell states in the tumor microenvironment. Proc Natl Acad Sci U S A. 2021;118:e2101169118.

5. Andrade LF de, Lu Y, Luoma A, Ito Y, Pan D, Pyrdol JW, et al. Discovery of specialized NK cell populations infiltrating human melanoma metastases. JCI Insight. 2019;4:e133103.

6. Bourayou E, Golub R. Inflammatory-driven NK cell maturation and its impact on pathology. Front Immunol. 2022;13:1061959.

7. Cheng S, Li Z, Gao R, Xing B, Gao Y, Yang Y, et al. A pan-cancer single-cell transcriptional atlas of tumor infiltrating myeloid cells. Cell. 2021;184:792-809.e23.

8. Bosch MH van den, Blom AB, Schelbergen RF, Koenders MI, Loo FA van de, Berg WB van den, et al. Alarmin S100A9 Induces Proinflammatory and Catabolic Effects Predominantly in the M1 Macrophages of Human Osteoarthritic Synovium. J Rheumatol. 2016;43:1874–84.

9. Liu S, Zhang H, Li Y, Zhang Y, Bian Y, Zeng Y, et al. S100A4 enhances protumor macrophage polarization by control of PPAR-γ-dependent induction of fatty acid oxidation. J Immunother Cancer. 2021;9:e002548.
